# α-Glucosidase Inhibitors from Two Mangrove-Derived Actinomycetes

**DOI:** 10.3390/molecules28093822

**Published:** 2023-04-29

**Authors:** Xuejun Lu, Manlai Zhang, Yixian Qiu, Xiuxiu Liu, Cancan Wang, Jianwei Chen, Huawei Zhang, Bin Wei, Yanlei Yu, Youmin Ying, Kui Hong, Hong Wang

**Affiliations:** 1 College of Pharmaceutical Science & Collaborative Innovation Center of Yangtze River Delta Region Green Pharmaceuticals, Zhejiang University of Technology, Hangzhou 310014, China; 2 Key Laboratory of Combinatorial Biosynthesis and Drug Discovery, Ministry of Education, School of Pharmaceutical Sciences, Wuhan University, Wuhan 430072, China; 3 Key Laboratory of Marine Fishery Resources Exploitment and Utilization of Zhejiang Province, Zhejiang University of Technology, Hangzhou 310014, China

**Keywords:** mangrove microorganism, *Streptomyces*, diabetes mellitus, alpha-glucosidase, geldanamycin, diketopiperazine

## Abstract

α-Glucosidase (AGS) inhibitors have been regarded as an ideal target for the management of type 2 diabetes mellitus (T2DM) since they can maintain an acceptable blood glucose level by delaying the digestion of carbohydrates and diminishing the absorption of monosaccharides. In the process of our endeavor in mining AGS inhibitors from natural sources, the culture broth of two mangrove-derived actinomycetes *Streptomyces* sp. WHUA03267 and *Streptomyces* sp. WHUA03072 exhibited an apparent inhibitory activity against AGS. A subsequent chemical investigation into the two extracts furnished 28 secondary metabolites that were identified by spectroscopic methods as two previously undescribed linear polyketides **1**–**2**, four benzenoid ansamycins **3**–**6**, fourteen cyclodipeptides **7**–**18**, one prenylated indole derivative **19**, two fusicoccane-type diterpenoids **20**–**21**, two hydroxamate siderophore **22**–**23**, and five others **24**–**28**. Among all of the isolates, **11** and **24** were obtained from actinomycetes for the first time, while **20**–**21** had never been reported to occur in a marine-derived microorganism previously. In the in vitro AGS inhibitory assay, compounds **3**, **8**, **9**, **11**, **14**, **16**, and **17** exhibited potent to moderate activity with IC_50_ values ranging from 35.76 ± 0.40 to 164.5 ± 15.5 μM, as compared with acarbose (IC_50_ = 422.3 ± 8.4 μM). The AGS inhibitory activity of **3**, **9**, **14**, **16**, and **17** was reported for the first time. In particular, autolytimycin (**3**) represented the first ansamycin derivative reported to possess the AGS inhibitory activity. Kinetics analysis and molecular docking were performed to determine the inhibition types and binding modes of these inhibitors, respectively. In the MTT assay, **3**, **8**, **9**, **11**, **14**, **16**, and **17** exhibited no apparent cytotoxicity to the human normal hepatocyte (LO2) cells, suggesting satisfactory safety of these AGS inhibitors.

## 1. Introduction

Diabetes mellitus (DM) is a metabolic disorder featuring abnormally elevated blood glucose levels. DM has been regarded as a global health issue since its prevalence is rising at a surprisingly high rate. In total, 642 million people are expected to suffer from DM by the year 2040 [1]. Hyperglycemia has been evidenced to play a pivotal role in the onsets, development, and progress of DM. Consistent hyperglycemia can lead to a variety of complications such as foot ulcers, diabetic retinopathy, nephropathy, cardiovascular diseases, stroke, and neuropathy [2]. Hence, many interventions aimed at controlling hyperglycemia in DM patients have been developed, including insulin injection for type 1 diabetes mellitus (T1DM) and non-insulin medications for type 2 diabetes mellitus (T2DM) via reversing the pathophysiological abnormalities that contribute to hyperglycemia [2].

α-Glucosidases (AGSs) are hydrolases that cleavage the α-glucopyranosidic bond in complex carbohydrates to release glucose and other monosaccharides, resulting in elevated blood sugar levels [3]. AGS inhibitors (AGSIs) can inhibit the digestion of carbohydrate by occupying the binding site of AGS and slowing down the production and intestinal absorption of glucose [3]. Therefore, one of the effective therapeutic strategies to control hyperglycemia is the use of AGSIs. AGSIs have been regarded as the most eminent hyperglycemia controlling agents along with other medications such as glucagon-like peptide 1 (GLP-1) receptor agonists, dipeptidyl peptidase-4 (DPP-4) inhibitors, and sodium-glucose cotransporter-2 (SGL-2) inhibitors. In addition to the demand for parenteral administration, GLP-1 receptor agonists usually have short half-life times and expensive prices. DPP-4 inhibitors may cause adverse reactions such as neurogenic inflammation, elevated blood pressure, and immune responses triggering effects [4], while SGL-2 inhibitors were speculated to be related with nasopharyngitis and genitourinary infections [5]. The combinational use of AGS inhibitors with insulin, metformin, and sulfonylureas is an intervention for uncontrolled hyperglycemia recommended by the International Diabetes Federation [6]. Currently, acarbose, miglitol, voglibose, and emiglitate are commercially available AGS inhibitors. Nevertheless, the long-term use of these drugs has been reported to cause various side effects in clinical practice [5]. Hence, researchers are still endeavored in developing novel AGS inhibitors. Many synthetic efforts have been made towards the development of antidiabetic agents. In particular, progress has been achieved in the synthesis and development of iminosugars and sugar derivatives as antidiabetic agents [7,8,9].

Natural products have been regarded as a rich source of novel AGS inhibitors. The past several decades have witnessed the discovery of many AGS inhibitors from medicinal plants, including terpenes, alkaloids, quinines, flavonoids, xanthans, phenols, phenylpropanoids, steroids, and others [10,11,12,13,14]. In 2021, Dirir et al. reviewed the AGS inhibitory activity of 290 plant-derived natural products discovered between the year 2015 and 2020 and identified eight molecules, i.e., taxumariene F, akebonoic acid, morusin, rhaponticin, procyanidin A2, alaternin, mulberrofuran K, and psoralidin, as potent AGS inhibitors and promising drug candidates for the treatment of T2DM [15]. In recent years, there has been a growing interest in exploring microbial secondary metabolites for novel AGS inhibitors [16], given that the commercialized and pioneer AGS inhibitors, i.e., acarbose, voglibose, and miglitol, originated either directly or indirectly from microorganisms [17]. While mining microorganisms for novel AGS inhibitors, those inhabiting unique biotopes have been gaining attention since they were believed to produce secondary metabolites with novel structures and diverse bioactivities. Mangrove forests refer to a group of salt-tolerant plants growing at the junction of land and sea in tropical and subtropical intertidal estuarine zones [18]. In addition to the woody plants, the mangrove forests provide habitats for animals and a diverse array of microorganisms including fungi, actinomyces, bacteria, cyanobacteria, microalgae, macroalgae, and protozoa [19]. These living organisms, along with the abiotic factors, form the mangrove forest ecosystems with special ecological environments including high salinity, high tidal range, high temperature, intensive sunlight, low oxygen, and limited nutrient [20]. Microorganisms living in the mangrove forest ecosystems may evolve to form adaptational metabolic pathways and produce multifunctional secondary metabolites in response to the selective pressure posed by the unique environmental conditions. As promoted by the rise of mining novel drug leads from the big blue, along with the renaissance in the study of microbial secondary metabolites, mangrove microorganisms are becoming hot spots for microbial resources collection, secondary metabolites identification, and biosynthetic mechanism investigation [21]. The past few decades have witnessed the discovery of a variety of natural products with intriguing structures and encouraging bioactivities from mangrove associated microorganisms that are attracting attention as an inexhaustible source of drug leads [18,19,20,21].

As part of our ongoing efforts in exploring microbial natural products for AGS inhibitors [22,23,24,25,26,27,28], the ethyl acetate extracts of two mangrove-derived actinomycetes *Streptomyces* sp. WHUA03072 and *Streptomyces* sp. WHUA03267 exhibited apparent inhibitory activity. Subsequent chemical investigations led to the isolation and identification of 28 secondary metabolites. Herein, we report the isolation, structural elucidation, and biological evaluation of these compounds.

## 2. Results and Discussion

**Figure 1:**
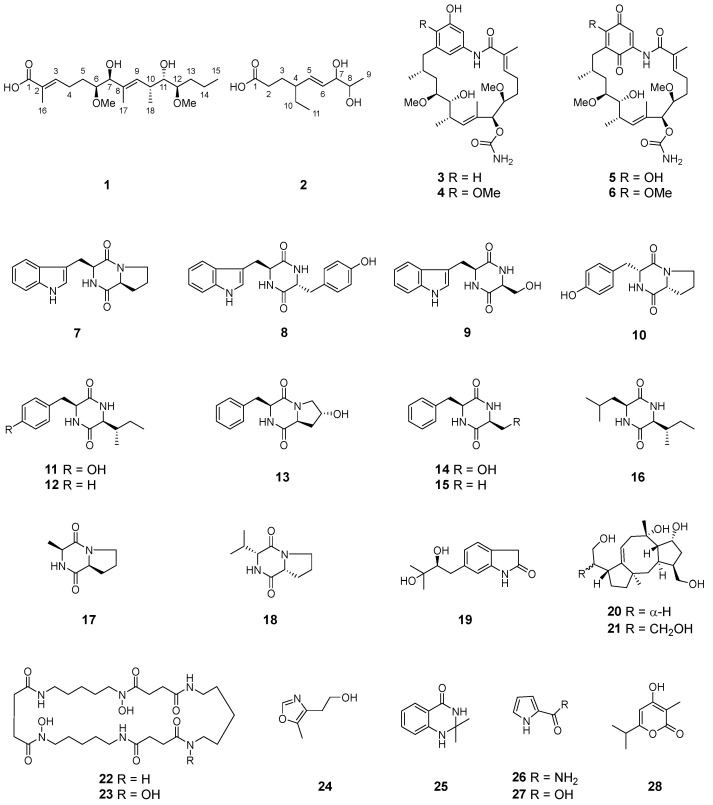
Chemical structures of **1**–**28**.

The ethyl acetate extracts of two mangrove-derived actinomycetes *Streptomyces* sp. WHUA03072 and *Streptomyces* sp. WHUA03267 afforded 28 secondary metabolites (Figure 1) characterized using a combination of spectroscopic methods including NMR, MS, and IR. Compounds **1** and **2** were new and their structures were elucidated as described below. The other 26 of the isolated compounds which were known were identified as autolytimycin (**3**) [29], reblastatin (**4**) [29], 17-*O*-demethylgeldanamycin (**5**) [30], geldanamycin (**6**) [30], cyclo(D-Trp-L-Tyr) (**7**) [31], cyclo(L-Pro-L-Trp) (**8**) [32], cyclo(L-Ser-L-Trp) (**9**) [33], cyclo(L-Tyr-L-Pro) (**10**) [34], cyclo(L-Ile-L-Tyr) (**11**) [35], cyclo(L-Ile-L-Phe) (**12**) [36], cyclo(4-OH-L-Pro-L-Phe) (**13**) [37], cyclo(L-Ser-L-Phe) (**14**) [38], cyclo(L-Phe-L-Ala) (**15**) [36], cyclo(L-Leu-L-Ile) (**16**) [39], cyclo(L-Ala-L-Pro) (**17**) [40], cyclo(L-Pro-L-Val) (**18**) [41], 6-(2,3-dihydroxy-3-methyibutyl)indolin-2-one (**19**) [42], 17-hydroxycyclooctatin (**20**) [43], 16,17-dihydroxycyclooctatin (**21**) [44], terragine E (**22**) [45], deferriferrioxamine E (**23**) [46], 4-(2-hydroxyethyl)-5-methyloxazole (**24**) [47], 2,3-dihydro-2,2-dimethyl-4(1*H*)-quinazolinone (**25**) [48], 1*H*-pyrrole-2-carboxamide (**26**) [49], 1*H*-pyrrole-2-carboxylic acid (**27**) [50], and surugapyrone A (**28**) [51] (Figure 1) by comparing their spectroscopic data with those in the related literatures. To the best of our knowledge, compounds **11** and **24** were obtained from actinomycetes for the first time, while **20**–**21** had never been reported to occur in a marine-derived microorganism previously.

### 2.1. Structure Elucidation

**Figure 2:**
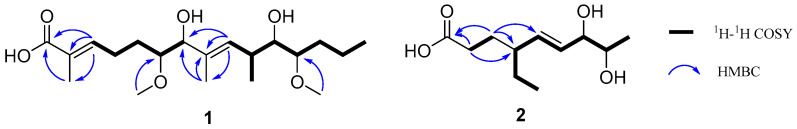
Key ^1^H-^1^H COSY and HMBC correlations in **1** and **2**.

Geldana acid A (**1**) was obtained as colorless oil. The molecular formula of **1** was deduced to be C_20_H_36_O_6_ based on the HR-ESI-MS [M + Na]^+^ ion at *m*/*z* 395.2424 (calculated for C_20_H_36_O_6_Na, 395.2404), comprising three degrees of unsaturation. The IR spectrum of **1** showed absorption bands at 3440 and 1686 cm^−1^, indicating the presence of hydroxyl and carbonyl groups, respectively. The ^1^H- and ^13^C-NMR spectral data for compound **1** were represented in Table 1. The ^13^C-NMR and DEPT spectra revealed the presence of twenty carbons which comprised four methyls, four methylenes, seven methines (four oxygenated at *δ*_C_ 75.5, 81.1, 83.5, and 84.0, and two olefinic at *δ*_C_ 132.5 and 142.9), and three non-protonated carbons (two olefinic at *δ*_C_ 129.6 and 135.4 and one carbonyl at *δ*_C_ 171.3). The ^1^H-NMR spectrum (Supplementary Materials) displayed signals for one methyl triplet, one methyl doublet, two methyl singlets, four oxygenated methine protons, two olefinic protons, and two methoxy singlets. The above-mentioned information suggested the existences of two tri-substituted double bonds and one carbonyl in **1**, accounting for all of the three degrees of unsaturation, as implied by the molecular formula. This deduction proposed that **1** was a chain carboxylic acid derivative. The structure of **1** was established by detailed elucidation of the 2D-NMR spectra (Figure 2). In the ^1^H-^1^H COSY plots, correlations of H_3_-15/H_2_-14, H_2_-14/H_2_-13, H_2_-13/H-12 (δ_H_ 3.08), H-12 (δ_H_ 3.08)/H-11 (δ_H_ 3.55), H-11 (δ_H_ 3.55)/H-10, H-10/H-9 (δ_H_ 5.33), and H-10/H_3_-18 (δ_H_ 1.07) revealed the presence of a structural moiety CH_3_(15)-CH_2_(14)-CH_2_(13)-CH(12)-CH(11)-CH(10)-CH_3_(18)/CH(9)= (A). Similarly, another structural fragment =CH(3)-CH_2_(4)-CH_2_(5)-CH(6)-CH(7)- (B) could also be established by the correlations of H-3 (δ_H_ 6.77)/H_2_-4, H_2_-4/H_2_-5, H_2_-5/H-6 (δ_H_ 3.29), and H-6 (δ_H_ 3.29)/H-7 (δ_H_ 3.93). In the HMBC spectrum, correlations from H_3_-17 (δ_H_ 1.66) to C-7 (δ_C_ 81.0) and C-8 (δ_C_ 135.4), as well as from H-9 (δ_H_ 5.33) to C-7 (δ_C_ 81.0), C-8 (δ_C_ 135.4), and C-17 (δ_C_ 12.2), suggested the linkage of C-7, C-9, and C-17 via the non-protonated olefinic carbon C-8. Additional HMBC correlations from H_3_-16 (δ_H_ 1.84) to C-2 (δ_C_ 129.6) and C-1 (δ_C_ 171.3), and from H-3 (δ_H_ 6.77) to C-2 (δ_C_ 129.6), C-16 (δ_C_ 12.6), and C-1 (δ_C_ 171.3), indicated the linkage of C-1, C-3, and C-16 via the other non-protonated olefinic carbon C-2. Thus, **1** was elucidated to possess a chain structure with multiple asymmetrical factors including tri-substituted double bonds and contiguous chiral centers. Due to the difficulty in defining the dominant conformations, it has always been a challenge to determine the stereochemistry of such a flexible structure as that of **1** by conventional spectroscopic methods or chemical correlations.

Interestingly, four ansamycins **3**–**6**, a class of bacteria-originated antibiotics featuring a rigid aromatic core and an aliphatic ansa chain linked to the nonadjacent positions of the core, were also obtained in the present study. After a detailed structural comparison, compound **1** was found to constitute the common ansa chain of **3**–**6**, suggesting their closely related biogenesis. The skeleton of **3**–**6** was biosynthesized via a common machinery, which was initiated with the formation of 3-amino-5-hydroxybenzoic acid (AHBA), followed by the assembly of the ansa chain to AHBA by the modular polyketide synthase (PKS) and intramolecular lactamization catalyzed by the amide synthase [52,53,54]. The post-PKS tailoring of the ansamycin skeleton by oxidation, elimination, methylation, and carbamoylation generated the structure diversity of ansamycins [52,53,54], as in the case of **3**–**6** which possessed different degrees of oxidation on the AHBA core. Although several natural ansamycins with open-chain structures were identified from bacteria, compound **1** represented the first natural product structurally related to the ansa chain moiety of the ansamycins. It was proposed to be biosynthesized via the same pathway with that of **3**–**6** by employing methylmalonyl-CoA as the starter unit. In view of the co-occurrence of **1**, **3**, and **6** in the culture broth of *Streptomyces* sp. WHUA03072, as well as the structural and biogenetic correlations among them, the stereochemistry of **1** was proposed to be the same with those of **3** and **6**. Compound **1** was thus identified to possess the structure as shown in Figure 1.

Geldana acid B (**2**), colorless oil, was assigned a molecular formula of C_11_H_20_O_4_ based on the HR-ESI-MS [M + Na]^+^ ion at *m*/*z* 239.1249 (calculated for C_11_H_20_O_4_Na, 239.1254), corresponding to two degrees of unsaturation. The IR spectrum of **2** showed absorption bands for hydroxy (3397 cm^−1^) and carbonyl (1708 cm^−1^) groups. The ^13^C-NMR and DEPT spectra revealed the presence of eleven carbon resonances ascribable to two methyls, three methylenes, five methines (two oxygenated at *δ*_C_ 71.8 and 78.3, and two olefinic at *δ*_C_ 131.8 and 137.8), and one carbonyl at *δ*_C_ 178.5. In the ^1^H-NMR spectrum of **2**, signals ascribable to one methyl triplet, one methyl doublet, two oxygenated methine protons, and two olefinic protons could be well distinguished. The above-mentioned information suggested the existence of one di-substituted double bond and one carbonyl in **2**, accounting for both the two degrees of unsaturation implied by the molecular formula. This deduction proposed that **2** was a chain carboxylic acid derivative. The structure of **2** was established by the detailed elucidation of the 2D-NMR spectra (Figure 2). In the ^1^H-^1^H COSY plots, the correlations of H_3_-9 (δ_H_ 1.12)/H-8 (δ_H_ 3.60), H-8 (δ_H_ 3.60)/H-7 (δ_H_ 3.81), H-7 (δ_H_ 3.81)/H-6 (δ_H_ 5.48), H-6 (δ_H_ 5.48)/H-5 (δ_H_ 5.46), H-5 (δ_H_ 5.46)/H-4, H-4/H_2_-10, H_2_-10/H_3_-11 (δ_H_ 0.90), and H_2_-2/H_2_-3 indicated the presence of two structural fragments in **2**, namely CH_3_(9)-CH(8)-CH(7)-CH(6)-CH(5)-CH(4)-CH_2_(10)-CH_3_(11) and CH_2_(2)-CH_2_(3). The connection of CH_2_(3) and CH(4) was supported by the HMBC correlations from H_2_-3 to C-4 and C-5, as well as from H_2_-2 to C-4, in spite of the absence of ^1^H-^1^H COSY correlation between H_2_-3 and H-4. Additional HMBC correlations from H_2_-2 and H_2_-3 to the carbonyl anchored a carboxyl group at C-2. The chemical shifts of C-7 and C-8 in combination with the molecular formula suggested that both C-7 and C-8 were substituted by hydroxy groups for the α,β-unsaturated diol moiety in **2**. A relatively large coupling constant (15.5 Hz) between H-5 and H-6 revealed the *E* configuration of the double bond. In order to determine the relative configuration of the vicinal diol, we tried to prepare the acetonide derivative of **2** by chemical derivatization. Unfortunately, it turned out to be futile by furnishing several products which were difficult to be separated, probably due to the instability of **2** under the reaction condition. Hence, the configuration of C-4, C-7, and C-8 in **2** remained undefined. Compound **2** was proposed to be biosynthesized via a PKS pathway incorporating a rare extender unit ethylmalonyl CoA.

### 2.2. AGS Inhibitory Activity

Compounds **1**–**28** were evaluated for in vitro AGS inhibitory activities employing acarbose as a reference standard for the assay. As a result, compounds **3**, **8**, **9**, **11**, **14**, **16**, and **17** showed a potent to moderate activity with IC_50_ values ranging from 35.76 ± 0.40 to 164.5 ± 15.5 µM (Table 2) compared to acarbose (IC_50_ = 422.3 ± 8.44 μM). It is worth noting that autolytimycin (**3**) exhibited the most potent AGS inhibitory activity. Compounds **3**–**6** are geldanamycin derivatives belonging to the benzoquinone ansamycins antibiotics. These compounds have been reported to effectively inhibit the function of heat shock protein 90 (Hsp90), a molecular chaperone playing a key role in fostering metabolic pathways essential in tumorigenesis, by competing for the ATP binding site on Hsp90 [55]. Compound **3** which exhibited AGS inhibitory activity has a less oxidized AHBA core compared to compounds **4**–**6**, which probably accounted for the observed activity. The ansamycin antibiotic **3** inhibits the function of both Hsp90 and AGS and may probably serve as a multifunctional agent against cancer and diabetes.

Compounds **8**, **9**, **11**, **14**, **16**, and **17** are cyclodipeptides featuring a 2,5-diketopiperazine (2,5-DKP) moiety that has been frequently found in the secondary metabolites of both fungi and bacteria [56]. The conformationally constrained 2,5-DKP moiety has been regarded as a pharmacophore in medicinal chemistry, which endowed natural products embracing 2,5-DKP moiety with a variety of biological properties including antitumor, antiviral, antifungal, antibacterial, neuroprotective, and nootropic activities [57]. In the present study, the AGS inhibitory activity of **9**, **14**, **16**, and **17** was evaluated. It appeared that the substituents played a more influential role in the maintenance of AGS inhibitory activity rather than the common 2,5-DKP moiety itself since the active compounds **8**, **9**, **11**, **14**, **16**, and **17** shared a common 2,5-DKP moiety with the non-active cyclodipeptides obtained in the present study, while bearing different substituents including methyl, hydroxymethyl, isopropyl, isobutyl, phenethyl, and indole groups.

### 2.3. Analysis of Inhibition Kinetics

**Figure 3:**
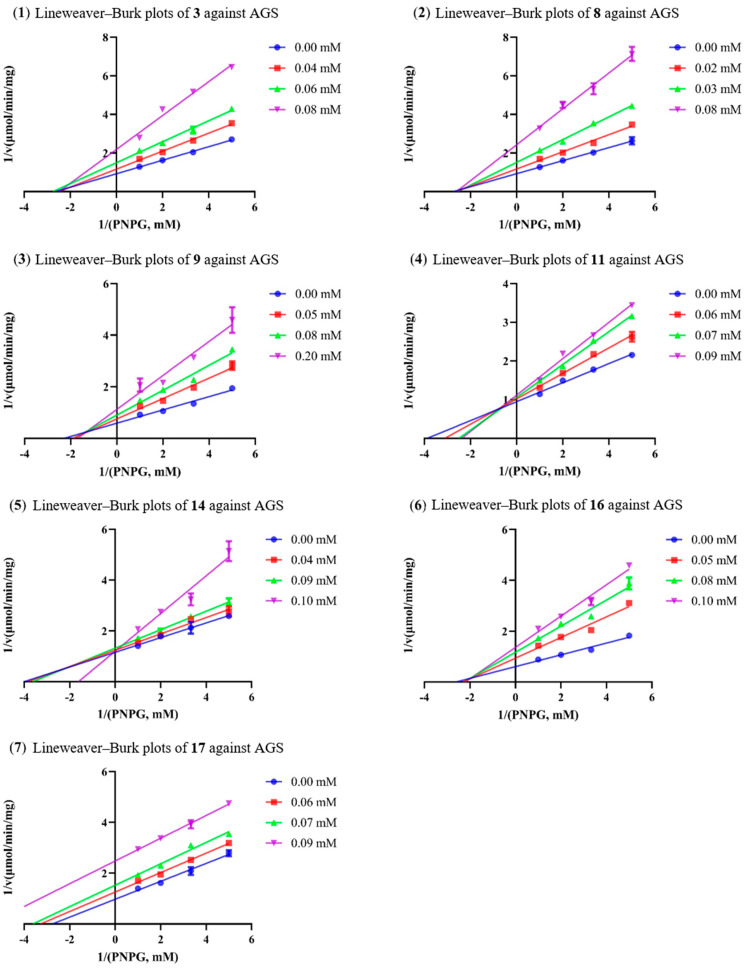
The Lineweaver–Burk plots of **3**, **8**, **9**, **11**, **14**, **16**, and **17** against AGS.

Although several 2,5-DKP containing cyclodipeptides have been reported to inhibit the activity of AGS to different extents, little is known on the inhibition kinetics of these inhibitors. Herein, the inhibition kinetic mechanisms of **3**, **8**, **9**, **11**, **14**, **16**, and **17** against AGS were analyzed using the Lineweaver–Burk plots. The inhibition constants K_i_ and K_i’_ of the inhibitors were calculated by secondary plots of “slope *versus* [I]” and “Y-intercept *versus* [I]”, respectively. As shown in Table 3, both **3** and **8** decreased the maximum velocity (V_max_) while the Michaelis constant (K_m_) was kept at a fixed value. These results suggested that they functioned as non-competitive inhibitors against AGS, which was verified by the intersection of data lines at the x-axis in the Lineweaver–Burk plots (Figure 3). The approximately equal K_i_ and K_i’_ values implied that **3** and **8** could bind to AGS and AGS-substrate complex unbiasedly. All of the data lines of **9**, **11**, and **16** intersected in the second quadrant, suggesting that they inhibited the activity of AGS in a mixed-type manner. In addition, the increased K_m_ values and reduced V_max_ values revealed that the inhibition of AGS by **9**, **11**, and **16** comprised competitive and non-competitive inhibition. As listed in Table 3, all of them possessed a smaller K_i_ value as compared with the corresponding K_i’_ value, indicating that they bound more easily and tightly to the free AGS than the AGS-substrate complex. The smallest K_i_ and K_i’_ of **16** (59.75 and 78.89 µM, respectively) indicated the best inhibitory potency against AGS as compared with those of **9** and **11**, which was consistent with the IC_50_ values. Compound **14** was determined to be a competitive inhibitor of AGS, in view of the intersection of data lines on the y-axis, as well as the increased K_m_ and the constant V_max_ values. Compound **17** was proposed to behave in a non-competitive and uncompetitive mixed-type of inhibition instead of a solely uncompetitive inhibition, since the data lines would eventually intersect in the third quadrant. This postulation was reinforced by the varied K_m_/V_max_ values which were not in accordance with uncompetitive inhibition. However, the replots of the slope and y-intercept versus the concentration of **14** and **17** were not linearly fitted, which limited the application of Equations (3) and (4). Consequently, the K_i_ or K_i’_ of **14** and **17** remained not calculated. It was interesting that seven cyclodipeptide congeners tended to inhibit the activity of AGS in four different manners, which was proposed to be caused by different substitutions on the 2,5-DKP moiety.

### 2.4. Molecular Docking Studies

**Figure 4:**
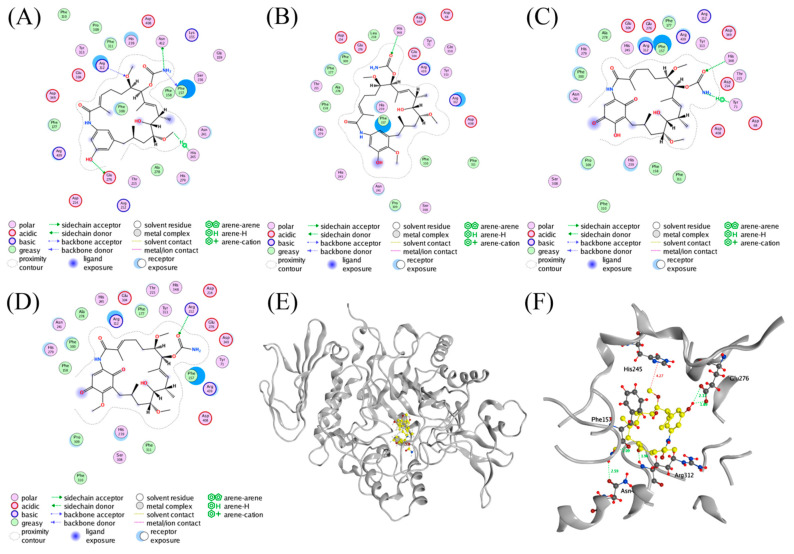
Ligand interactions of (**A**) **3**, (**B**) **4**, (**C**) **5**, and (**D**) **6** with AGS. (**E**) Stereo diagram of **3** in the active site of AGS. (**F**) Binding modes of **3** with AGS.

A molecular docking was performed to investigate the interactions between AGS and the inhibitors using MOE software (Version 2014. 09, Chemical Computing Group Inc., Montreal, QC, Canada). Compound **3** was predicted to form hydrogen bond interactions with Glu276 (2.39 and 1.89 Å), Phe157 (2.09 Å), Asn412 (2.59 Å), and Arg312 (1.96 Å) of AGS via the phenolic hydroxyl (18-OH), amino, and methoxy (6-OMe) groups (Figure 4A,F). In addition, the presence of the other methoxy group (12-OMe) in **3** enhanced the stability of the complex by forming a hydrogen-π interaction with His245 (4.27 Å). On the contrary, another three geldanamycin derivatives **4**–**6** that exhibited weak AGS inhibitory activity in the preliminary screening were found to form less protein-ligand interactions (Figure 4B–D) as compared with those of **3**, probably due to the conformational alterations induced by the oxidation of the benzene ring in the structure. The results of the docking study may partially explain the discrepancy of **3**–**6** in inhibiting the activity of AGS.

**Figure 5:**
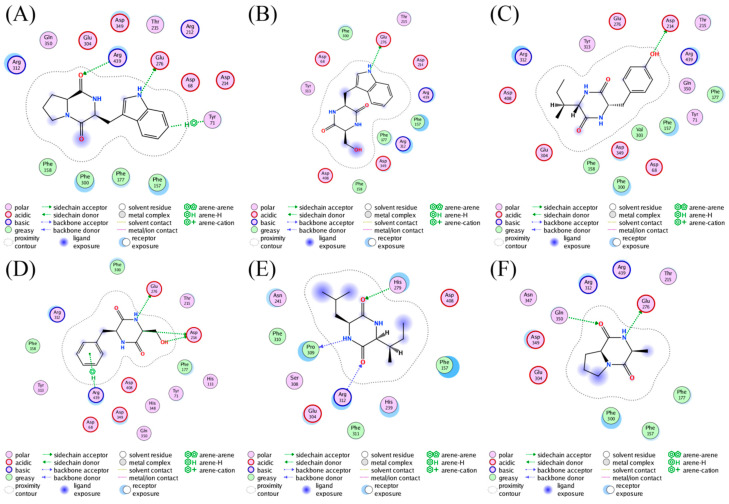
Ligand interactions of (**A**) **8**, (**B**) **9**, (**C**) **11**, (**D**) **14**, (**E**) **16**, and (**F**) **17** with AGS.

The bioactive cyclodipeptides **8**, **9**, **11**, **14**, **16**, and **17** could be well docked into the active site of AGS (Figure 5). Specifically, cyclo(L-Pro-L-Trp) (**8**)**,** a derivative with the most potent activity, formed hydrogen bond interactions with Arg439 (2.49 Å) and Glu276 (1.98 Å) via the carbonyl of the 2,5-DKP moiety and the nitrogen atom of the indole moiety, respectively. Furthermore, the hydrogen-π interaction between the phenyl ring of the indole moiety in **8** and Tyr71 (4.16 Å) also contributed to the stabilization of the complex. As shown in Figure 4, similar interactions could also be found between AGS and **14** that exhibited comparable activity with **8**. These interactions were proposed to play pivotal roles in retaining the AGS inhibitory activity of **8** and **14**. However, it seemed that the potent activity of either **16** or **17** was attributed to the interactions between the 2,5-DKP moiety and AGS solely. The less active compounds **9** and **11** against AGS were predicted to form fewer interactions with protein residues in the active site of this enzyme compared to **8**, **14**, **16**, and **17**. Acarbose was reported to form interactions with Asp326, Arg197, and Asn258 of AGS [58].

### 2.5. In Vitro Cytotoxicity Assay

To preliminarily evaluate the safety of the AGS inhibitors, the in vitro cytotoxicity of **3**, **8**, **9**, **11**, **14**, **16**, and **17** toward human normal hepatocyte (LO2) cells was tested by MTT assay. As a result, the IC_50_ values of them were all >100 μM (Table 4), suggesting that those promising AGS inhibitors were nontoxic. Since cancer and diabetes are linked, these compounds need to be evaluated for cytotoxicity against a panel of cancer cell lines to assess their safety profile at least in vitro in future studies.

## 3. Materials and Methods

### 3.1. General Experimental Procedure

Optical rotations were measured on a Rudolph Research Autopol III polarimeter (Rudolph Research Analytical, Hackettstown, NJ, USA). IR spectra were obtained on a Thermo Nicolet 6700 FT-IR microscope instrument (Thermo Electron Corporation, Waltham, MA, USA). The UV spectra were obtained on a TU-1900 ultraviolet spectrometer (Beijing Persee General Instrument Co., Ltd., Beijing, China). HR-ESI-MS was performed on an Agilent-6210-LC/TOF mass spectrometer (Agilent Technologies, Inc., Santa Clara, CA, USA). The NMR spectra were obtained on a Bruker Avance 600 spectrometer (Bruker Corporation, Billerica, MA, USA). Silica gel (300–400 mesh; Qingdao Marine Chemical Co., Ltd., Qingdao, China), MCI CHP 20P gel (75–150 μm, Tokyo, Japan), ODS AQ C-18 gel (50 μm; YMC Co., Ltd., Kyoto, Japan), and Sephadex LH-20 gel (GE Healthcare, Uppsala, Sweden) were used for CC. Precoated GF254 silica gel plates (Qingdao Marine Chemical Co., Ltd., Qingdao, China) were used for thin layer chromatography. Semi-preparative HPLC was carried out on an Agilent ZORBAX Eclipse XDB-C18 column (5 μm, 250 × 9.4 mm) with a Shimadzu LC-20AT system by eluting with CH_3_OH/H_2_O solvent system at 3 mL/min. A SpectraMax Plus 384 microplate reader (Molecular Devices, San Jose, CA, USA) was used in the AGS inhibition assay. 

### 3.2. Microorganisms

*Streptomyces* sp. WHUA03072 and *Streptomyces* sp. WHUA03267 were isolated from the mangrove sediment collected at Bailu Park and Hongsha Bay of Sanya, Hainan Province, respectively. Both of the two strains were identified as *Streptomyces* spp. based on the 16S rDNA sequencing. Voucher samples were preserved at the Key Laboratory of Combinatorial Biosynthesis and Drug Discovery, Ministry of Education, School of Pharmaceutical Sciences, Wuhan University, Wuhan, China.

### 3.3. Fermentation, Extraction, and Isolation

*Streptomyces* sp. WHUA03072 was cultured in 2 L polypropylene flasks containing 1 L liquid ISP_2_ medium (malt extract 10 g/L, yeast extract 4 g/L, glucose 4 g/L, pH 7.2 ± 0.2) on a rotatory shaker at 180 rpm and 30 °C for 7 days. At the end of fermentation, all culture broth (50 L) was combined and filtered through a cheesecloth. The filtrate was concentrated under a vacuum to ca. 3 L and partitioned with ethyl acetate (EtOAc) 3 × 3 L. The organic extract solution was collected and evaporated under a vacuum to provide an EtOAc-soluble residue A (10.64 g). *Streptomyces* sp. WHUA03267 was fermented and processed in the same way to afford another EtOAc-soluble residue B (7.65 g).

Residue A (10.64 g) was separated by column chromatography (CC) on MCI CHP 20P gel (CH_3_OH-H_2_O (10:90 → 100:0, *v*/*v*)) to give five fractions (Fr. A–E). Fr. B (1.0 g) was chromatographed by ODS AQ C-18 CC (CH_3_OH-H_2_O (10:90 → 60:40, *v*/*v*)) to provide six subfractions (Fr. B1–B6). Fr. B1 was purified by semi-preparative HPLC (CH_3_OH-H_2_O (20:80, *v*/*v*)) to offer **9** (3.0 mg, t_R_ = 18.0 min). Fr. B3 was purified by silica gel CC (CH_2_Cl_2_-CH_3_OH (12:1, *v*/*v*)) to yield two subfractions (Fr. B3A–B3B). Fr. B3A was purified by semi-preparative HPLC (CH_3_OH-H_2_O (28:72, *v*/*v*)) to furnish **13** (1.2 mg, t_R_ = 18.0 min) and **15** (2.2 mg, t_R_ = 19.0 min). Fr. B5 was subjected to silica gel CC (CH_2_Cl_2_-CH_3_OH (20:1, *v*/*v*)) to offer **19** (8.3 mg) and a subfraction Fr. B5A, which was further purified by semi-preparative HPLC (CH_3_OH-H_2_O (32:68, *v*/*v*)) to furnish **21** (4.7 mg, t_R_ = 24.0 min). Fr. B6 was purified by silica gel CC (CH_2_Cl_2_-CH_3_OH (12:1, *v*/*v*)) to give **2** (3.5 mg). Fr. C (3.0 g) was isolated by ODS AQ C-18 CC (CH_3_OH-H_2_O (20:80 → 100:0, *v*/*v*)) to yield nine subfractions (Fr. C1–C9). Fr. C2 was purified by semi-preparative HPLC (CH_3_OH-H_2_O (27:73, *v*/*v*)) to offer **1** (1.9 mg, t_R_ = 29.0 min). Fr. C3 was isolated by Sephadex LH-20 CC (CH_3_OH), followed by semi-preparative HPLC (CH_3_OH-H_2_O (40:60, *v*/*v*)) to afford **22** (8.4 mg, t_R_ = 15.2 min). Fr. C4 was chromatographed over silica gel (CH_2_Cl_2_-CH_3_OH (100:1 → 12:1, *v*/*v*)), followed by semi-preparative HPLC (CH_3_OH-H_2_O (37:63, *v*/*v*)) to give **28** (1.1 mg, t_R_ = 25.2 min). Fr. C7 was separated by silica gel CC (CH_2_Cl_2_-CH_3_OH (25:1, *v*/*v*)) to afford **16** (3.4 mg). Fr. C8 was subjected to silica gel CC (CH_2_Cl_2_-CH_3_OH (50:1 → 20:1, *v*/*v*)) to give **3** (5.0 mg). Fr. C9 was loaded onto Sephadex LH-20 CC (CH_3_OH), followed by silica gel CC (CH_2_Cl_2_-CH_3_OH (20:1, *v*/*v*)) to give **20** (8.0 mg). Fr. E (3.60 g) was fractionated by CC on silica gel (petroleum ether-EtOAc (10:1 → 1:10, *v*/*v*)) to yield nine subfractions (Fr. E1–E9). Fr. E9 was purified by semi-preparative-HPLC (CH_3_OH-H_2_O (65:35, *v*/*v*)) and was further purified on a silica gel column (CH_2_Cl_2_-CH_3_OH (50:1, *v*/*v*)) to give **6** (11.2 mg).

Residue B (7.65 g) was first separated by CC on MCI CHP 20P gel (CH_3_OH-H_2_O (20:80 → 100:0, *v*/*v*)) to afford eight fractions (Fr. A–H). Fr. C (0.65 g) was chromatographed on ODS AQ C-18 gel (CH_3_OH-H_2_O (5:95 → 100:0, *v*/*v*)) to provide six subfractions (Fr. C1–C6). Fr. C4 was purified on a silica gel column (CH_2_Cl_2_-CH_3_OH (1:0 → 4:1, *v*/*v*)) to give **26** (7.8 mg) and a subfraction Fr. C4A, which was further purified by semi-preparative HPLC (CH_3_OH-H_2_O (11:89, *v*/*v*)) to furnish **17** (10.5 mg, t_R_ = 10.7 min). Fr. D (0.60 g) was separated by CC on Sephadex LH-20 (CH_3_OH) to give six subfractions (Fr. D1–D6). Fr. D3 was chromatographed over silica gel (CH_2_Cl_2_-CH_3_OH (1:0 → 4:1, *v*/*v*)), followed by semi-preparative HPLC (CH_3_OH-H_2_O (20:80, *v*/*v*)) to afford **24** (6.5 mg, t_R_ = 11.5 min). Fr. D4 was successively fractioned by ODS AQ C-18 CC (CH_3_OH-H_2_O (10:90 → 100:0, *v*/*v*)) and Sephadex LH-20 CC (CH_3_OH) to give five subfractions (Fr. D4B1–D4B5). Fr. D4B3 was separated by semi-preparative HPLC (CH_3_OH-H_2_O (10:90, *v*/*v*)) to give six subfractions (Fr. D4B3A–D4B3F). In addition, **14** (12.0 mg, t_R_ = 14.0 min) and **10** (4.4 mg, t_R_ = 14.2 min) were obtained from Fr. D4B3B and D4B3F, respectively, by semi-preparative HPLC (CH_3_OH-H_2_O (20:80, *v*/*v*)). Fr. D5 was subjected to ODS AQ C-18 CC (CH_3_OH-H_2_O (5:95 → 100:0, *v*/*v*)), followed by semi-preparative HPLC (CH_3_OH-H_2_O (13:87, *v*/*v*)) to furnish **27** (8.9 mg, t_R_ = 21.5 min). Fr. E (1.02 g) was fractionated using ODS AQ C-18 CC (CH_3_OH-H_2_O (40:60 → 100:0, *v*/*v*)) to yield seven subfractions (Fr. E1–E7). Fr. E2 was purified by Sephadex LH-20 CC (CH_3_OH), followed by semi-preparative HPLC (CH_3_OH-H_2_O (18:82, *v*/*v*)) to afford **18** (5.4 mg, t_R_ = 22.1 min). Fr. E3 was fractionated by Sephadex LH-20 CC (CH_3_OH) to give seven subfractions (Fr. E3A–E3G). Fr. E3A was fractioned by silica gel CC (CH_2_Cl_2_-CH_3_OH (70:1 → 20:1, *v*/*v*)) to afford four subfractions (Fr. E3A1–E3A4). Fr. E3A2 was purified by semi-preparative HPLC (CH_3_OH-H_2_O (23:77, *v*/*v*)) to give **25** (7.1 mg, t_R_ = 28.7 min). Fr. E3A4 was purified by semi-preparative HPLC (CH_3_OH-H_2_O (28:72, *v*/*v*)) to give **11** (1.7 mg, t_R_ = 28.3 min). Fr. E3D was purified by semi-preparative HPLC (CH_3_OH-H_2_O (28:72, *v*/*v*)) to yield **7** (2.6 mg, t_R_ = 30.0 min). Fr. F (0.70 g) was fractionated by ODS AQ C-18 CC (CH_3_OH-H_2_O (50:50 → 100:0, *v*/*v*)) to give twelve fractions (Fr. F1–F12). Fr. F1 was chromatographed over Sephadex LH-20 CC (CH_3_OH) to offer **23** (3.6 mg) and a subfraction Fr. F1A that was further purified by preparative TLC (CH_2_Cl_2_-CH_3_OH (12:1, *v*/*v*)) to afford **8** (3.1 mg). Fr. F6 was initially fractioned by Sephadex LH-20 CC (CH_3_OH) to give six sub-fractions (Fr. F6A–F6F). Fr. F6B was purified by semi-preparative HPLC (CH_3_OH-H_2_O (43:57, *v*/*v*)) to afford **4** (7.5 mg, t_R_ = 47.5 min). Fr. F6C was purified by semi-preparative HPLC (CH_3_OH-H_2_O (41:59, *v*/*v*)) to furnish **12** (2.4 mg, t_R_ = 49.7 min). The purification of Fr. G (0.47 g) by silica gel CC (CH_2_Cl_2_-CH_3_OH (60:1 → 0:1, *v*/*v*)) yielded a sub-fraction G1, which was further separated by Sephadex LH-20 CC (CH_3_OH) and semi-preparative HPLC (CH_3_OH-H_2_O (50:50, *v*/*v*)) to give **5** (1.3 mg, t_R_ = 47.3 min).

Geldana acid A (**1**), colorless oil, ${\left[\alpha \right]}_{D}^{20}$: −20 (*c* 0.1, MeOH), IR (KBr): 3440, 2925, 1686, and 1084 cm^−1^. UV λ_max_ (MeOH) nm (log*ε*): 215 (4.14); HR-ESI-MS *m*/*z*: 395.2424 [M + Na]^+^ (calculated for C_20_H_36_O_6_Na, 395.2404). For ^1^H- and ^13^C-NMR data, see Table 1.

Geldana acid B (**2**), colorless oil, ${\left[\alpha \right]}_{D}^{20}$: −10 (*c* 0.1, MeOH), IR (KBr): 3397, 2921, 2581, 1708, and 1646 cm^−1^. HR-ESI-MS *m*/*z*: 239.1249 [M + Na]^+^ (calculated for C_11_H_20_O_4_Na, 239.1254). For ^1^H- and ^13^C-NMR data, see Table 1.

### 3.4. AGS Inhibition Assay

The AGS inhibitory activity was evaluated following the methods reported in the literature [23,24,25].

### 3.5. Inhibition Kinetics Analysis

The mode of inhibition was determined by the Lineweaver–Burk plot using GraphPad Prism 8.0 software. The K_m_ and V_max_ values were obtained from the slope and y-axis intercept of the Lineweaver–Burk plot based on Equations (1) and (2):

(1) $$V_{\max}=\frac{1}{Y-\mathrm{intercept}}$$

(2) $$K_{m}=\mathrm{slope}\times {\;V}_{\max}$$

The K_i_ and K_i’_ values were calculated by secondary plotting of the slope and y-intercept on the Lineweaver–Burk plot versus the inhibitor [I] based on Equations (3) and (4):

(3) $$\mathrm{Slope}=\frac{K_{m}}{V_{\max}}+\frac{K_{m}\left[I\right]}{V_{\max}K_{i}}$$

(4) $$Y-\mathrm{intercept}=\frac{1}{V_{\max}}+\frac{\left[I\right]}{V_{\max}K_{i^{\prime }}}$$

### 3.6. Molecular Docking

Molecular docking between the inhibitors and AGS was performed following the method we reported previously [28].

### 3.7. Cell Culture and Cytotoxicity Assay

The LO2 cell line was cultured in a 1640 medium supplemented with 10% fetal bovine serum, 100 U/mL of penicillin, and 0.1 mg/mL of streptomycin in a humidified incubator with a 5% CO_2_ atmosphere at 37 °C. The cells were seeded in 96-well plates at a density of 3 × 10^3^ cells per well. After 12 h, the medium in the wells was replaced by 100 µL of fresh medium containing drugs of six different concentrations. After incubation for another 48 h, the medium was removed and the fresh medium containing 10 µL of MTT (5 mg/mL) were added to each well and incubated for 4 h. Then, the medium was replaced by 100 µL of DMSO to dissolve the formazan crystals. OD_570_ was detected on a microplate reader. Parallel triplicate replication was performed for each dose, and the IC_50_ values were calculated.

## 4. Conclusions

A total of 28 compounds, including two previously undescribed polyketides **1**–**2**, four benzenoid ansamycins **3**–**6**, fourteen diketopiperazine derivatives **7**–**18**, one prenylated indole derivative **19**, two fusicoccane-type diterpenoids **20**–**21**, two hydroxamate siderophore **22**–**23**, and five others **24**–**28**, were isolated from the ethyl acetate extracts of two mangrove associated actinomycetes, *Streptomyces* sp. WHUA03267 and *Streptomyces* sp. WHUA03072. These compounds were structurally characterized by a combination of spectroscopic methods including NMR, MS, and IR. Among all of the isolates, **11** and **24** were obtained from actinomycetes for the first time. In the in vitro AGS inhibitory assay, autolytimycin (**3**) significantly inhibited the activity of AGS in a non-competitive manner. To the best of our knowledge, it was the first report on the AGS inhibitory activity of an ansamycin derivative, which enriched the functional diversity of ansamycin antibiotics. Cyclodipeptides, i.e., **8**, **9**, **11**, **14**, **16**, and **17**, were also found to be responsible for the AGS inhibitory activity of the two strains, among which, **9**, **14**, **16**, and **17** had never been reported as AGS inhibitors previously. As revealed by kinetic analysis, these bioactive cyclopeptides functioned in multiple manners despite the presence of the common 2,5-DKP moiety in the structures, emphasizing the importance of both the 2,5-DKP moiety and the substituents in retaining the AGS inhibitory activity. It was also supported by the results of molecular docking studies.

## Figures and Tables

**Table 1:** ^1^H- (600 MHz) and ^13^C-NMR (150 MHz) spectroscopic data for **1** and **2** in CD_3_OD.

Position	1		2	
	*δ*_C_, Type	*δ*_H_ (Multi, *J* in Hz)	*δ*_C_, Type	*δ*_H_ (Multi, *J* in Hz)
1	171.3, C	-	178.5, C	
2	129.6, C	-	33.4, CH_2_	2.25 (m)
3	142.9, CH	6.77 (t, 7.2)	31.2, CH_2_	1.76 (m) 1.50 (m)
4	25.4, CH_2_	2.32 (m)	45.4, CH	1.94 (m)
5	30.9, CH_2_	1.53 (m) 1.45 (m)	137.8, CH	5.46 (dd, 15.5, 8.0)
6	83.5, CH	3.29 (td, 7.8, 1.8)	131.8, CH	5.48 (dd, 15.5, 6.3)
7	81.0, CH	3.93 (d, 7.2)	78.3, CH	3.81 (dd, 6.3, 6.3)
8	135.4, C	-	71.8, CH	3.60 (m)
9	132.5, CH	5.33 (d, 10.2)	19.0, CH_3_	1.12 (d, 6.3)
10	36.0, CH	2.52 (m)	29.1, CH_2_	1.50 (m) 1.30 (m)
11	75.5, CH	3.55 (dd, 9.0, 3.6)	12.1, CH_3_	0.90 (t, 7.2)
12	84.0, CH	3.08 (dt, 9.0, 3.0)	-	-
13	31.6, CH_2_	1.52 (m) 1.36 (m)	-	-
14	20.3, CH_2_	1.55 (m) 1.28 (m)	-	-
15	14.8, CH_3_	0.93 (t, 7.2)	-	-
16	12.6, CH_3_	1.84 (s)	-	-
17	12.2, CH_3_	1.66 (s)	-	-
18	17.5, CH_3_	1.07 (d, 6.6)	-	-
6-OMe	59.1, CH_3_	3.49 (s)	-	-
12-OMe	57.3, CH_3_	3.34 (s)	-	-

**Table 2:** AGS inhibitory activity of **1**–**28**.

Compounds	IC_50_ (μM) ^a^	Compounds	IC_50_ (μM) ^a^
**1**	NA ^b^	**15**	NA ^b^
**2**	NA ^b^	**16**	73.98 ± 1.25
**3**	35.76 ± 0.40	**17**	56.75 ± 0.32
**4**	NA ^b^	**18**	NA ^b^
**5**	NA ^b^	**19**	NA ^b^
**6**	NA ^b^	**20**	NA ^b^
**7**	NA ^b^	**21**	NA ^b^
**8**	53.85 ± 1.88	**22**	NA ^b^
**9**	164.5 ± 15.5	**23**	NA ^b^
**10**	NA ^b^	**24**	NA ^b^
**11**	101.1 ± 2.52	**25**	NA ^b^
**12**	NA ^b^	**26**	NA ^b^
**13**	NA ^b^	**27**	NA ^b^
**14**	62.20 ± 0.11	**28**	NA ^b^
**Acarbose**	422.3 ± 8.44		

^a^ Data are presented as means ± SD; ^b^ NA: not active.

**Table 3:** Inhibition kinetics of **3**, **8**, **9**, **11**, **14**, **16**, and **17** against AGS.

Compounds	Concentration (mM)	K_m_ (mM)	V_max_ (μM/min)	K_i_ (μM)	K_i’_ (μM)
**3**	0.00	0.38 ± 0.02	1.08 ± 0.03	49.18	53.10
	0.04	0.40 ± 0.03	0.86 ± 0.03		
	0.06	0.36 ± 0.04	0.67 ± 0.04		
	0.08	0.40 ± 0.05	0.46 ± 0.03		
**8**	0.00	0.36 ± 0.04	1.08 ± 0.07	43.89	46.28
	0.02	0.38 ± 0.04	0.85 ± 0.05		
	0.03	0.39 ± 0.03	0.66 ± 0.03		
	0.08	0.38 ± 0.05	0.41 ± 0.03		
**9**	0.00	0.44 ± 0.07	1.70 ± 0.16	148.94	238.78
	0.05	0.53 ± 0.11	1.33 ± 0.17		
	0.08	0.53 ± 0.11	1.11 ± 0.14		
	0.20	0.59 ± 0.22	0.89 ± 0.22		
**11**	0.00	0.26 ± 0.02	1.05 ± 0.04	95.52	531.53
	0.06	0.32 ± 0.03	0.98 ± 0.05		
	0.07	0.40 ± 0.02	0.94 ± 0.02		
	0.09	0.42 ± 0.04	0.90 ± 0.05		
**14**	0.00	0.25 ± 0.04	0.86 ± 0.06	NC ^a^	-
	0.04	0.26 ± 0.04	0.81 ± 0.06		
	0.09	0.28 ± 0.03	0.76 ± 0.04		
	0.10	0.62 ± 0.21	0.83 ± 0.18		
**16**	0.00	0.38 ± 0.06	1.66 ± 0.15	59.75	78.89
	0.05	0.43 ± 0.09	1.07 ± 0.13		
	0.08	0.44 ± 0.10	0.86 ± 0.11		
	0.10	0.45 ± 0.07	0.74 ± 0.07		
**17**	0.00	0.36 ± 0.05	1.03 ± 0.08	NC ^a^	NC ^a^
	0.06	0.30 ± 0.02	0.79 ± 0.02		
	0.07	0.28 ± 0.03	0.66 ± 0.03		
	0.09	0.25 ± 0.01	0.47 ± 0.01		

^a^ NC: not calculated.

**Table 4:** Cytotoxicity of **3**, **8**, **9**, **11**, **14**, **16**, and **17** against LO2 cells.

Compounds	IC_50_ (μM)
**3**	>100
**8**	>100
**9**	>100
**11**	>100
**14**	>100
**16**	>100
**17**	>100

## Data Availability

Data are contained within the article and Supplementary Materials.

## Supplementary Materials

The following are available online at https://www.mdpi.com/article/10.3390/molecules28093822/s1, Figure S1a: ^1^H-NMR spectrum of **1**; Figure S1b: ^13^C-NMR spectrum of **1**; Figure S1c: HSQC spectrum of **1**; Figure S1d: HMBC spectrum of **1**; Figure S1e: DEPT spectrum of **1**; Figure S1f: ^1^H-^1^H COSY spectrum of **1**; Figure S1g: NOESY spectrum of **1**; Figure S1h: The IR (KBr disc) spectrum of **1**; Figure S1i: HR-ESI-MS spectroscopic data for **1**; Figure S1j: UV spectrum of **1**; Figure S2a: ^1^H-NMR spectrum of **2**; Figure S2b: ^13^C-NMR spectrum of **2**; Figure S2c: HSQC spectrum of **2**; Figure S2d: HMBC spectrum of **2**; Figure S2e: DEPT spectrum of **2**; Figure S2f: ^1^H-^1^H COSY spectrum of **2**; Figure S2g: The IR (KBr disc) spectrum of **2**; Figure S2h: HR-ESI-MS spectroscopic data for **2**; Figure S3a: ^1^H-NMR spectrum of antolytimycin (**3**); Figure S3b: ^13^C-NMR spectrum of antolytimycin (**3**); Figure S4a: ^1^H-NMR spectrum of reblastatin (**4**); Figure S4b: ^13^C-NMR spectrum of reblastatin (**4**); Figure S5a: ^1^H-NMR spectrum of 17-*O*-demethylgeldanamycin (**5**); Figure S5b: ^13^C-NMR spectrum of 17-*O*-demethylgeldanamycin (**5**); Figure S6a: ^1^H-NMR spectrum of geldanamycin (**6**); Figure S6b: ^13^C-NMR spectrum of geldanamycin (**6**); Figure S7a: ^1^H-NMR spectrum of cyclo(D-Trp-L-Tyr) (**7**); Figure S7b: ^13^C-NMR spectrum of cyclo(D-Trp-L-Tyr) (**7**); Figure S8a: ^1^H-NMR spectrum of cyclo(L-Pro-L-Trp) (**8**); Figure S8b: ^13^C-NMR spectrum of cyclo(L-Pro-L-Trp) (**8**); Figure S9a: ^1^H-NMR spectrum of cyclo(L-Ser-L-Trp) (**9**); Figure S9b: ^13^C-NMR spectrum of cyclo(L-Ser-L-Trp) (**9**); Figure S10a: ^1^H-NMR spectrum of cyclo(L-Tyr-L-Pro) (**10**); Figure S10b: ^13^C-NMR spectrum of cyclo(L-Tyr-L-Pro) (**10**); Figure S11a: ^1^H-NMR spectrum of cyclo(L-Ile-L-Tyr) (**11**); Figure S11b: ^13^C-NMR spectrum of cyclo(L-Ile-L-Tyr) (**11**); Figure S12a: ^1^H-NMR spectrum of cyclo(L-Ile-L-Phe) (**12**); Figure S12b: ^13^C-NMR spectrum of cyclo(L-Ile-L-Phe) (**12**); Figure S13a: ^1^H-NMR spectrum of cyclo(4-OH-L-Pro-L-Phe) (**13**); Figure S13b: ^13^C-NMR spectrum of cyclo(4-OH-L-Pro-L-Phe) (**13**); Figure S14a: ^1^H-NMR spectrum of cyclo(L-Ser-L-Phe) (**14**); Figure S14b: ^13^C-NMR spectrum of cyclo(L-Ser-L-Phe) (**14**); Figure S15a: ^1^H-NMR spectrum of cyclo(L-Phe-L-Ala) (**15**); Figure S15b: ^13^C-NMR spectrum of cyclo(L-Phe-L-Ala) (**15**); Figure S16a: ^1^H-NMR spectrum of cyclo(L-Leu-L-Ile) (**16**); Figure S16b: ^13^C-NMR spectrum of cyclo(L-Leu-L-Ile) (**16**); Figure S17a: ^1^H-NMR spectrum of cyclo(L-Ala-L-Pro) (**17**); Figure S17b: ^13^C-NMR spectrum of cyclo(L-Ala-L-Pro) (**17**); Figure S18a: ^1^H-NMR spectrum of cyclo(L-Pro-L-Val) (**18**); Figure S18b: ^13^C-NMR spectrum of cyclo(L-Pro-L-Val) (**18**); Figure S19a: ^1^H-NMR spectrum of 6-(2,3-dihydroxy-3-methyibutyl)indolin-2-one (**19**); Figure S19b: ^13^C-NMR spectrum of 6-(2,3-dihydroxy-3-methyibutyl)indolin-2-one (**19**); Figure S20a: ^1^H-NMR spectrum of 17-hydroxycyclooctatin (**20**); Figure S20b: ^13^C-NMR spectrum of 17-hydroxycyclooctatin (**20**); Figure S21a: ^1^H-NMR spectrum of 16,17-dihydroxycyclooctatin (**21**); Figure S21b: ^13^C-NMR spectrum of 16,17-dihydroxycyclooctatin (**21**); Figure S22a: ^1^H-NMR spectrum of terragine E (**22**); Figure S22b: ^13^C-NMR spectrum of terragine E (**22**); Figure S23a: ^1^H-NMR spectrum of deferriferrioxamine E (**23**); Figure S23b: ^13^C-NMR spectrum of deferriferrioxamine E (**23**); Figure S24a: ^1^H-NMR spectrum of 4-(2-hydroxyethyl)-5-methyloxazole (**24**); Figure S24b: ^13^C-NMR spectrum of 4-(2-hydroxyethyl)-5-methyloxazole (**24**); Figure S25a: ^1^H-NMR spectrum of 2,3-dihydro-2,2-dimethyl-4 (1*H*)-quinazolinone (**25**); Figure S25b: ^13^C-NMR spectrum of 2,3-dihydro-2,2-dimethyl-4 (1*H*)-quinazolinone (**25**); Figure S26a: ^1^H-NMR spectrum of 1*H*-pyrrole-2-carboxamide (**26**); Figure S26b: ^13^C-NMR spectrum of 1*H*-pyrrole-2-carboxamide (**26**); Figure S27a: ^1^H-NMR spectrum of 1*H*-pyrrole-2-carboxylic acid (**27**); Figure S27b: ^13^C-NMR spectrum of 1*H*-pyrrole-2-carboxylic acid (**27**); Figure S28a: ^1^H-NMR spectrum of surugapyrone A (**28**); Figure S28b: ^13^C-NMR spectrum of surugapyrone A (**28**); Figure S29: HPLC profiles of residues A and B.

## Sample Availability

Samples of the compounds **1**–**28** are available from the authors.
