# Supplementary material for: α-Glucosidase Inhibitors from Two Mangrove-Derived Actinomycetes
Source: Molecules. 2023 Apr 29;28(9):3822. doi: 10.3390/molecules28093822 (PMC10180428; doi:10.3390/molecules28093822)
Supplement: Supplementary file 1 [file molecules-28-03822-s001.zip › molecules-2359336-supplementary.pdf]

molecules

Supplementary Material

α-Glucosidase Inhibitors from Two Mangrove-Derived Actinomycetes

Xuejun Lu [1,†], Manlai Zhang [1,†], Yixian Qiu [1,†], Xiuxiu Liu [1], Cancan Wang [1], Jianwei Chen [1], Huawei Zhang [1], Bin Wei [1], Yanlei Yu [1], Youmin Ying [1,*], Kui Hong [2,*] and Hong Wang [1,3,*]

1 College of Pharmaceutical Science & Collaborative Innovation Center of Yangtze River Delta Region Green Pharmaceuticals, Zhejiang University of Technology, Hangzhou 310014, China
2 Key Laboratory of Combinatorial Biosynthesis and Drug Discovery, Ministry of Education, School of Pharmaceutical Sciences, Wuhan University, Wuhan, 430072, China
3 Key Laboratory of Marine Fishery Resources Exploitment and Utilization of Zhejiang Province, Zhejiang University of Technology, Hangzhou 310014, China
* Correspondence: ymying@zjut.edu.cn (Y.Y.); kuihong31@whu.edu.cn (K.H.); hongw@zjut.edu.cn (H.W.)
† These authors contributed equally to this work.

CONTENTS

Figure S1a. ^{1}H NMR spectrum of **1** in CD_3OD. 4
Figure S1b. ^{13}C NMR spectrum of **1** in CD_3OD. 4
Figure S1c. HSQC spectrum of **1** in CD_3OD. 5
Figure S1d. HMBC spectrum of **1** in CD_3OD. 5
Figure S1e. DEPT spectrum of **1** in CD_3OD. 6
Figure S1f. ^{1}H-^{1}H COSY spectrum of **1** in CD_3OD. 6
Figure S1g. NOESY spectrum of **1** in CD_3OD. 7
Figure S1h. The IR (KBr disc) spectrum of **1**. 7
Figure S1i. The HR-ESI-MS spectroscopic data of **1**. 7
Figure S1j. UV spectrum of **1**. 8
Figure S2a. ^{1}H NMR spectrum of **2** in CD_3OD. 8
Figure S2b. ^{13}C NMR spectrum of **2** in CD_3OD. 9
Figure S2c. HSQC spectrum of **2** in CD_3OD. 9
Figure S2d. HMBC spectrum of **2** inCD_3OD. 9
Figure S2e. DEPT spectrum of **2** in CD_3OD. 10
Figure S2f. ^{1}H-^{1}H COSY spectrum of **2** in CD_3OD. 10
Figure S2g. The IR (KBr disc) spectrum of **2.** 11
Figure S2h. The HR-ESI-MS spectroscopic data of **2** 11
Figure S3a. ^{1}H NMR spectrum of **3** in DMSO. 12
Figure S3b. ^{13}C NMR spectrum of **3** in DMSO. 12
Figure S4a. ^{1}H NMR spectrum of **4** in CD_3OD. 13
Figure S4b. ^{13}C NMR spectrum of **4** in CD_3OD. 13
Figure S5a. ^{1}H NMR spectrum of **5** in $CDCl_3$. 14
Figure S5b. ^{13}C NMR spectrum of **5** in $CDCl_3$. 14
Figure S6a. ^{1}H NMR spectrum of **6** in $CDCl_3$. 15
Figure S6b. ^{13}C NMR spectrum of **6** in $CDCl_3$. 15
Figure S7a. ^{1}H NMR spectrum of **7** in CD_3OD. 16

Figure S7b. ^{13}C NMR spectrum of **7** in CD_3OD. 16
Figure S8a. ^{1}H NMR spectrum of **8** in $CDCl_3$. 17
Figure S8b. ^{13}C NMR spectrum of **8** in $CDCl_3$. 17
Figure S9a. ^{1}H NMR spectrum of **9** in CD_3OD. 18
Figure S9b. ^{13}C NMR spectrum of **9** in CD_3OD. 18
Figure S10a. ^{1}H NMR spectrum of **10** in CD_3OD. 19
Figure S10b. ^{13}C NMR spectrum of **10** in CD_3OD. 19
Figure S11a. ^{1}H NMR spectrum of **11** in CD_3OD. 20
Figure S11b. ^{13}C NMR spectrum of **11** in CD_3OD. 20
Figure S12a. ^{1}H NMR spectrum of **12** in DMSO. 21
Figure S12b. ^{13}C NMR spectrum of **12** in DMSO. 21
Figure S13a. ^{1}H NMR spectrum of **13** in CD_3OD. 22
Figure S13b. ^{13}C NMR spectrum of **13** in CD_3OD. 22
Figure S14a. ^{1}H NMR spectrum of **14** in DMSO. 23
Figure S14b. ^{13}C NMR spectrum of **14** in DMSO. 23
Figure S15a. ^{1}H NMR spectrum of **15** in CD_3OD. 24
Figure S15b. ^{13}C NMR spectrum of **15** in CD_3OD. 24
Figure S16a. ^{1}H NMR spectrum of **16** in $CDCl_3$. 25
Figure S16b. ^{13}C NMR spectrum of **16** in $CDCl_3$. 25
Figure S17a. ^{1}H NMR spectrum of **17** in DMSO. 26
Figure S17b. ^{13}C NMR spectrum of **17** in DMSO. 26
Figure S18a. ^{1}H NMR spectrum of **18** in CD_3OD. 27
Figure S18b. ^{13}C NMR spectrum of **18** in CD_3OD. 27
Figure S19a. ^{1}H NMR spectrum of **19** in DMSO. 28
Figure S19b. ^{13}C NMR spectrum of **19** in DMSO. 28
Figure S20a. ^{1}H NMR spectrum of **20** in CD_3OD. 29
Figure S20b. ^{13}C NMR spectrum of **20** in CD_3OD. 29
Figure S21a. ^{1}H NMR spectrum of **21** in CD_3OD. 30
Figure S21b. ^{13}C NMR spectrum of **21** in CD_3OD. 30
Figure S22a. ^{1}H NMR spectrum of **22** in DMSO. 31
Figure S22b. ^{13}C NMR spectrum of **22** in DMSO. 31
Figure S23a. ^{1}H NMR spectrum of **23** in DMSO. 32
Figure S23b. ^{13}C NMR spectrum of **23** in DMSO. 32
Figure S24a. ^{1}H NMR spectrum of **24** in DMSO. 33
Figure S24b. ^{13}C NMR spectrum of **24** in DMSO. 33
Figure S25a. ^{1}H NMR spectrum of **25** in CD_3OD. 34
Figure S25b. ^{13}C NMR spectrum of **25** in CD_3OD. 34
Figure S26a. ^{1}H NMR spectrum of **26** in DMSO. 35
Figure S26b. ^{13}C NMR spectrum of **26** in DMSO. 35
Figure S27a. ^{1}H NMR spectrum of **27** in CD_3OD. 36
Figure S27b. ^{13}C NMR spectrum of **27** in CD_3OD. 36
Figure S28a. ^{1}H NMR spectrum of **28** in CD_3OD. 37

Figure S28b. ^{13}C NMR spectrum of **28** in CD3OD....................37
Figure S29. HPLC profiles of residues A and B....................38
NMR data for **3–28.**....................38
References....................41

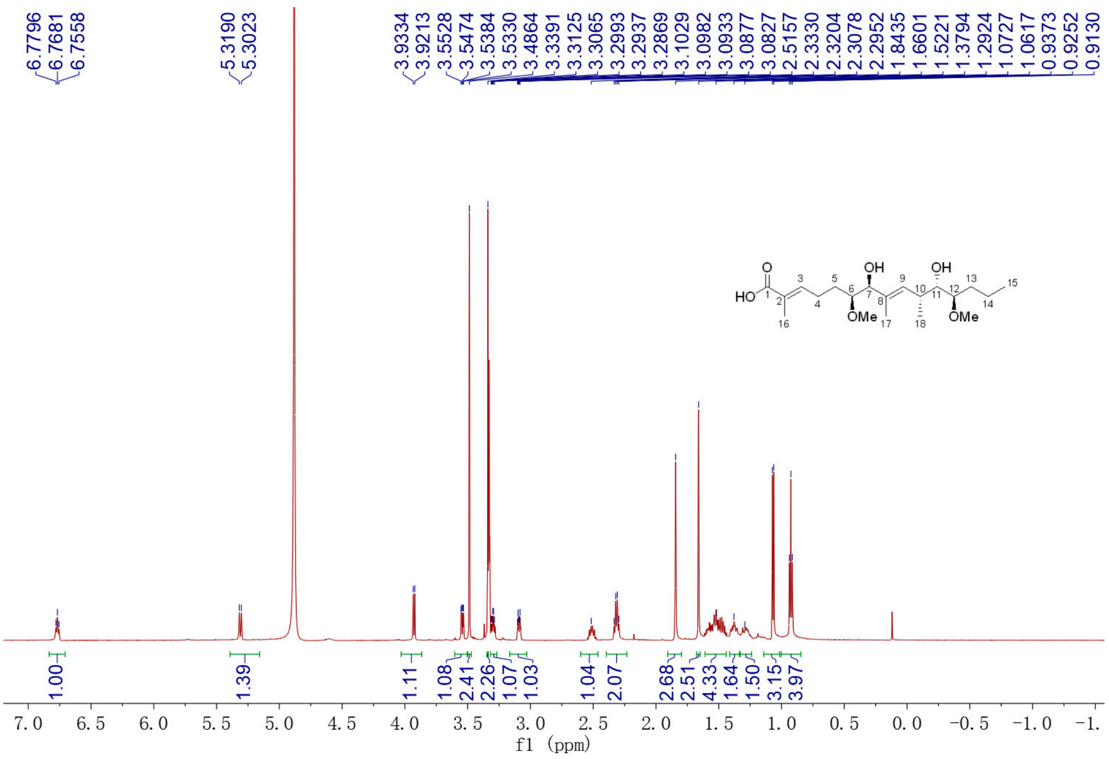

Figure S1a. ^{1}H NMR spectrum of **1** in CD3OD.

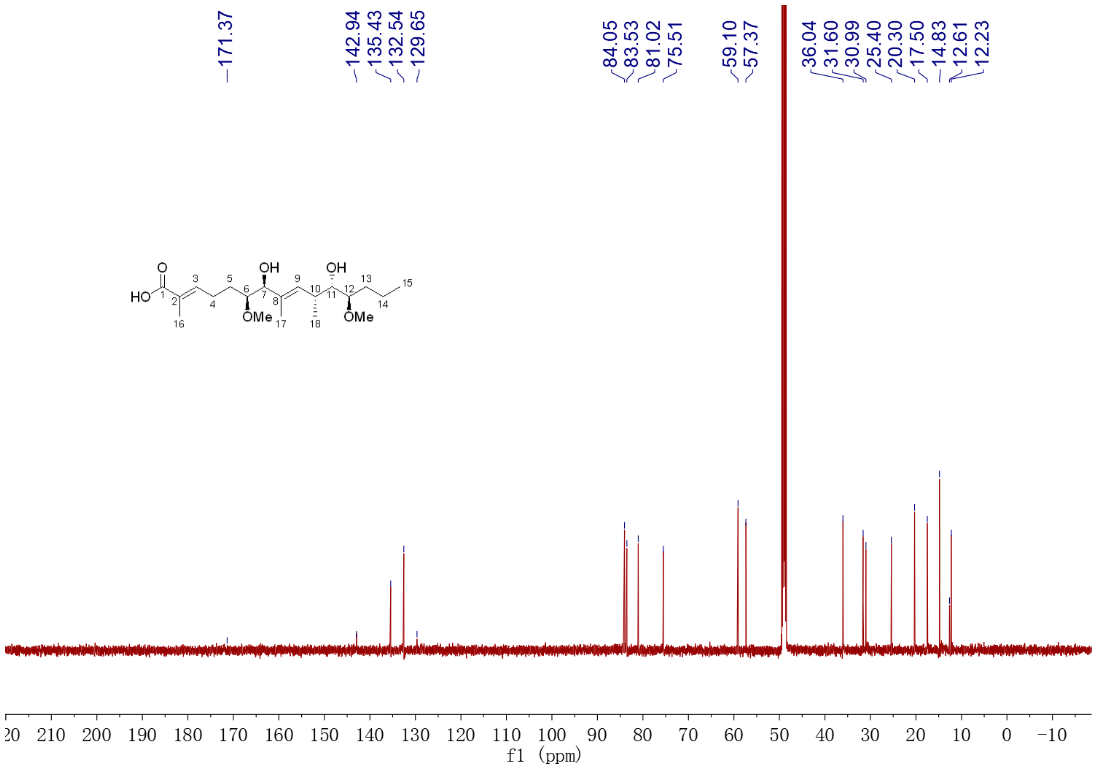

Figure S1b. ^{13}C NMR spectrum of **1** in CD3OD.

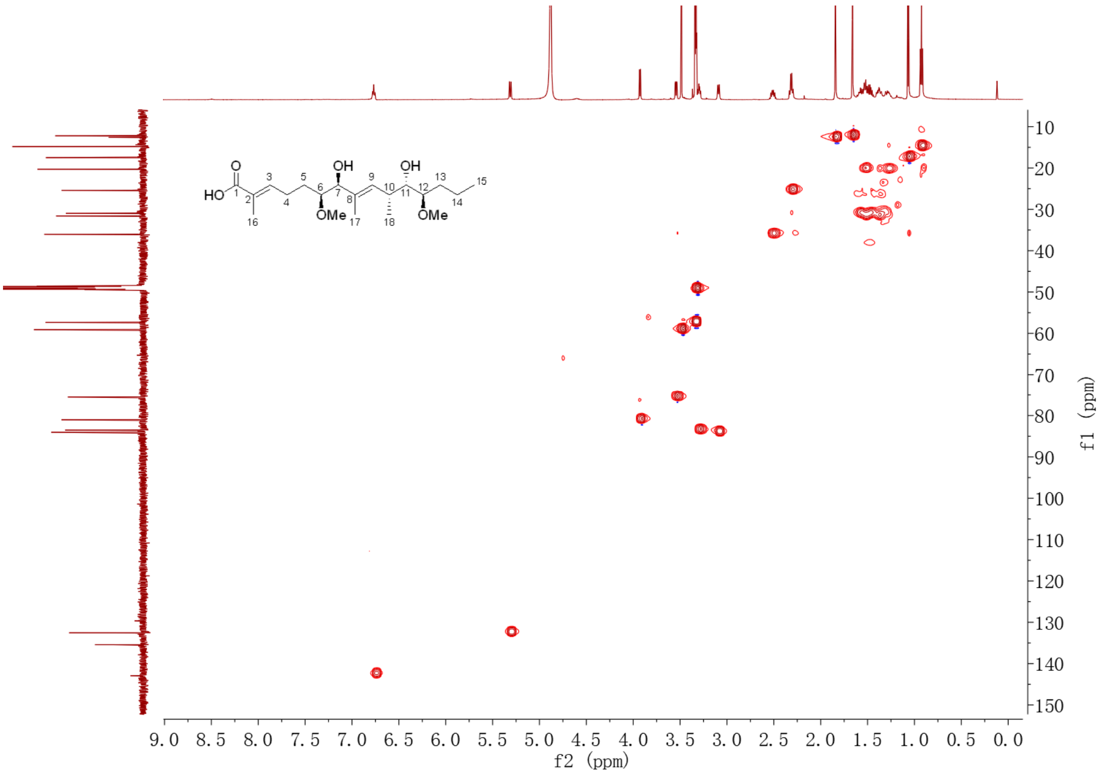

Figure S1c. HSQC spectrum of **1** in CD3OD.

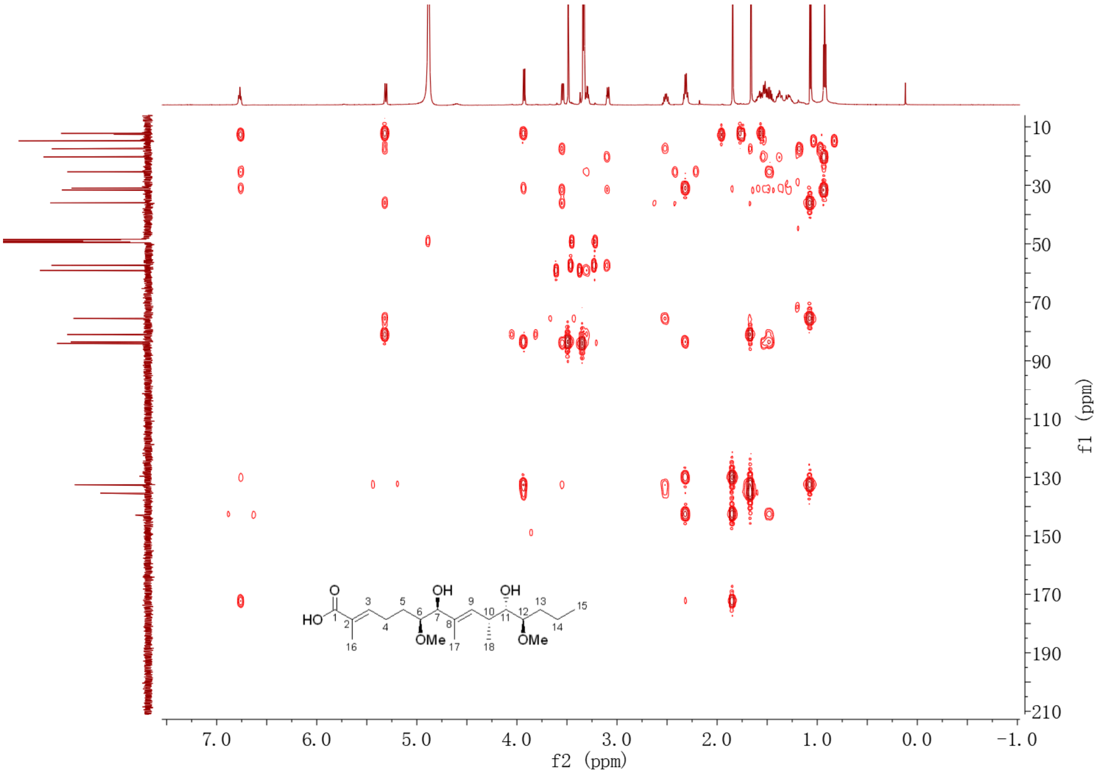

Figure S1d. HMBC spectrum of **1** in CD3OD.

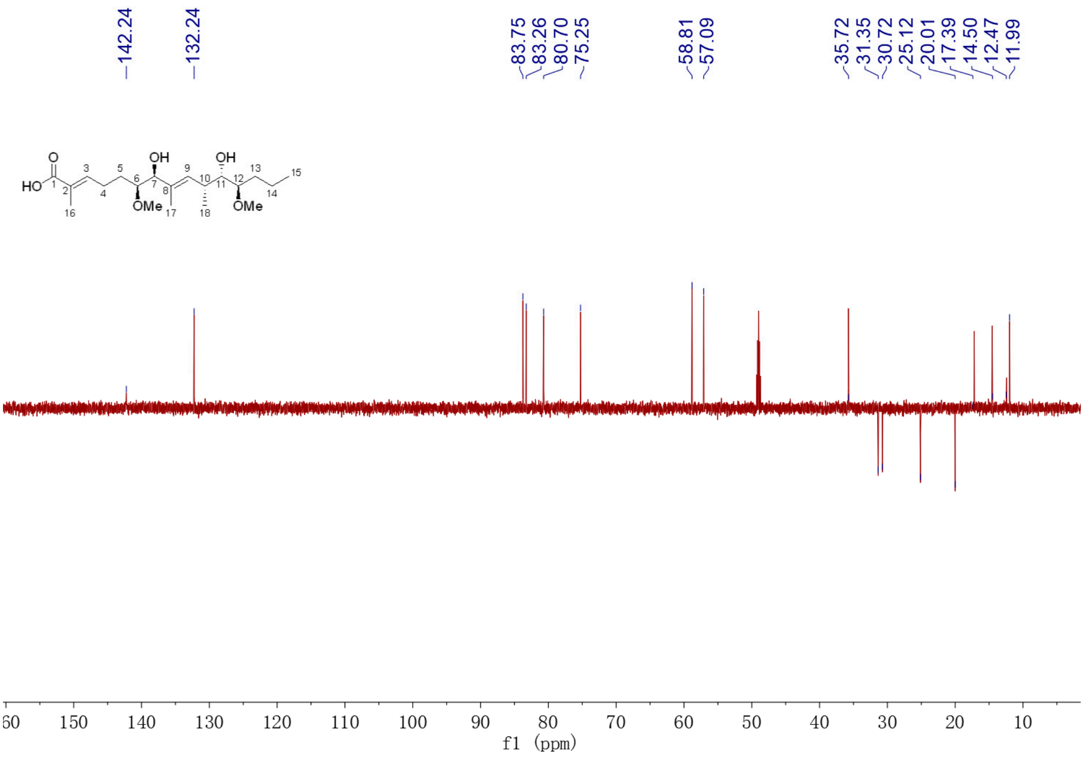

Figure S1e. DEPT spectrum of **1** in CD3OD.

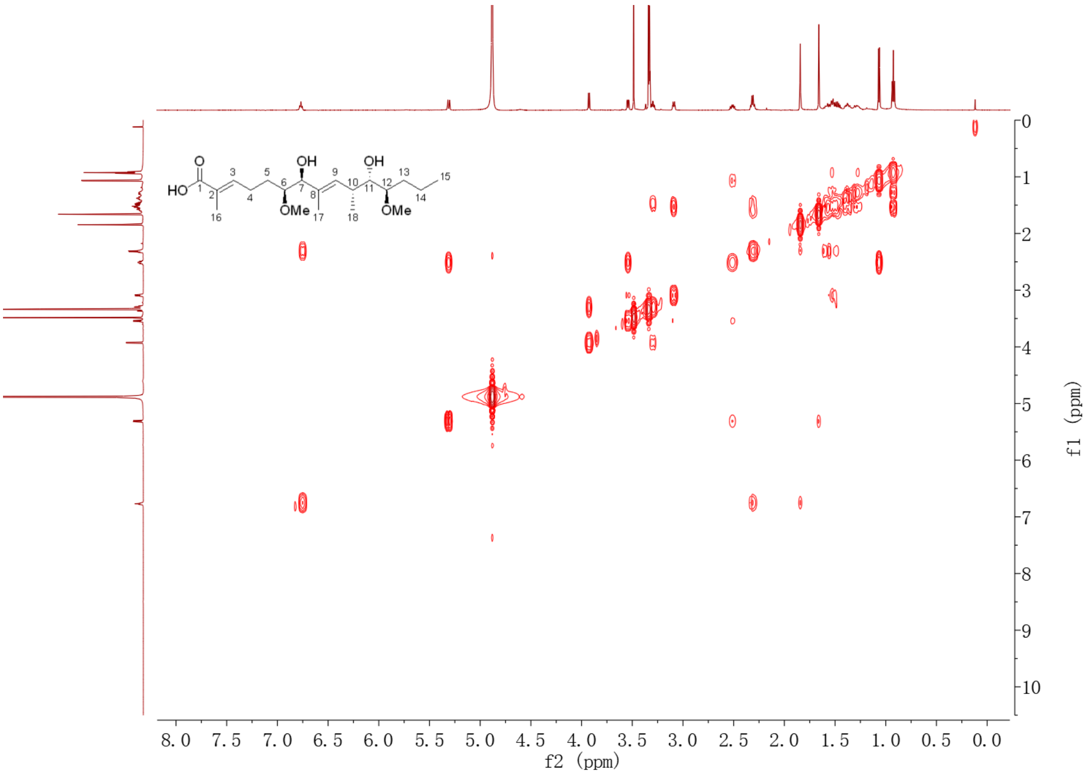

Figure S1f. ^{1}H-^{1}H COSY spectrum of **1** in CD3OD.

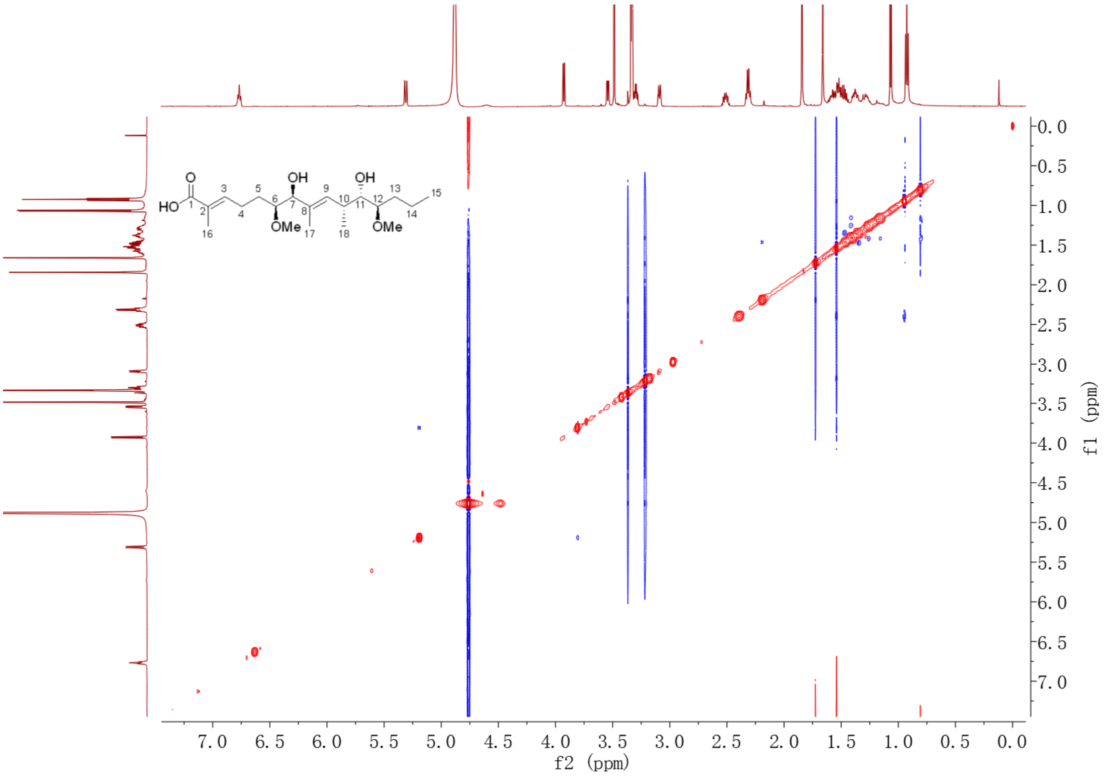

Figure S1g. NOESY spectrum of **1** in CD_3OD.

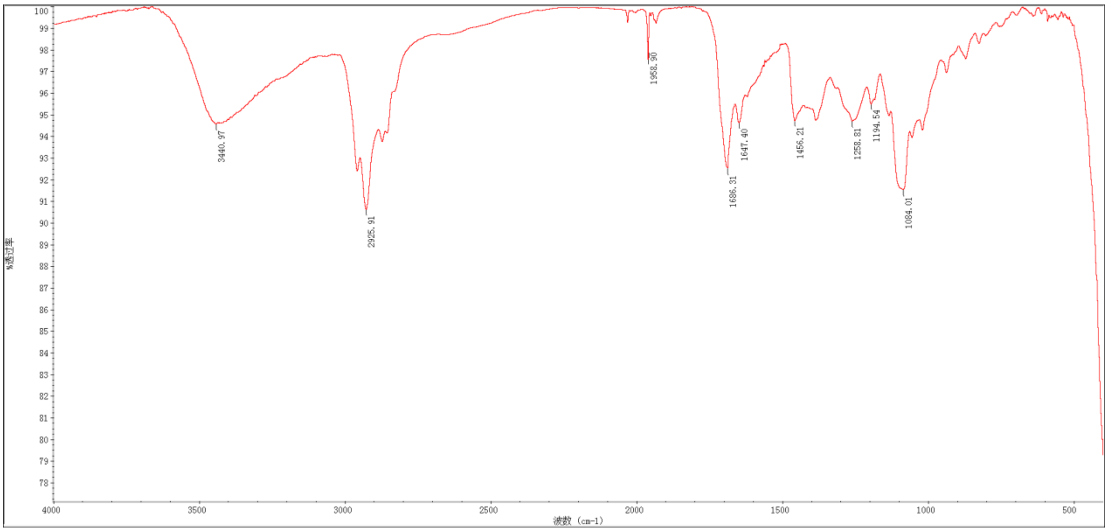

Figure S1h. The IR (KBr disc) spectrum of **1**.

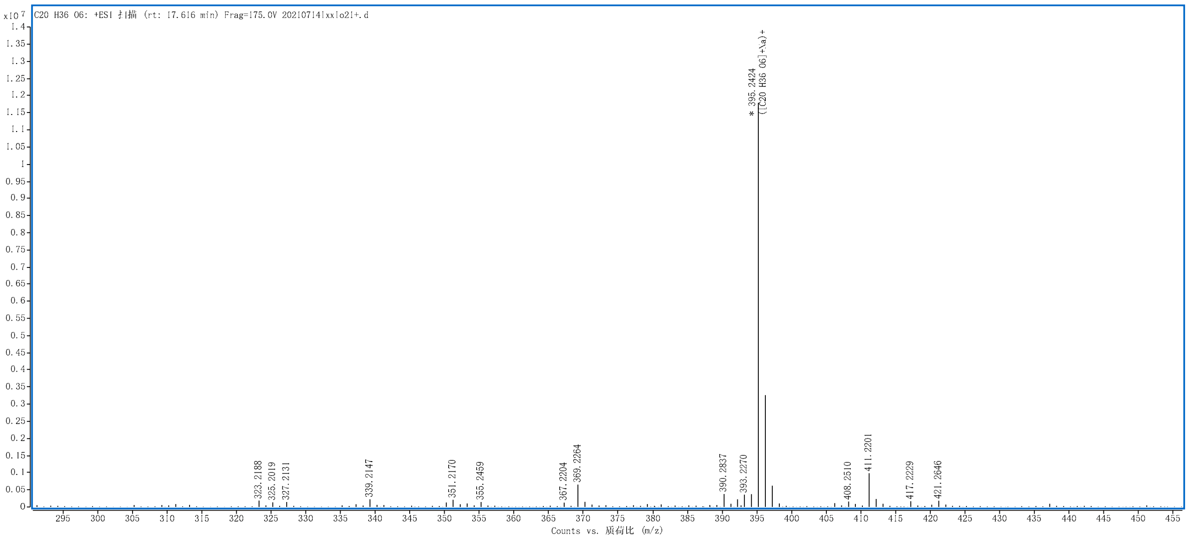

Formula (M)	Ion Formula	m/z	Calc m/z	Diff (ppm)	DBE
$C_{20}H_{36}O_6$	$C_{20}H_{36}NaO_6$	395.2424	395.2404	-4.67	3

Figure S1i. The HR-ESI-MS spectroscopic data of **1**.

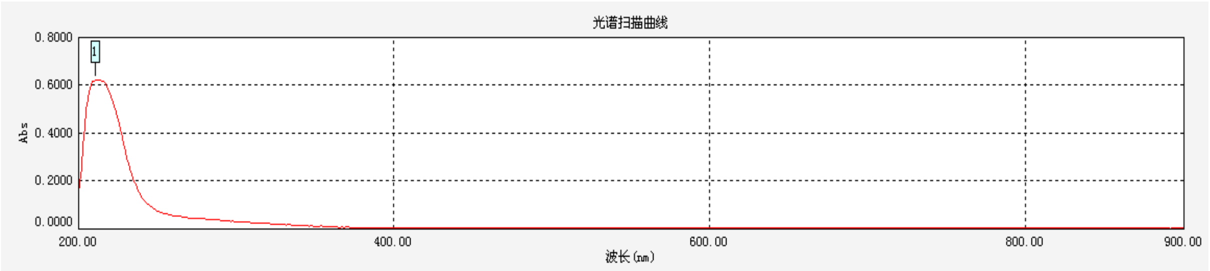

P	λ (nm)	Abs

1	215	0.6198

Figure S1j. UV spectrum of **1**.

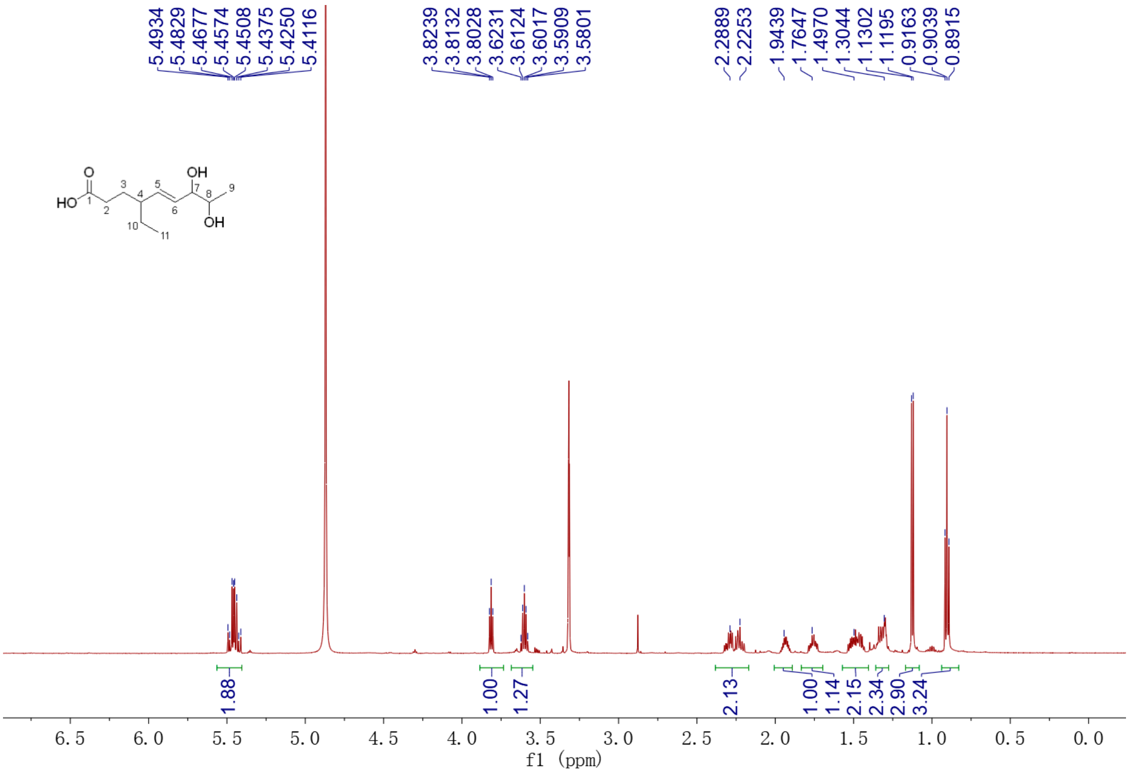

Figure S2a. ^{1}H NMR spectrum of **2** in CD_3OD.

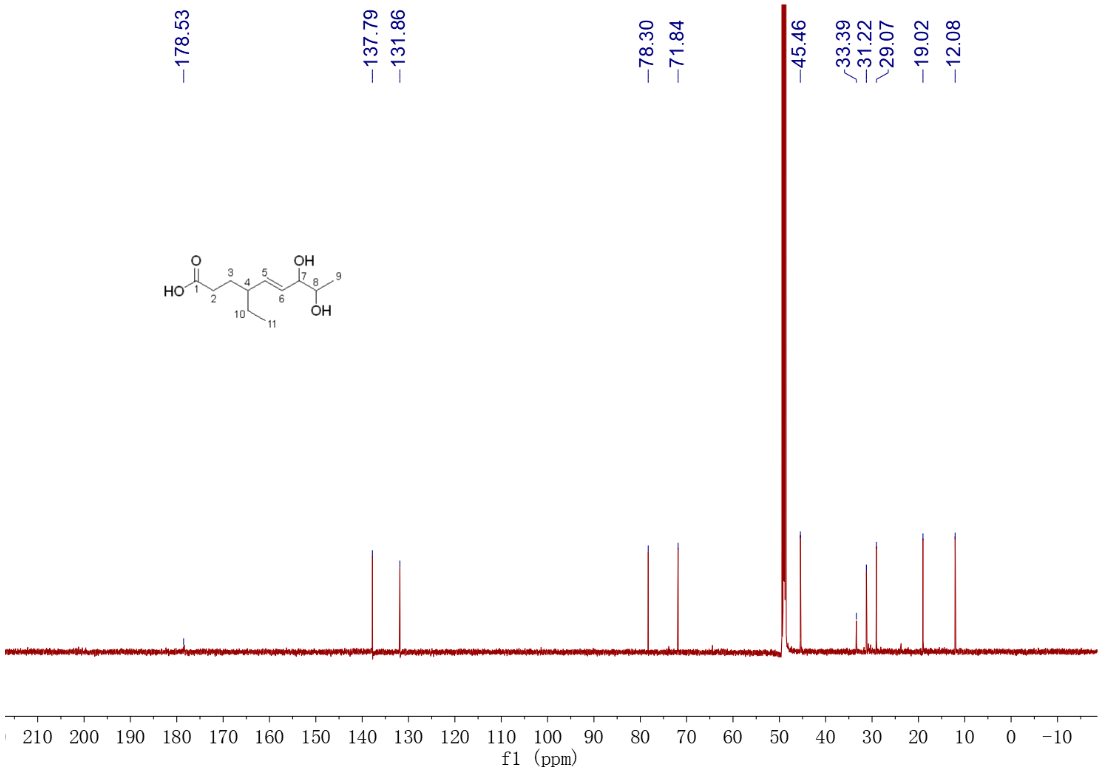

Figure S2b. ^{13}C NMR spectrum of **2** in CD_3OD.

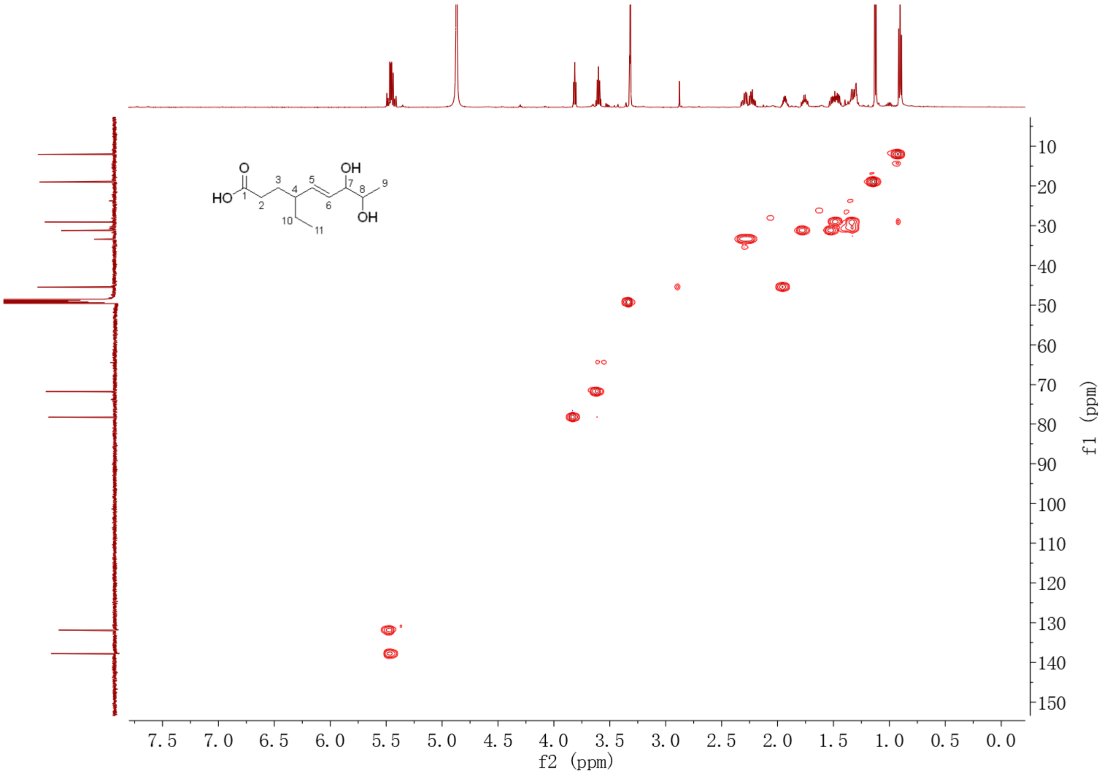

Figure S2c. HSQC spectrum of **2** in CD_3OD.

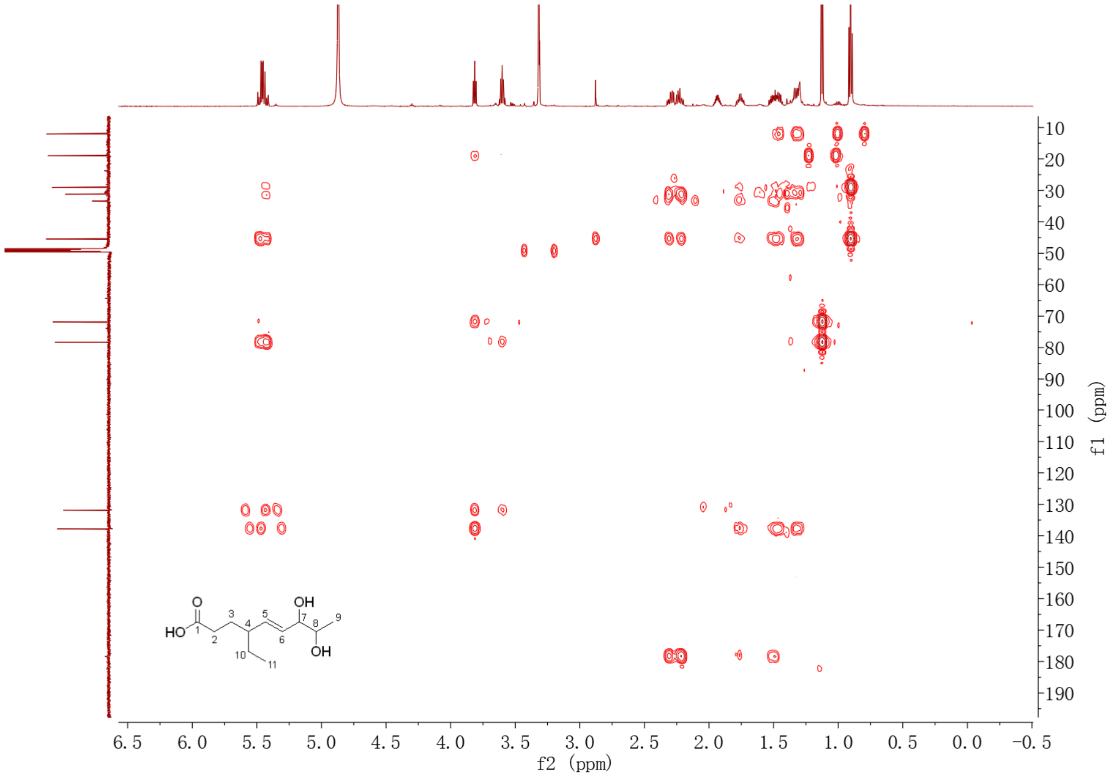

Figure S2d. HMBC spectrum of **2** inCD_3OD.

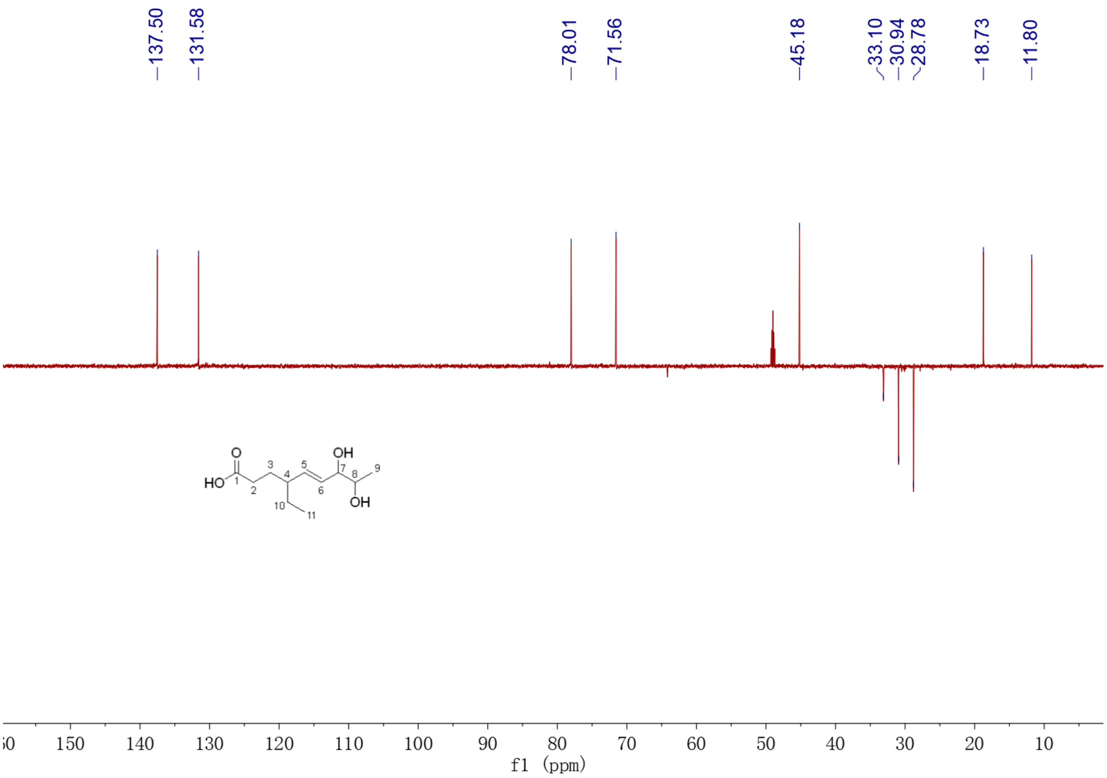

Figure S2e. DEPT spectrum of **2** in CD3OD.

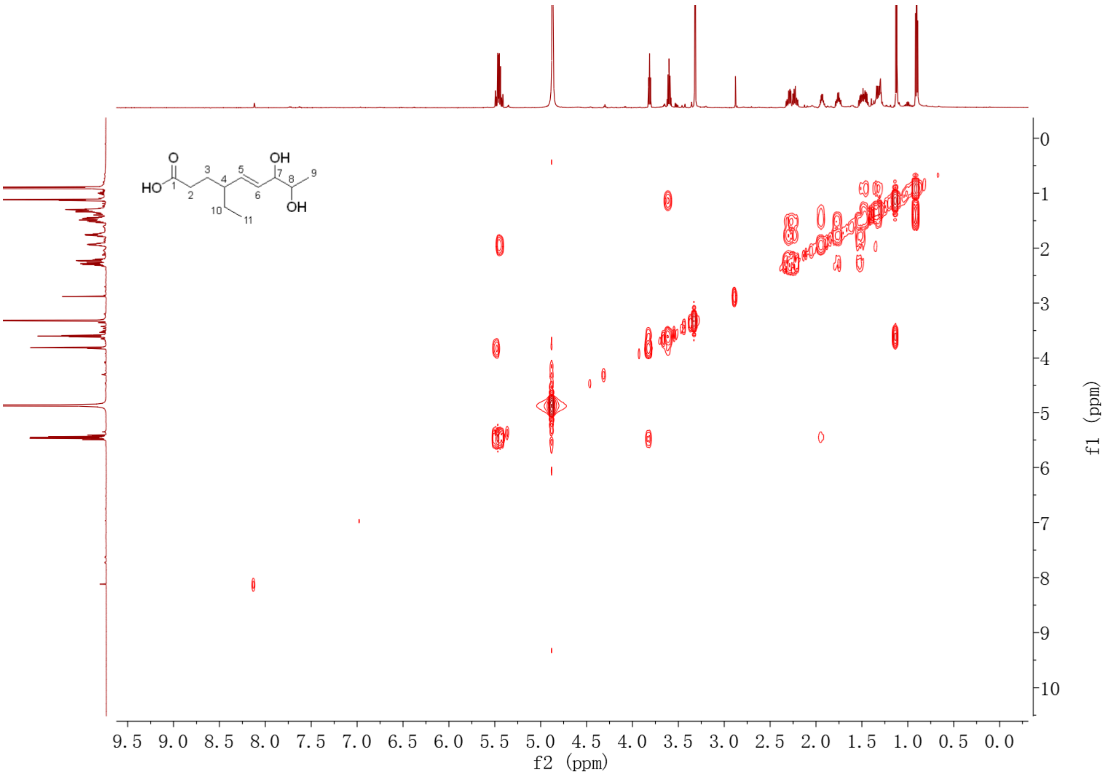

Figure S2f. ^{1}H-^{1}H COSY spectrum of **2** in CD3OD.

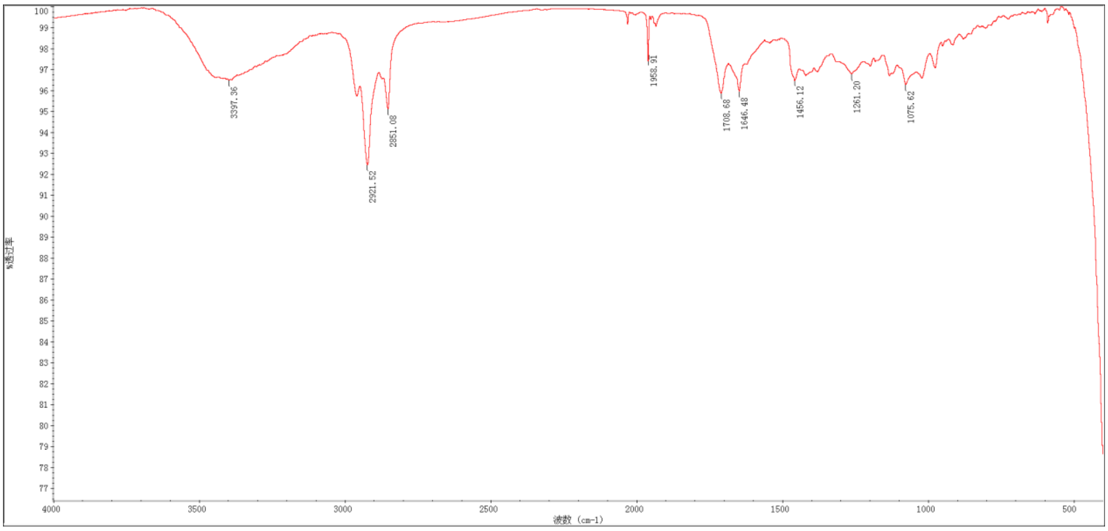

Figure S2g. The IR (KBr disc) spectrum of **2.**

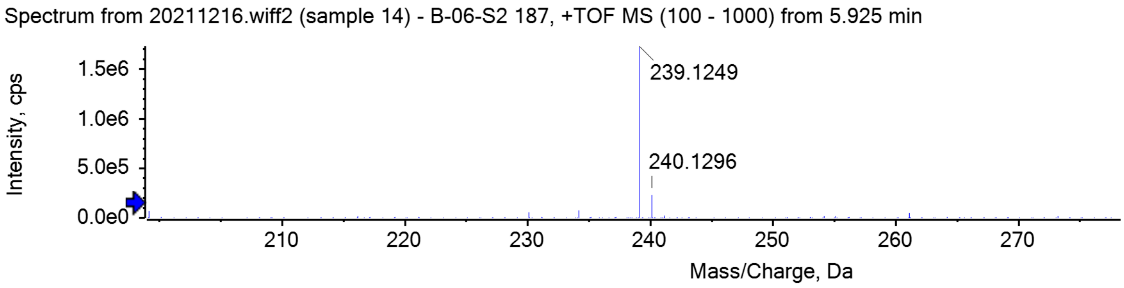

Formula (M)	Ion Formula	m/z	Calc m/z	Diff (ppm)	DBE
$C_{11}H_{20}O_4$	$C_{11}H_{20}NaO_4$	239.1249	239.1254	-2.0	2

Figure S2h. The HR-ESI-MS spectroscopic data of **2**

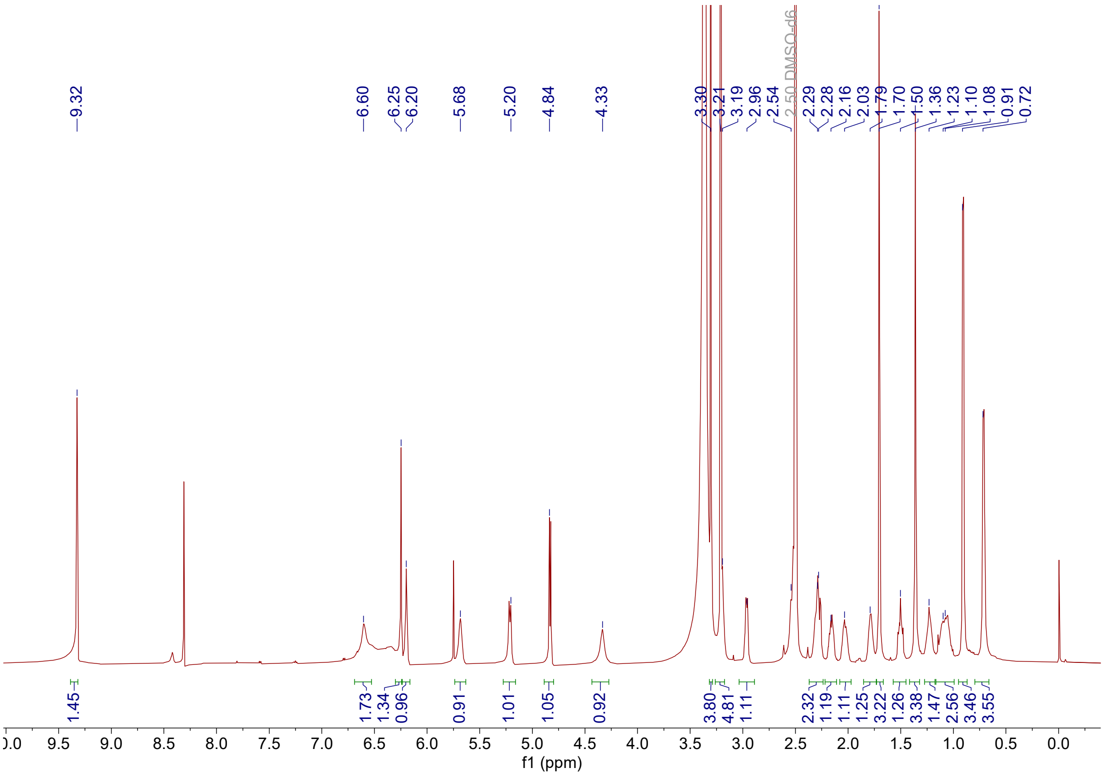

Figure S3a. ^{1}H NMR spectrum of **3** in DMSO.

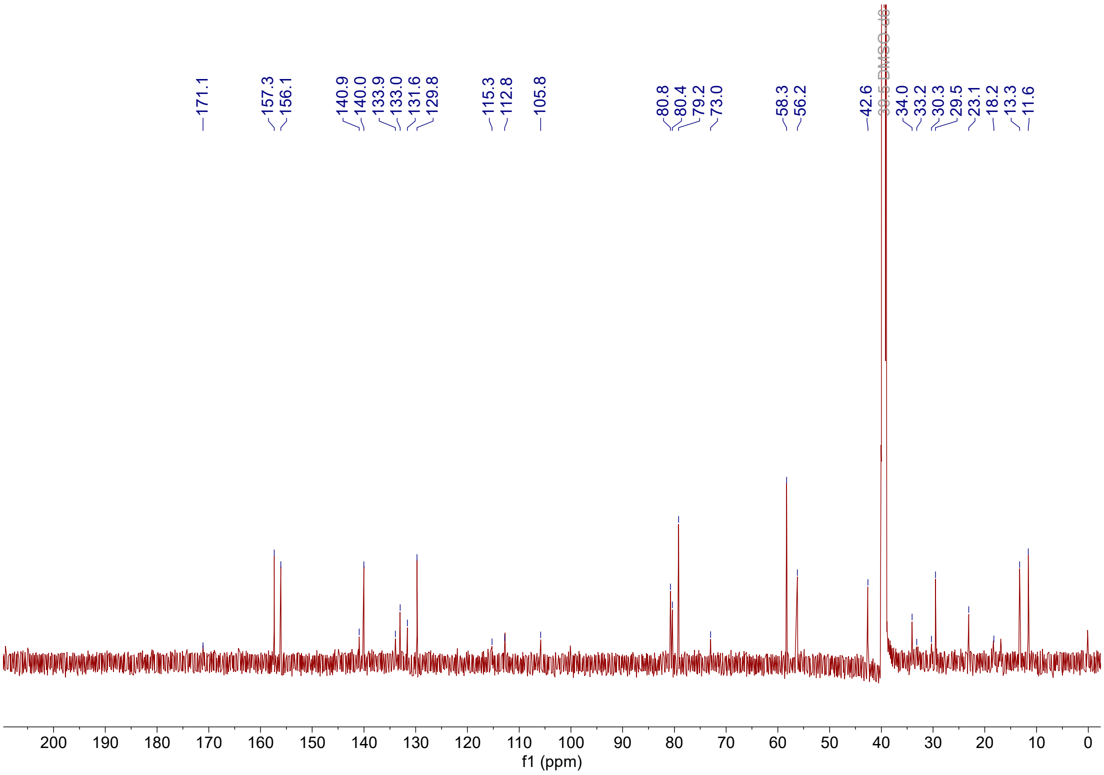

Figure S3b. ^{13}C NMR spectrum of **3** in DMSO.

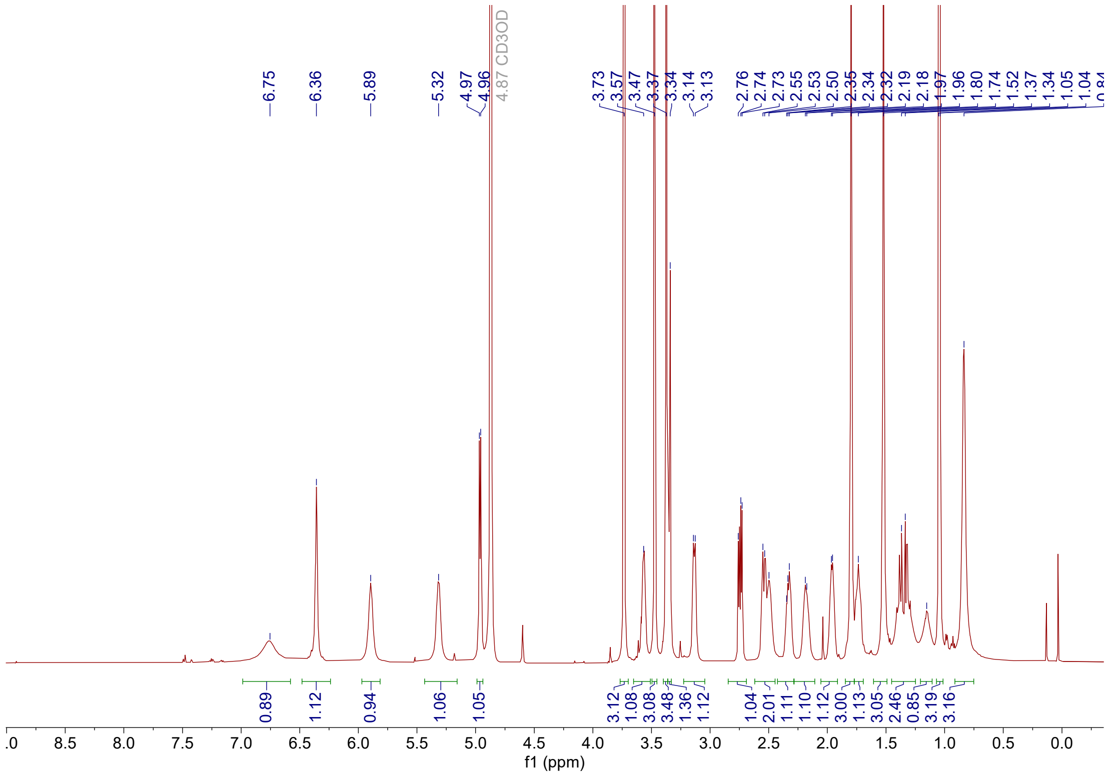

Figure S4a. ^{1}H NMR spectrum of **4** in CD_3OD.

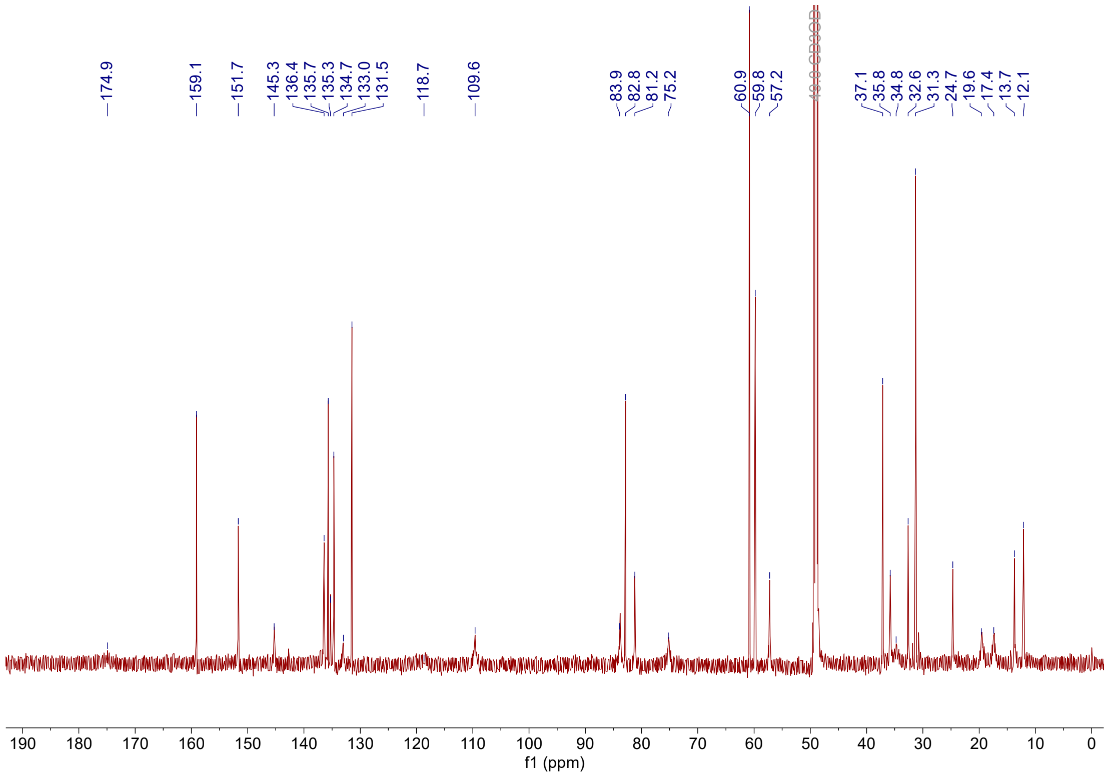

Figure S4b. ^{13}C NMR spectrum of **4** in CD_3OD.

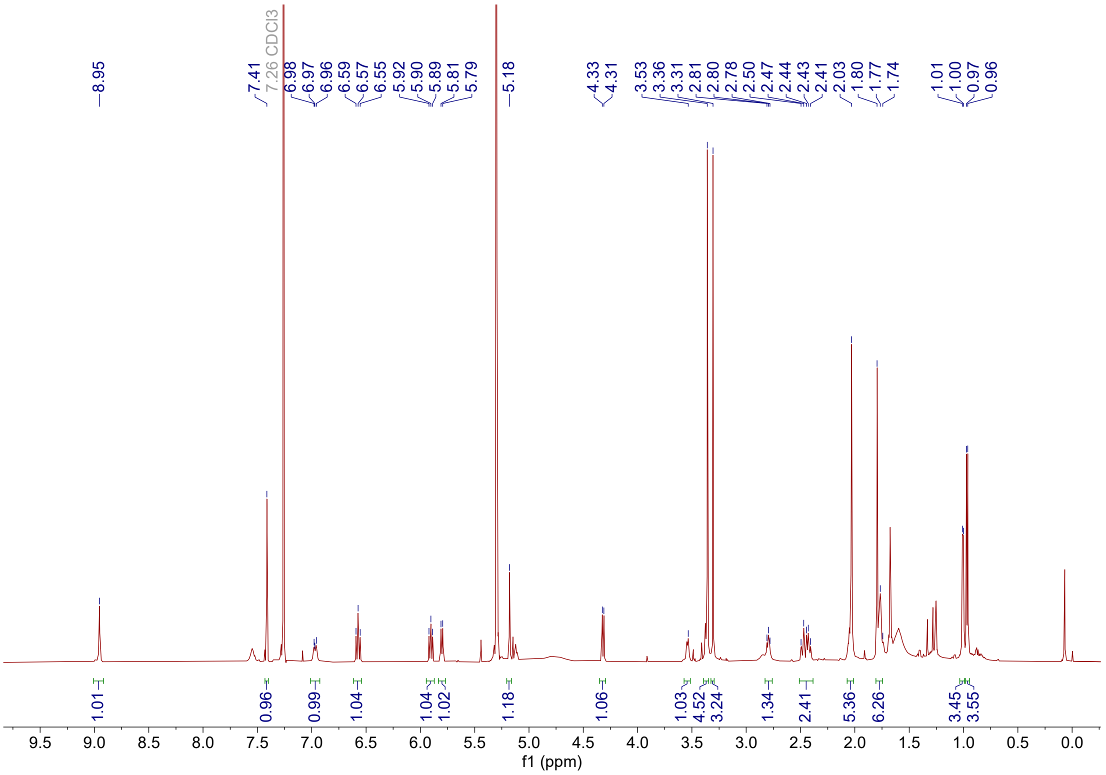

Figure S5a. ^{1}H NMR spectrum of **5** in $CDCl_3$.

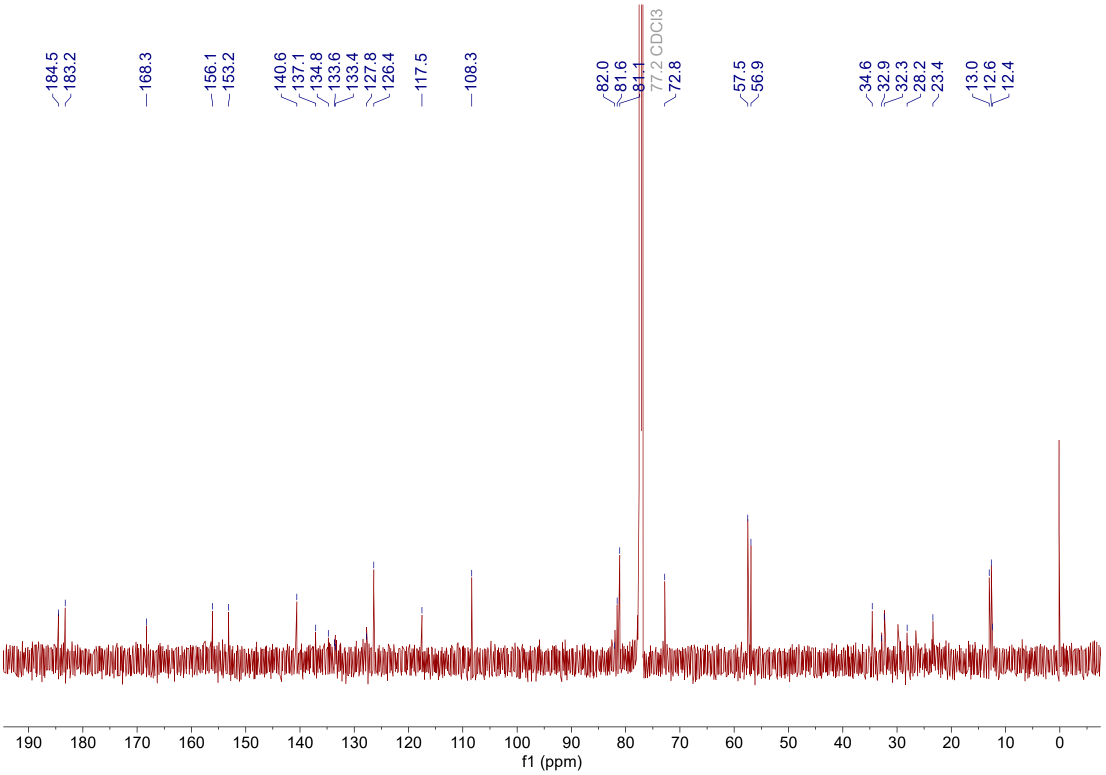

Figure S5b. ^{13}C NMR spectrum of **5** in $CDCl_3$.

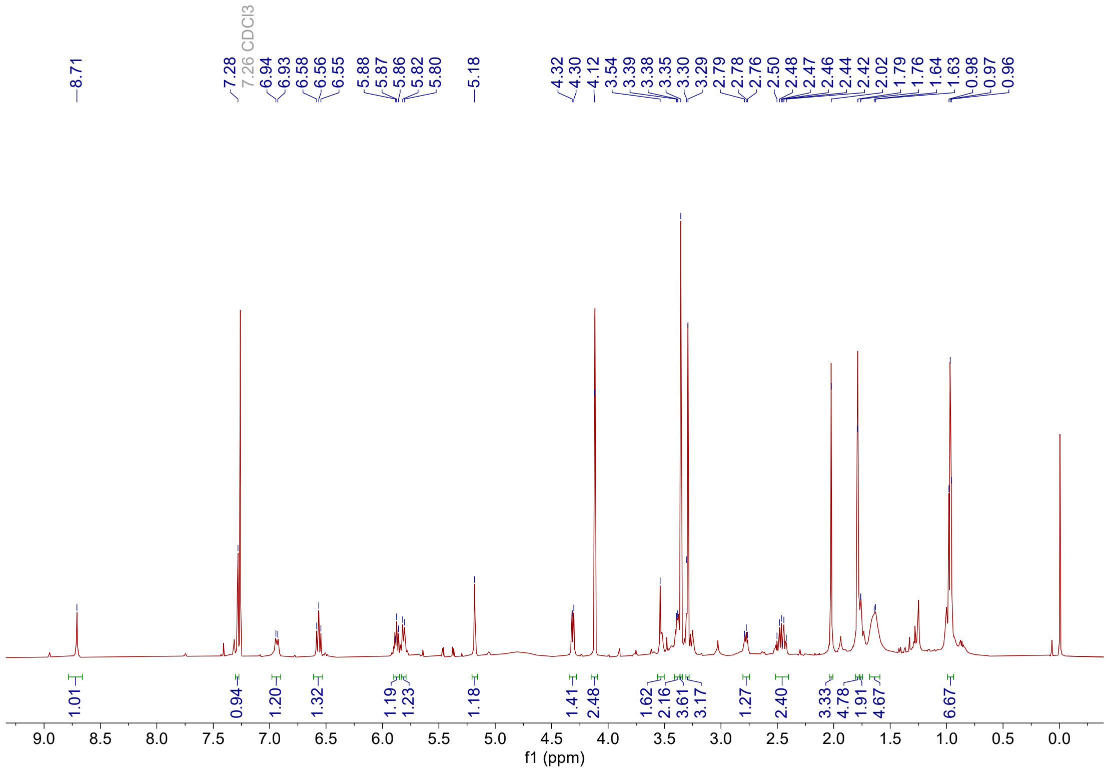

Figure S6a. ^{1}H NMR spectrum of **6** in $CDCl_3$.

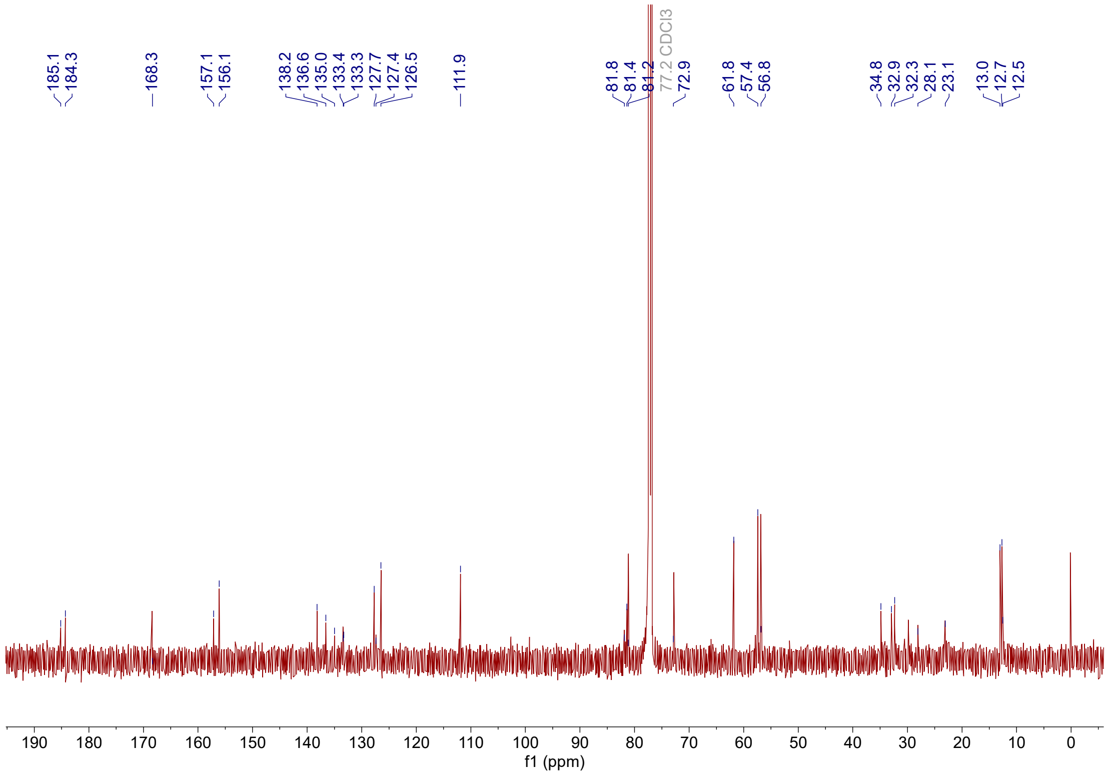

Figure S6b. ^{13}C NMR spectrum of **6** in $CDCl_3$.

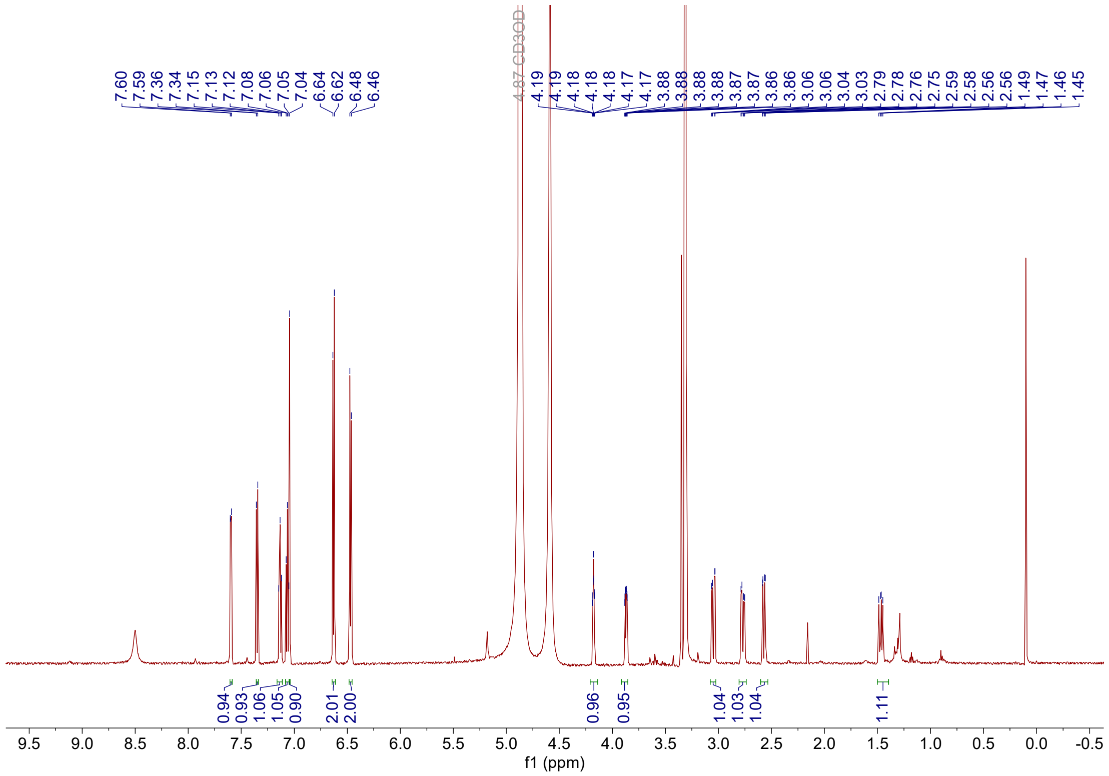

Figure S7a. ^{1}H NMR spectrum of **7** in CD_3OD.

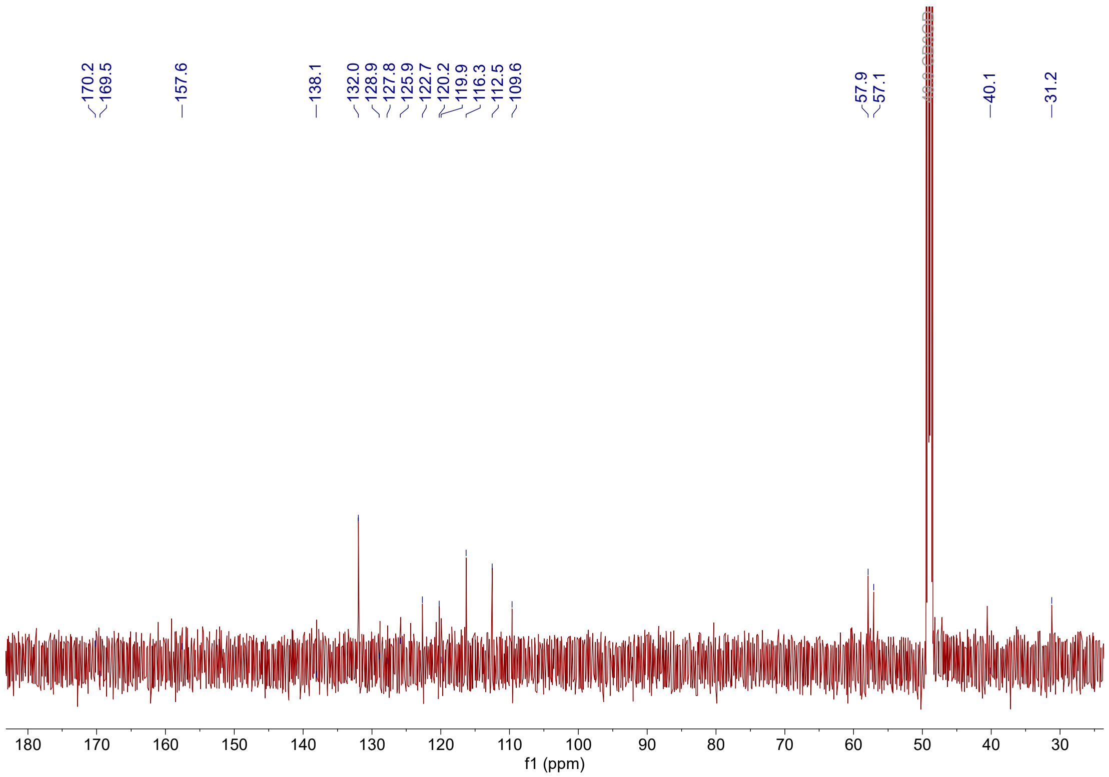

Figure S7b. ^{13}C NMR spectrum of **7** in CD_3OD.

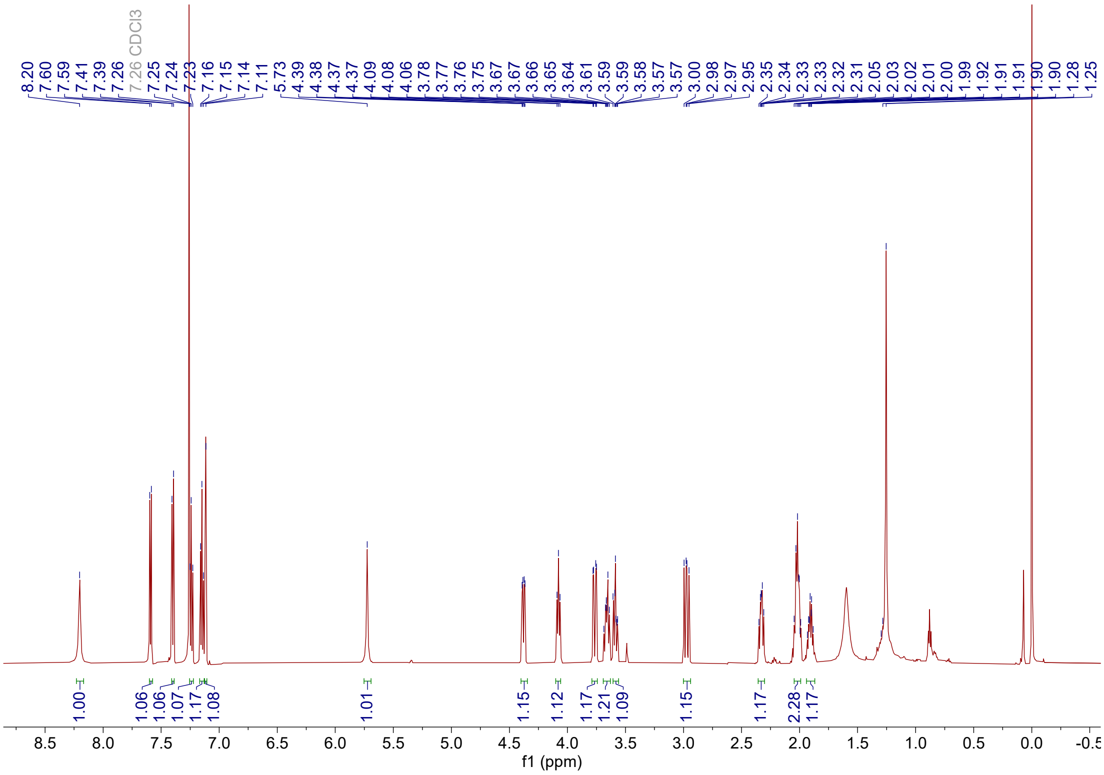

Figure S8a. ^{1}H NMR spectrum of **8** in $CDCl_3$.

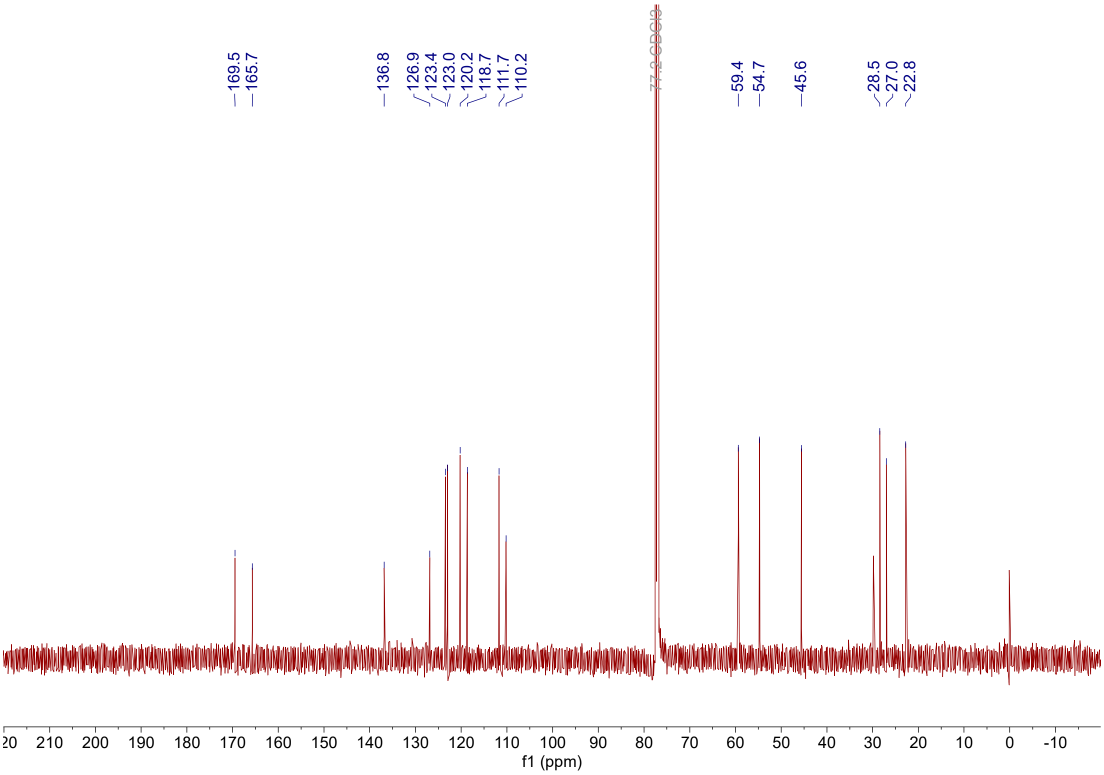

Figure S8b. ^{13}C NMR spectrum of **8** in $CDCl_3$.

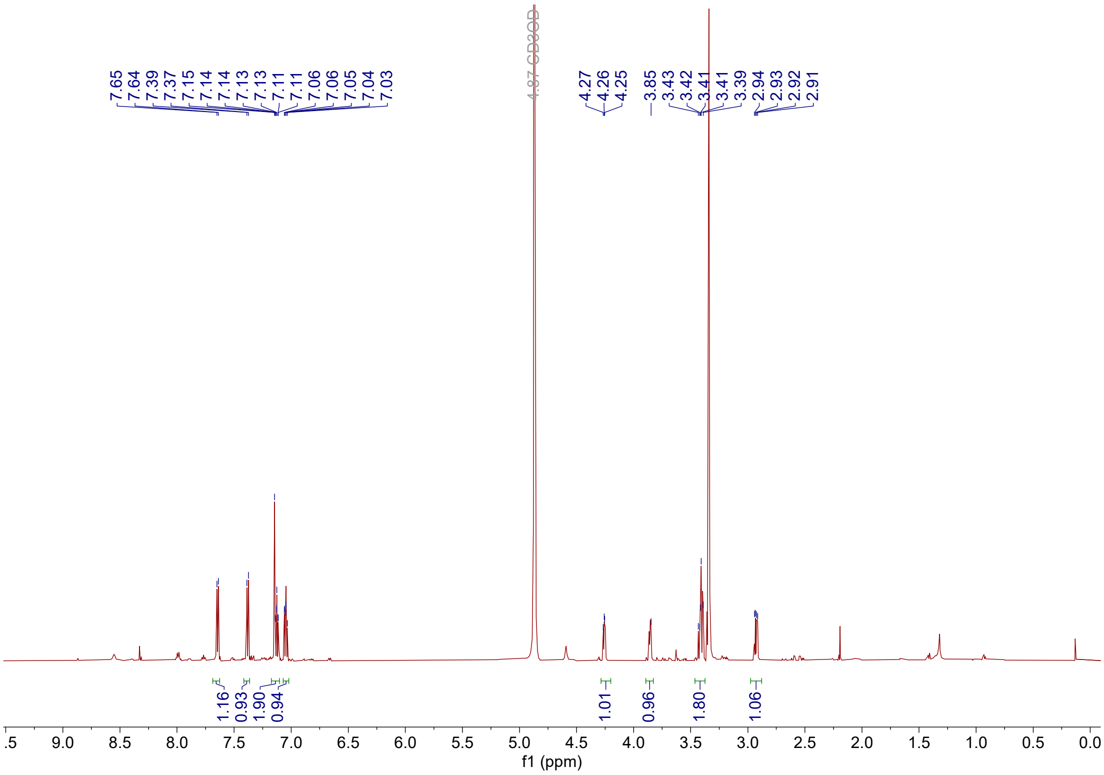

Figure S9a. ^{1}H NMR spectrum of **9** in CD_3OD.

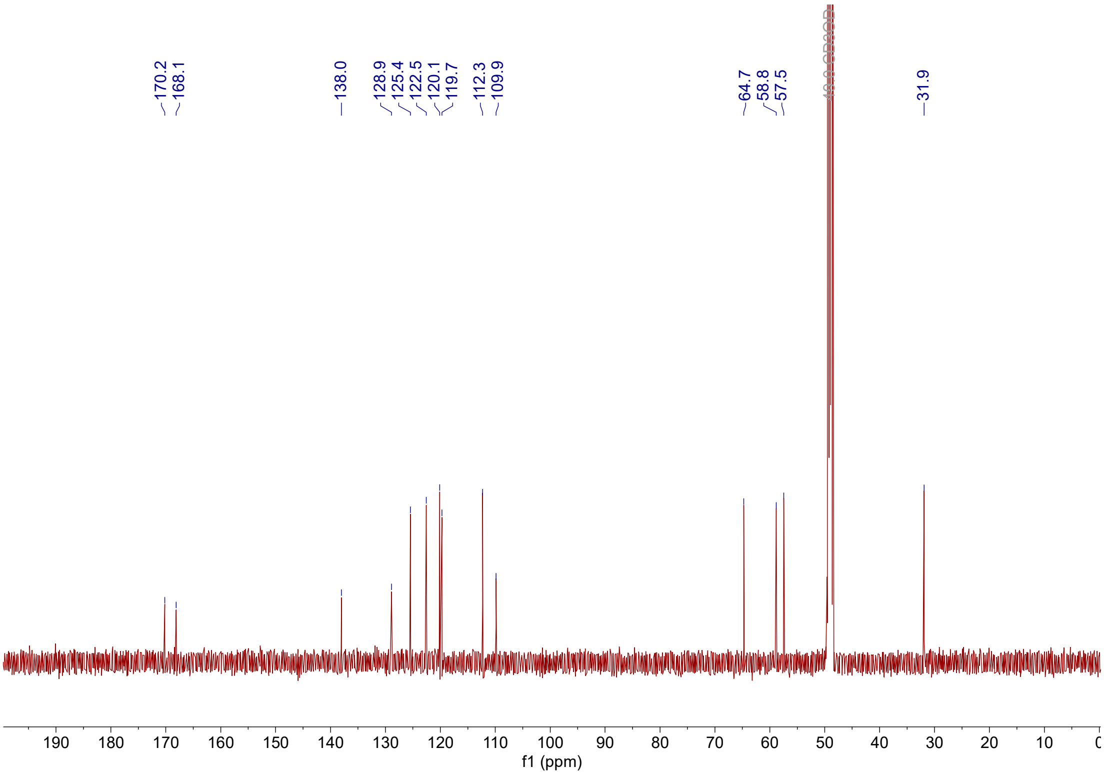

Figure S9b. ^{13}C NMR spectrum of **9** in CD_3OD.

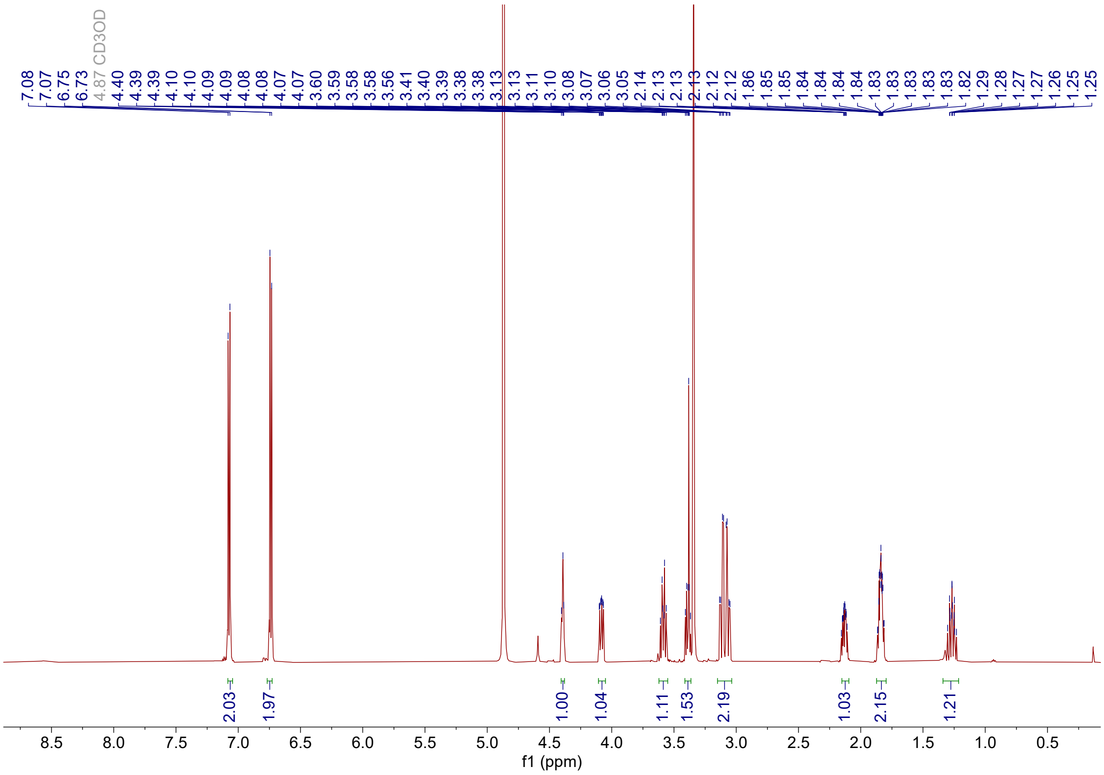

Figure S10a. ^{1}H NMR spectrum of **10** in CD_3OD.

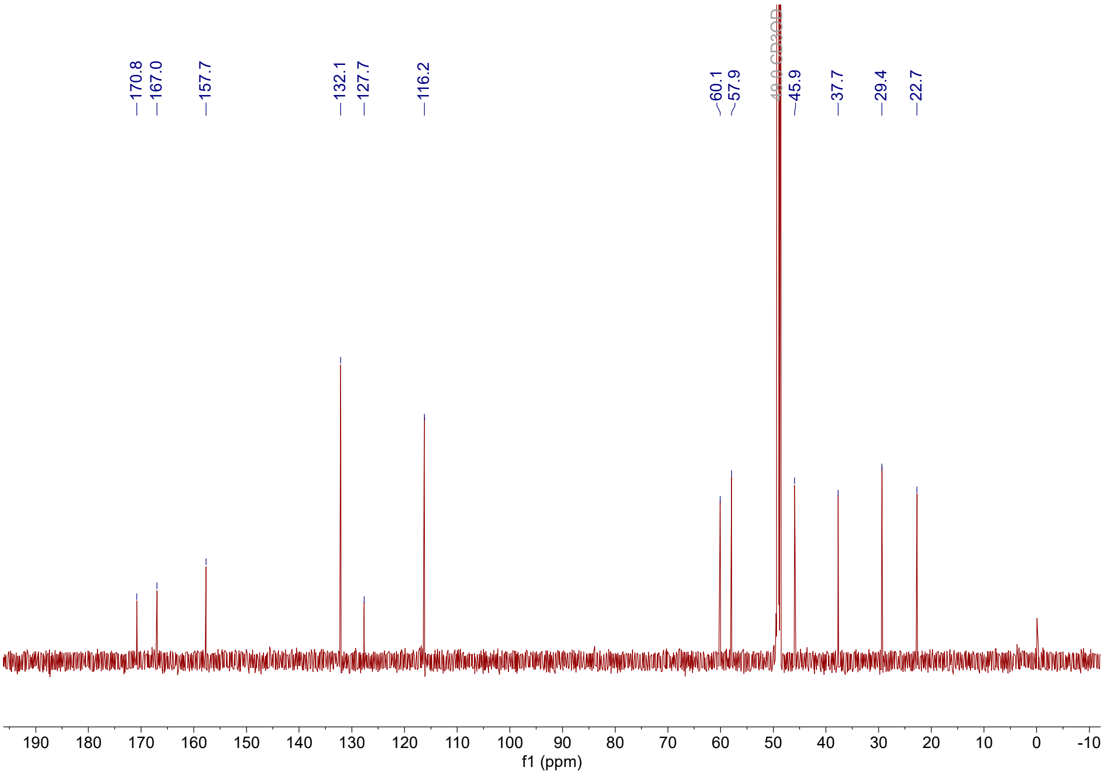

Figure S10b. ^{13}C NMR spectrum of **10** in CD_3OD.

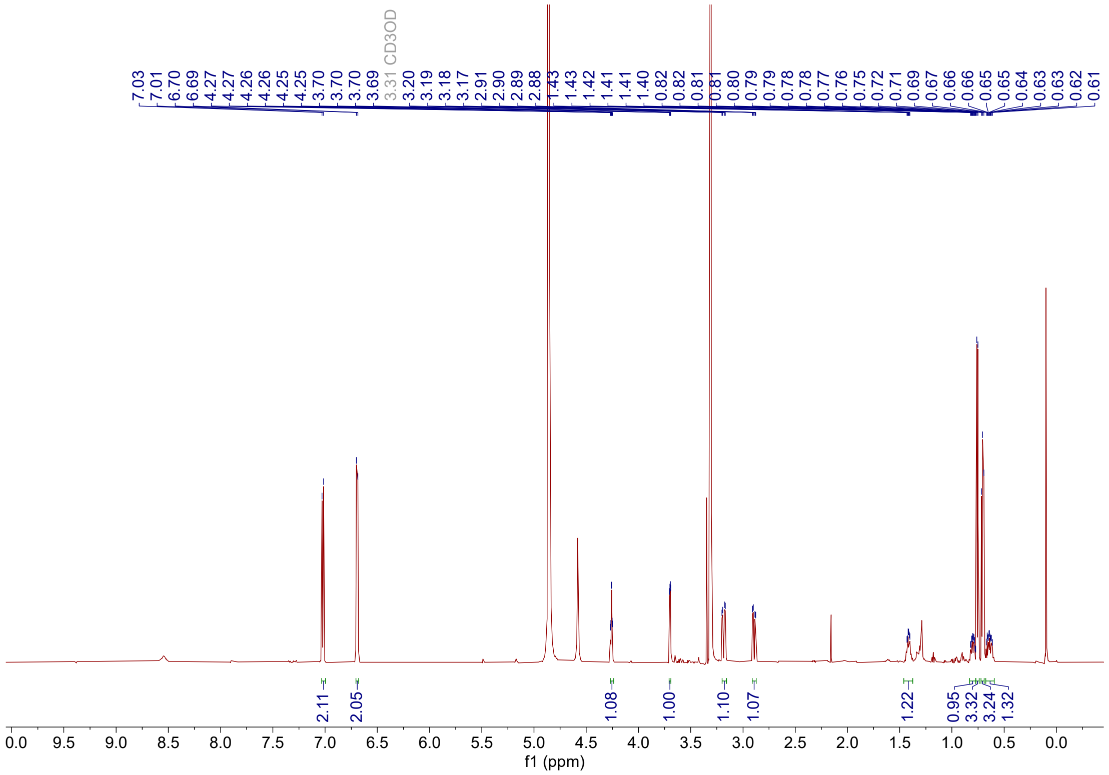

Figure S11a. ^{1}H NMR spectrum of **11** in CD_3OD.

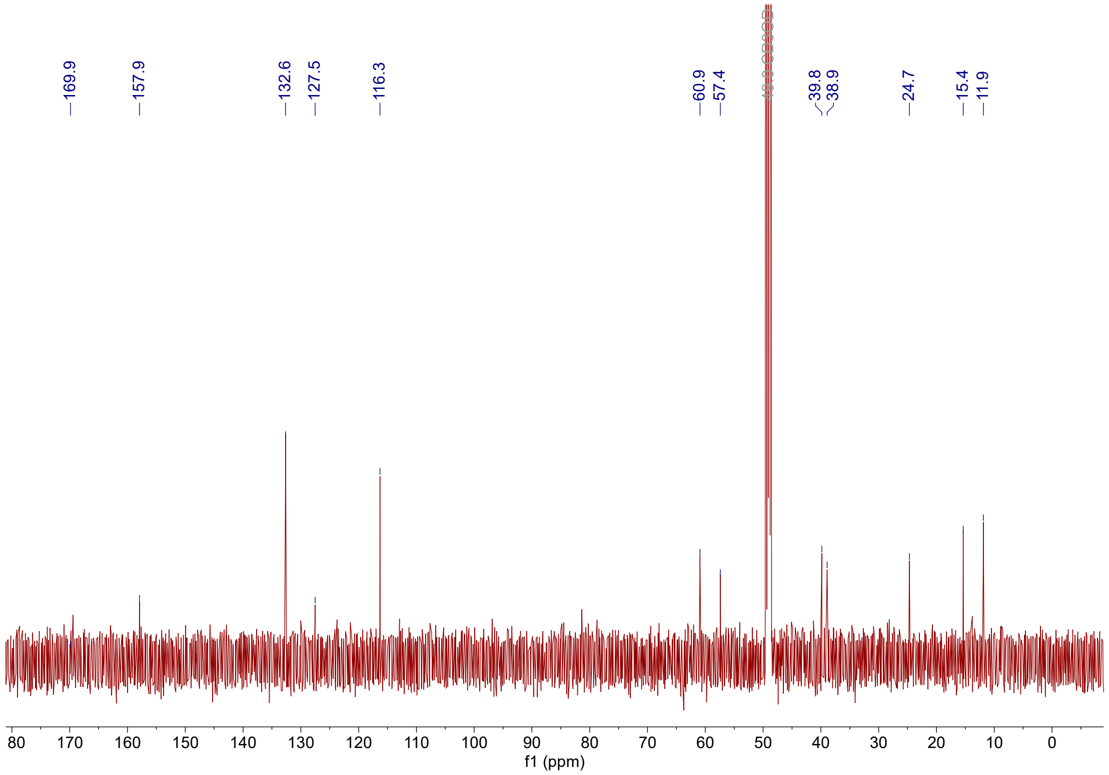

Figure S11b. ^{13}C NMR spectrum of **11** in CD_3OD.

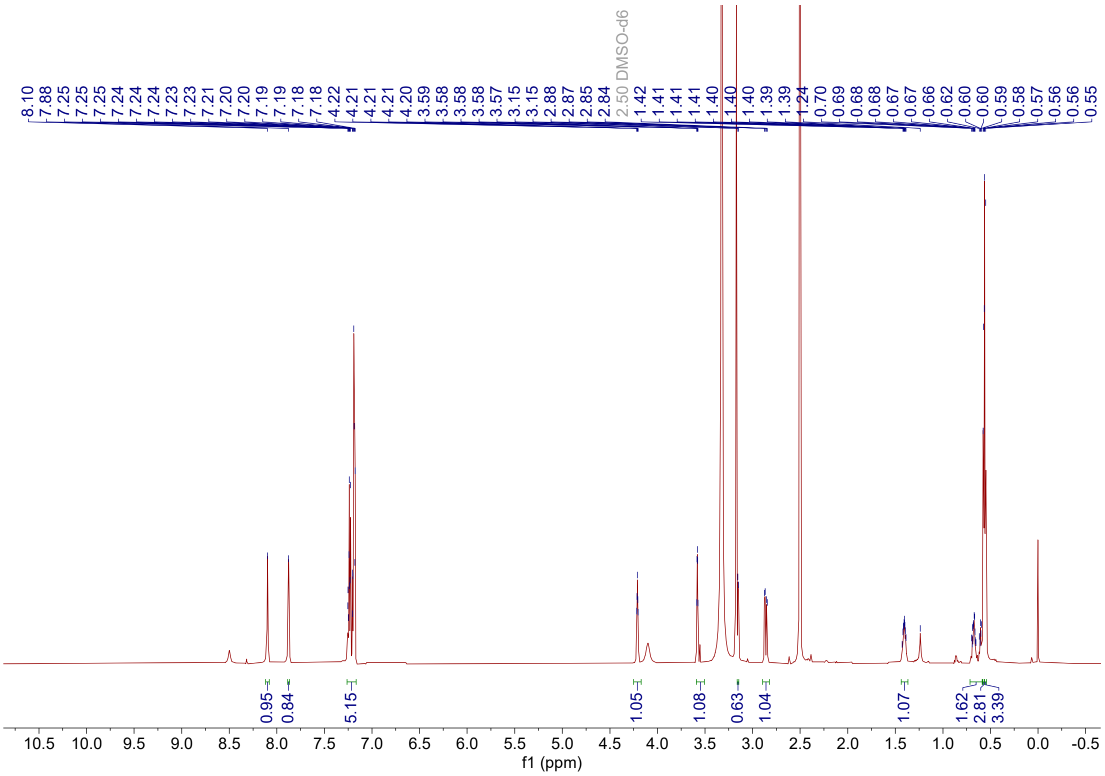

Figure S12a. ^{1}H NMR spectrum of **12** in DMSO.

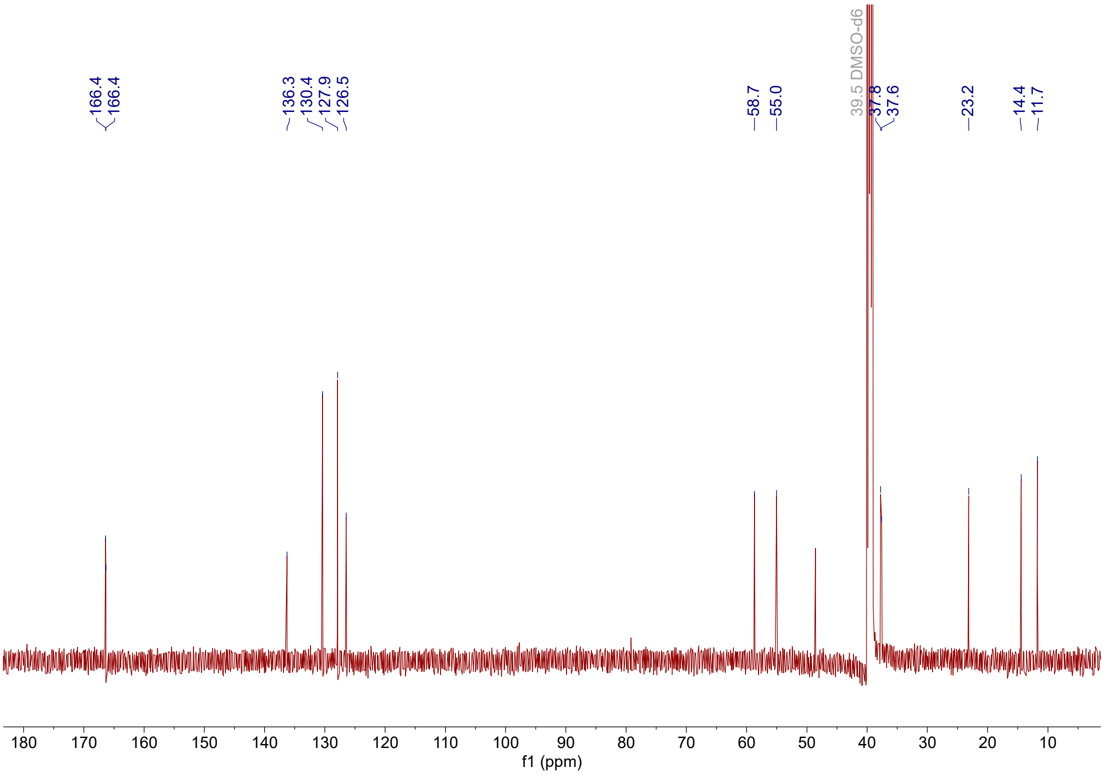

Figure S12b. ^{13}C NMR spectrum of **12** in DMSO.

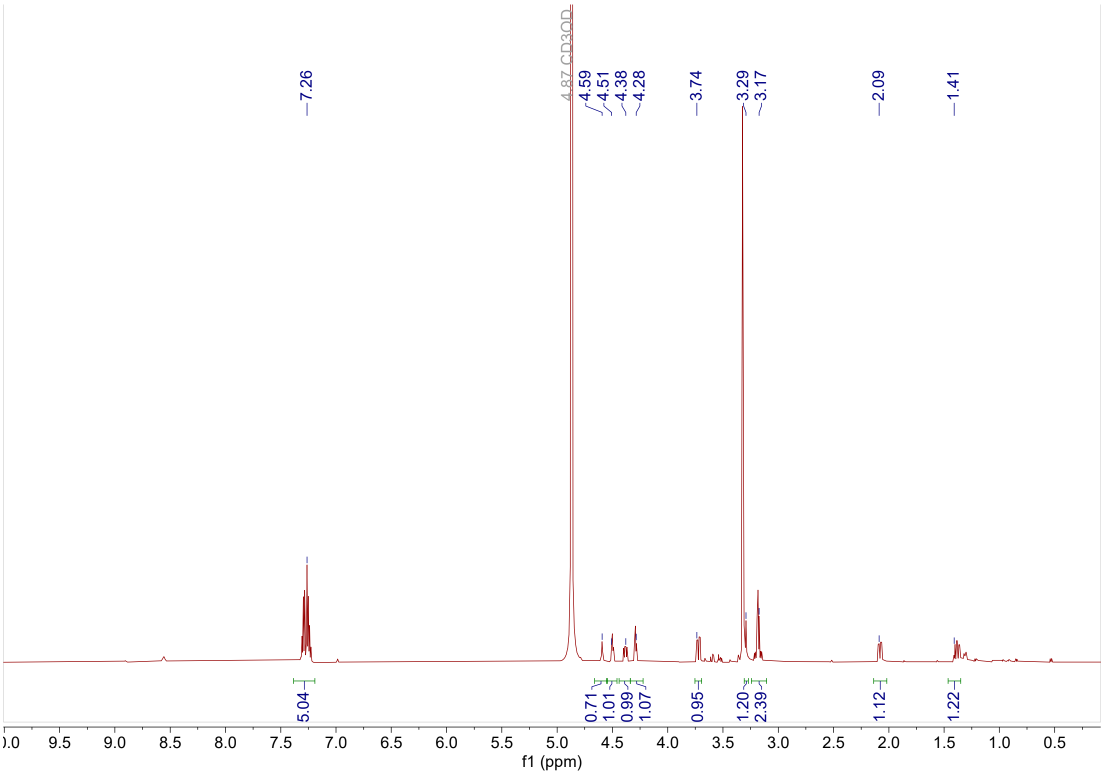

Figure S13a. ^{1}H NMR spectrum of **13** in CD_3OD.

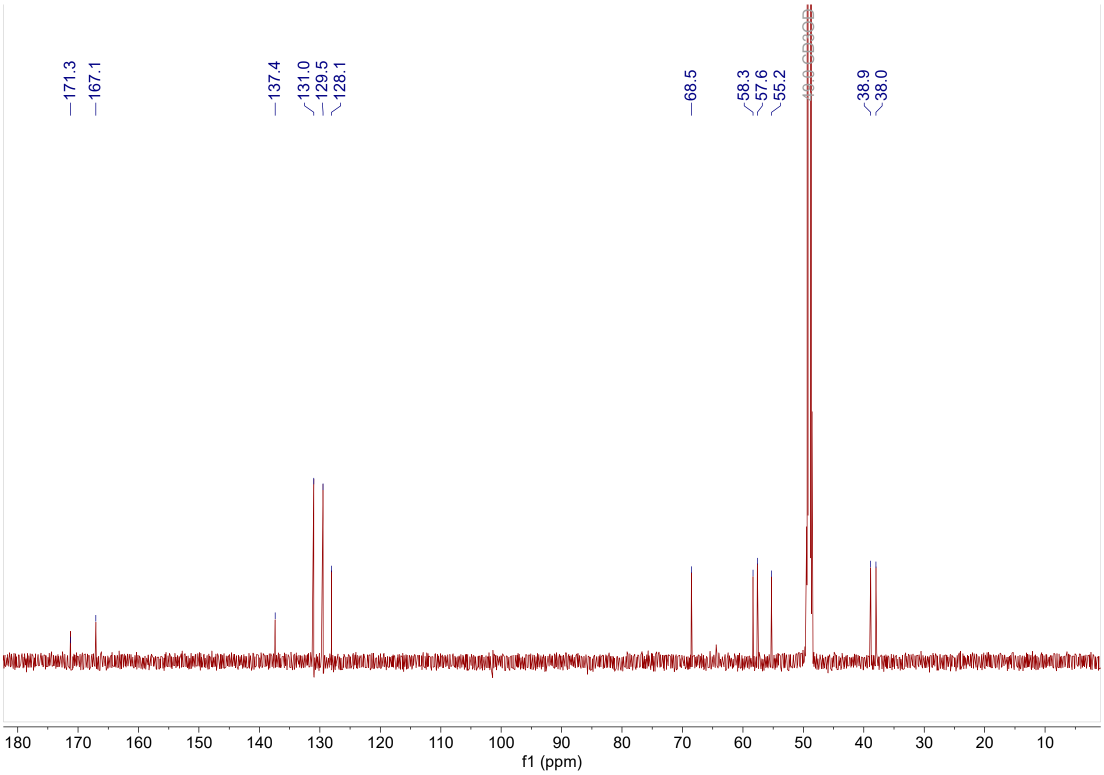

Figure S13b. ^{13}C NMR spectrum of **13** in CD_3OD.

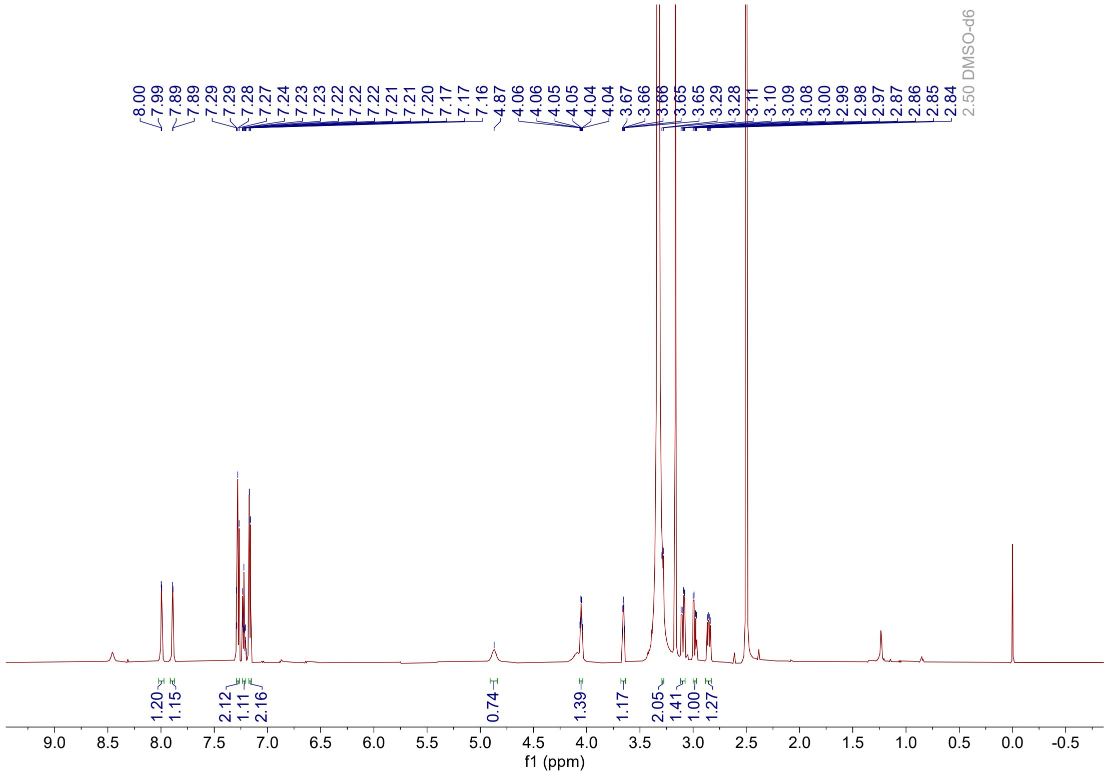

Figure S14a. ^{1}H NMR spectrum of **14** in DMSO.

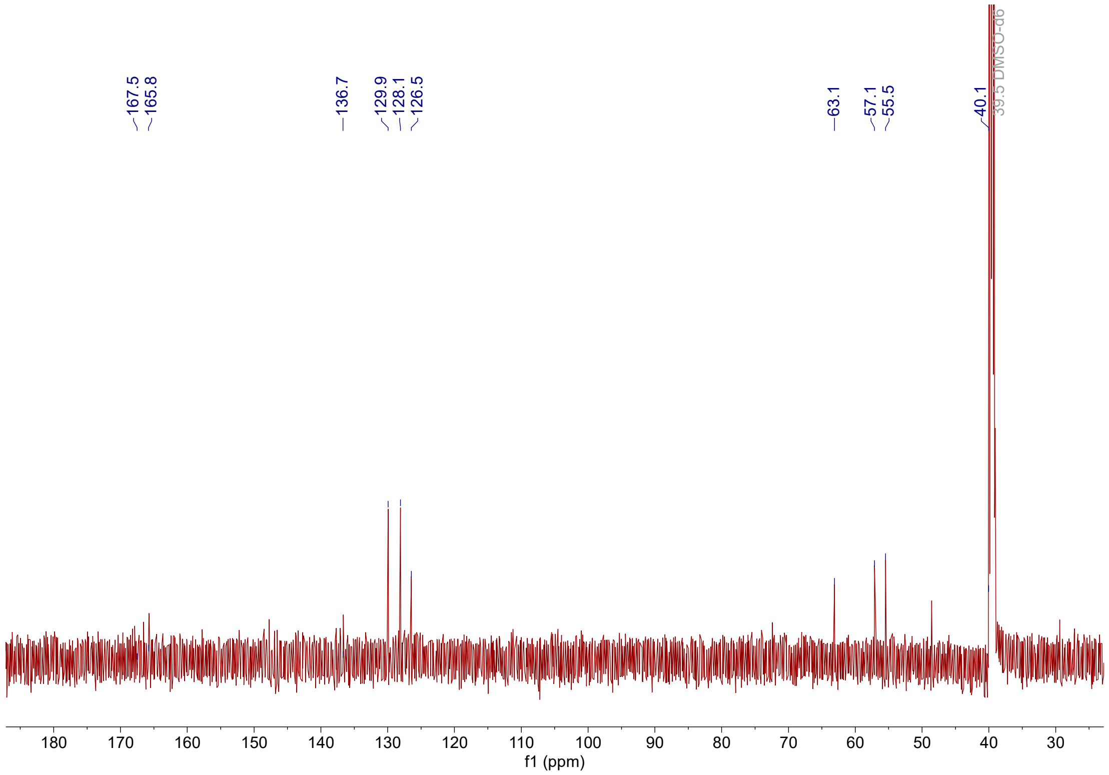

Figure S14b. ^{13}C NMR spectrum of **14** in DMSO.

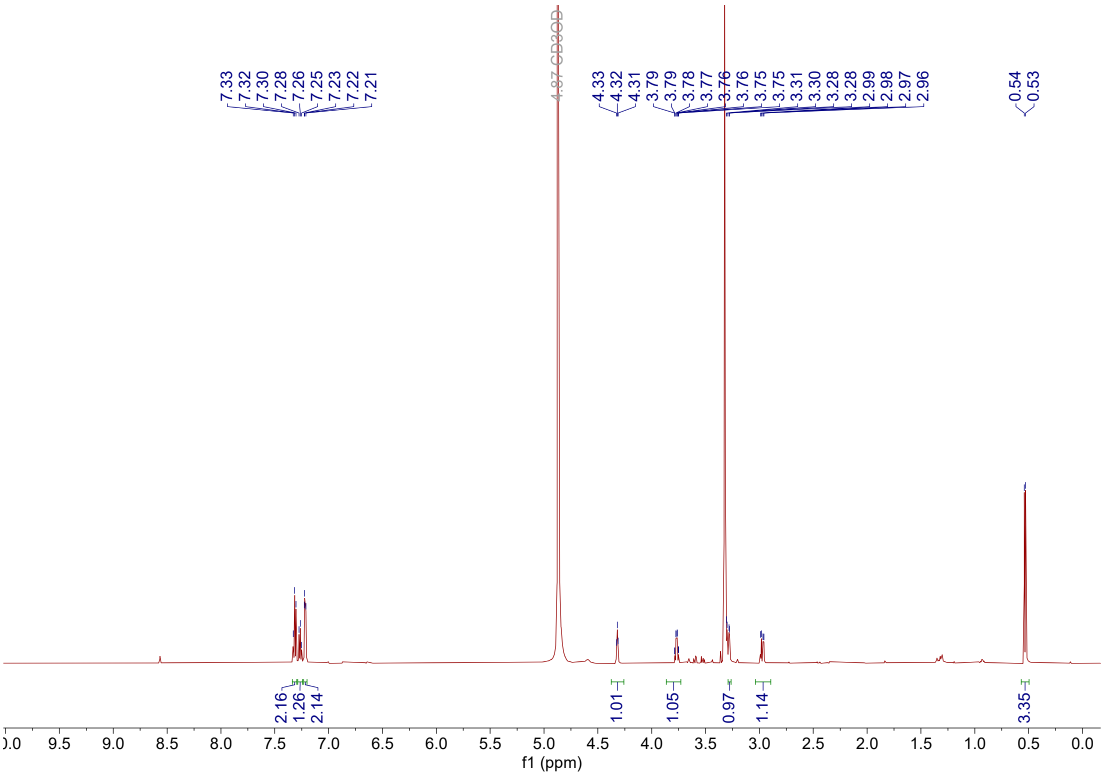

Figure S15a. ^{1}H NMR spectrum of **15** in CD_3OD.

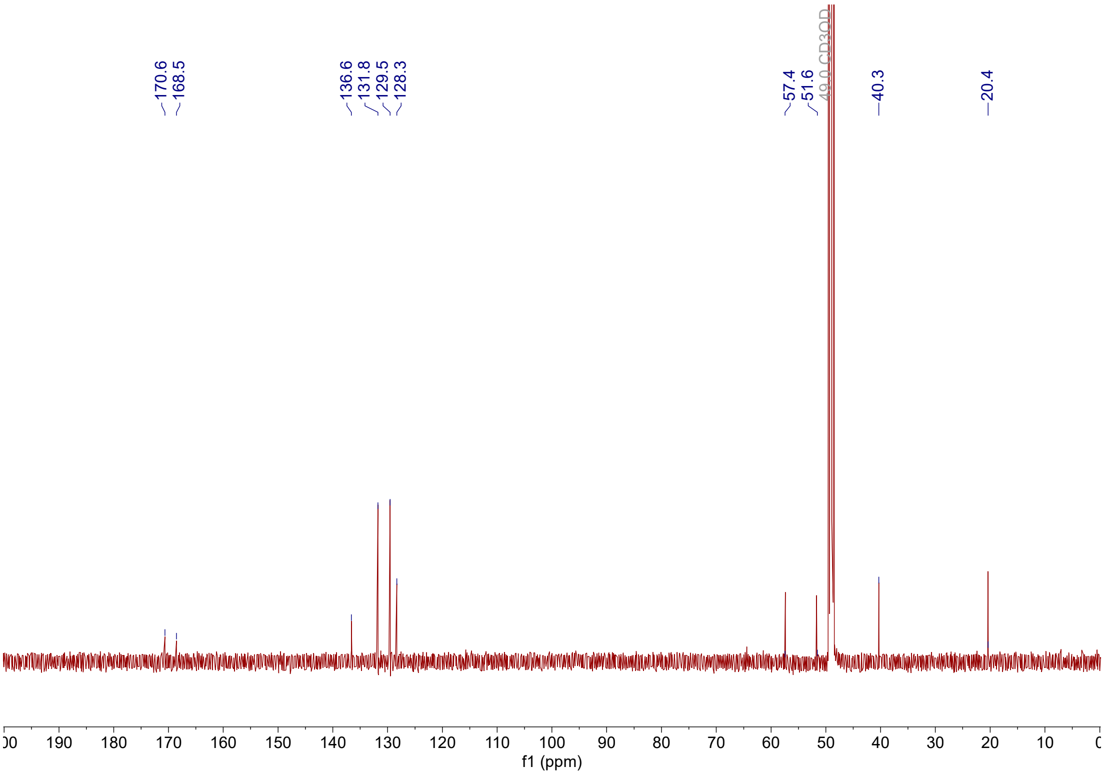

Figure S15b. ^{13}C NMR spectrum of **15** in CD_3OD.

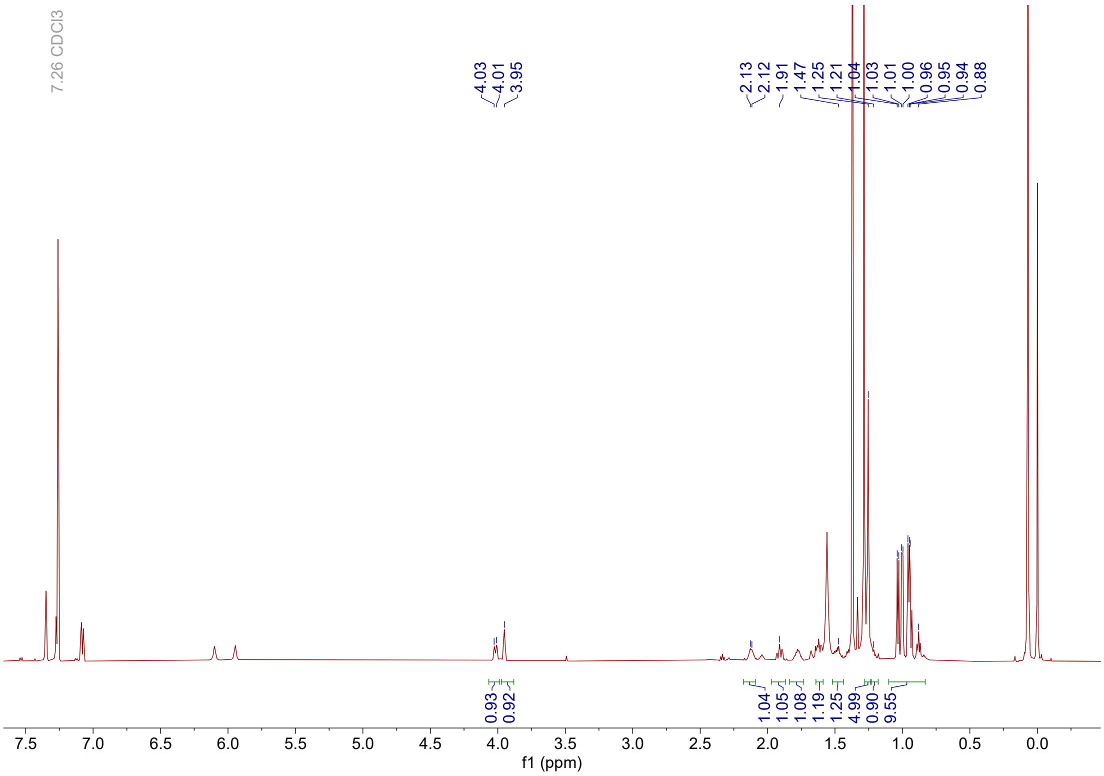

Figure S16a. ^{1}H NMR spectrum of **16** in $CDCl_3$.

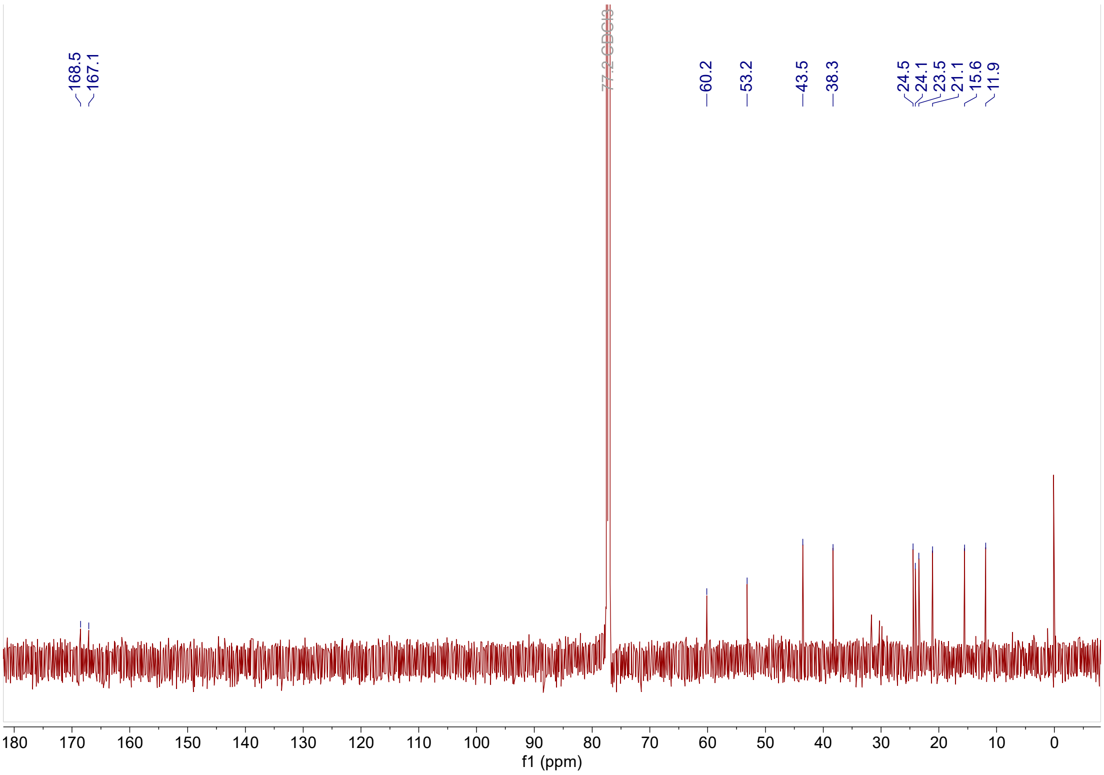

Figure S16b. ^{13}C NMR spectrum of **16** in $CDCl_3$.

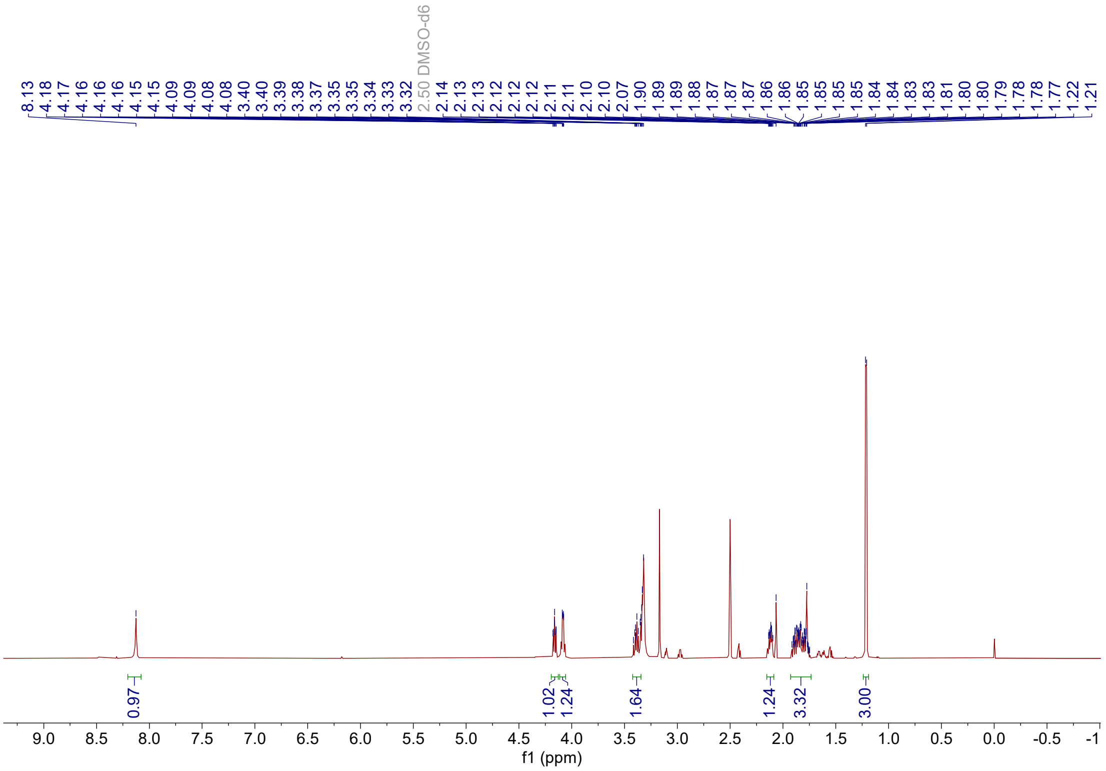

Figure S17a. ^{1}H NMR spectrum of **17** in DMSO.

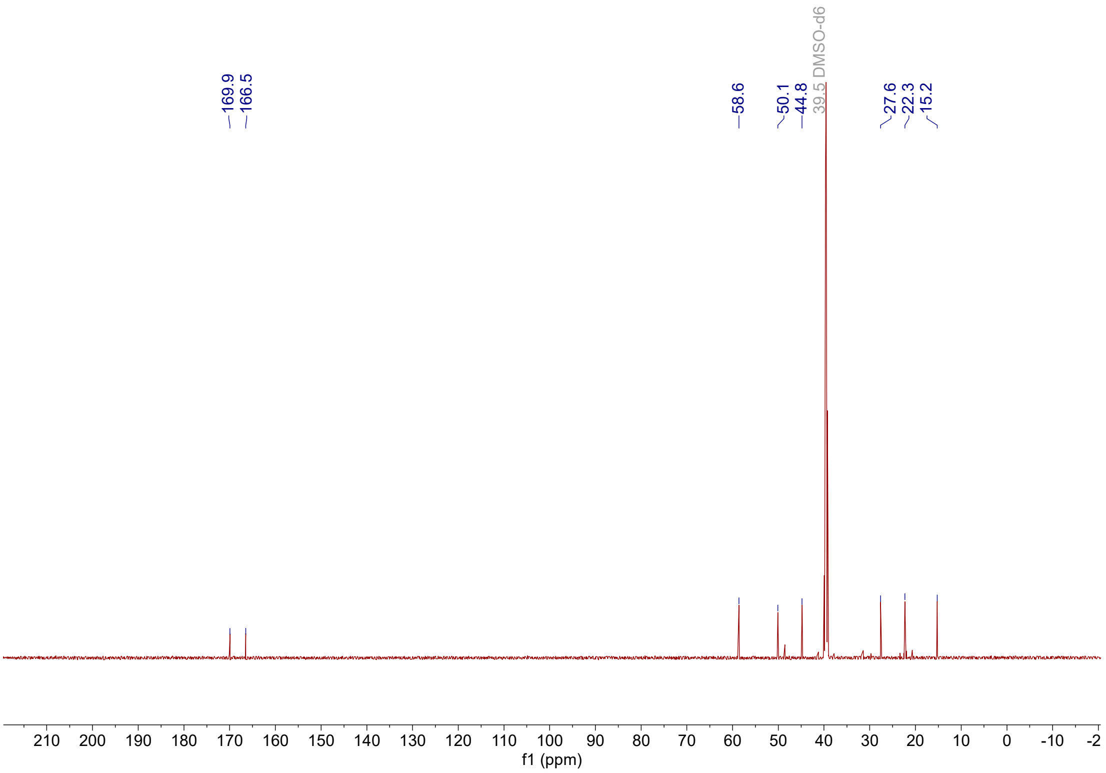

Figure S17b. ^{13}C NMR spectrum of **17** in DMSO.

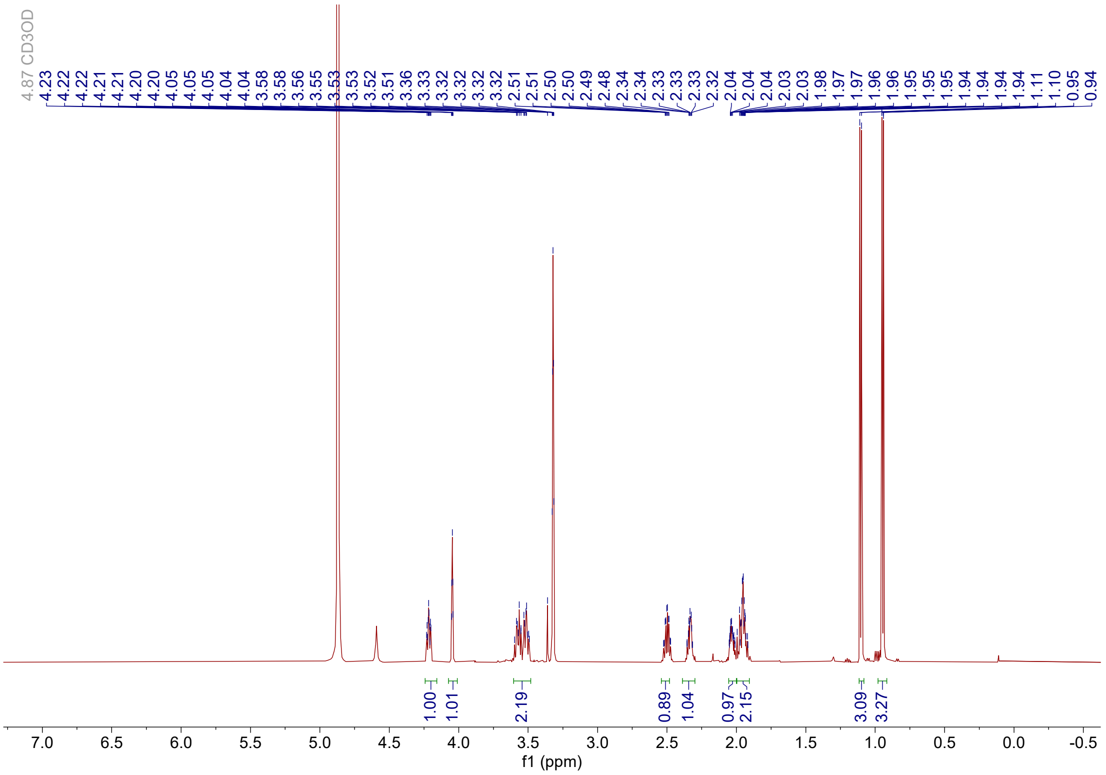

Figure S18a. ^{1}H NMR spectrum of **18** in CD_3OD.

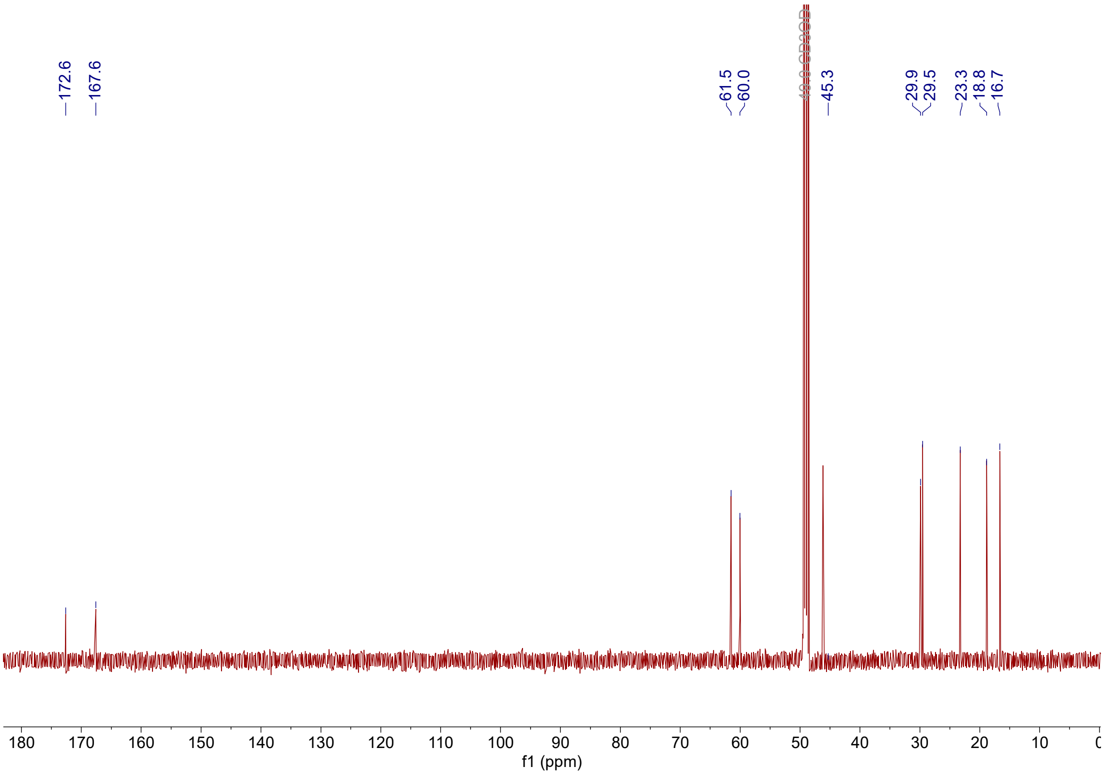

Figure S18b. ^{13}C NMR spectrum of **18** in CD_3OD.

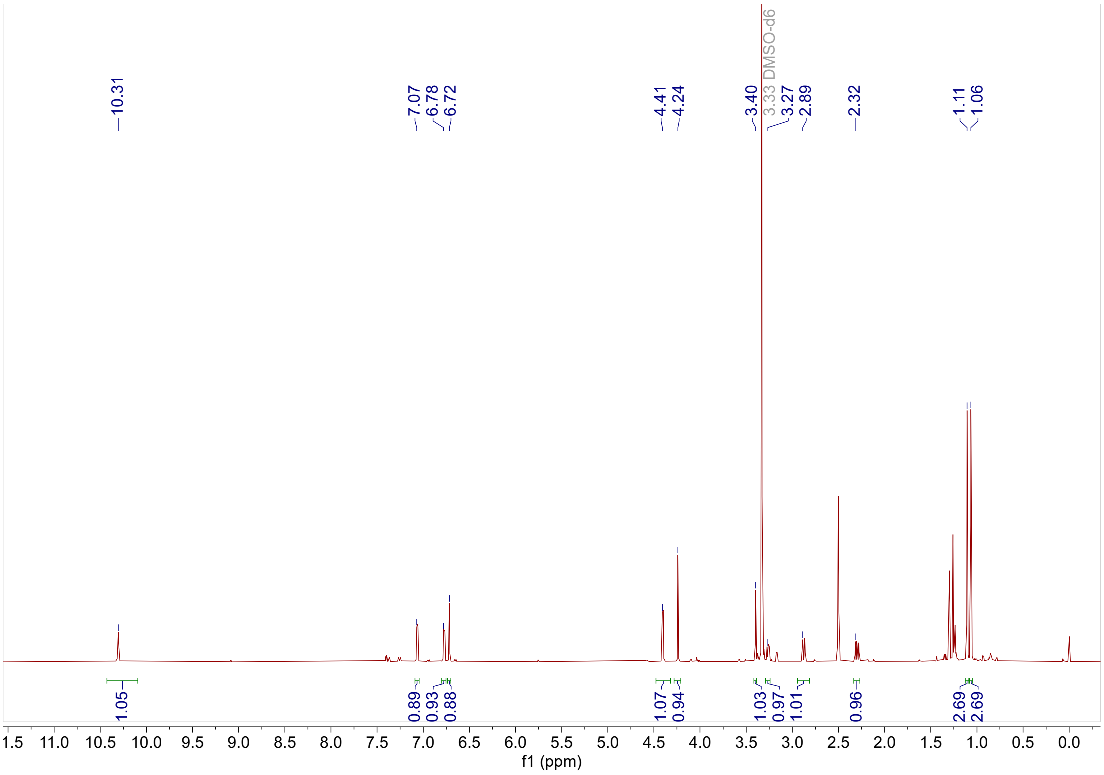

Figure S19a. ^{1}H NMR spectrum of **19** in DMSO.

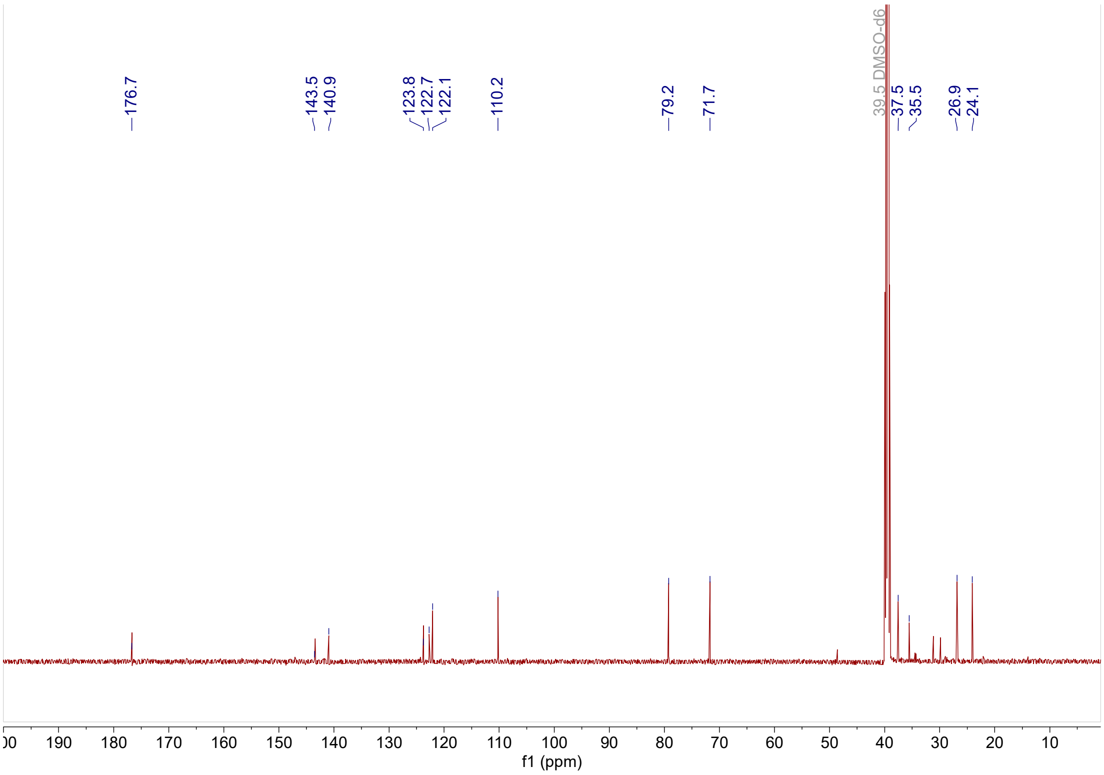

Figure S19b. ^{13}C NMR spectrum of **19** in DMSO.

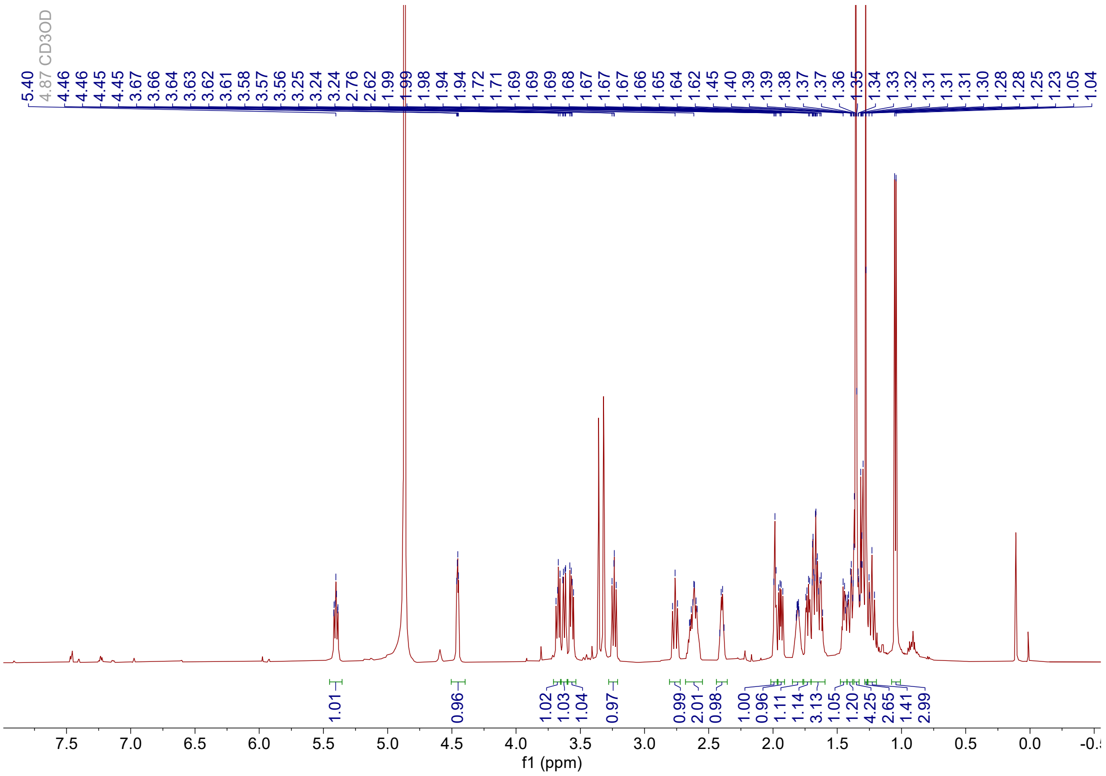

Figure S20a. ^{1}H NMR spectrum of **20** in CD_3OD.

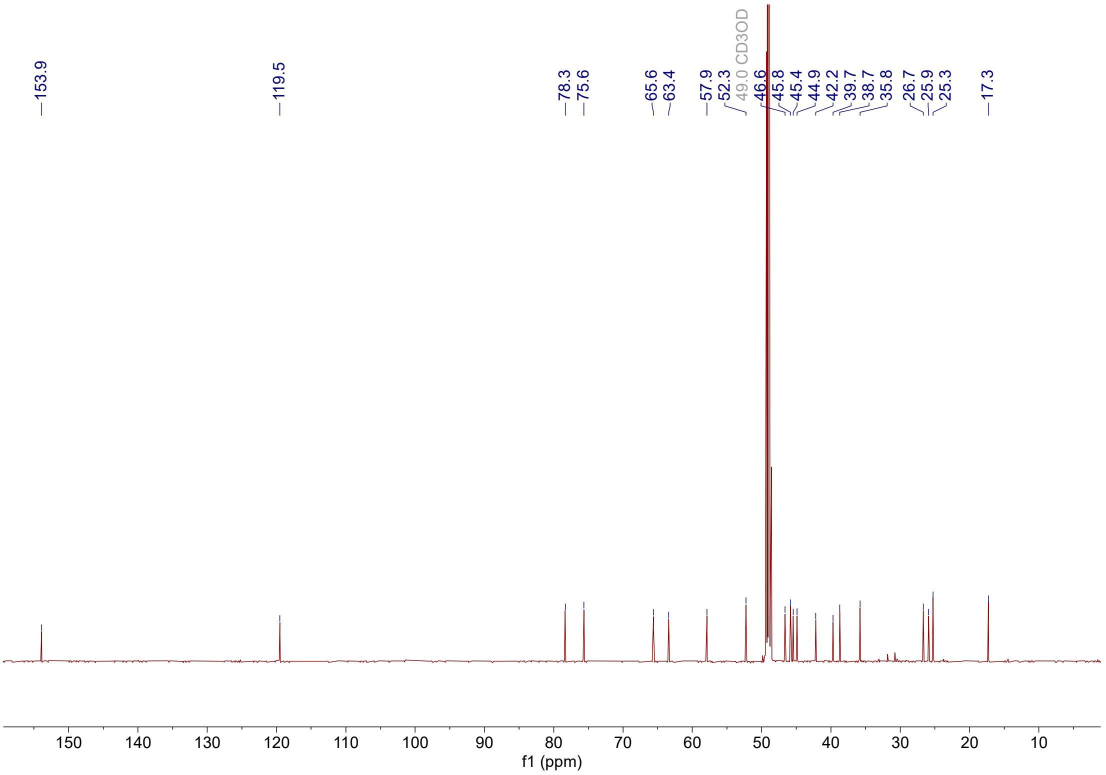

Figure S20b. ^{13}C NMR spectrum of **20** in CD_3OD.

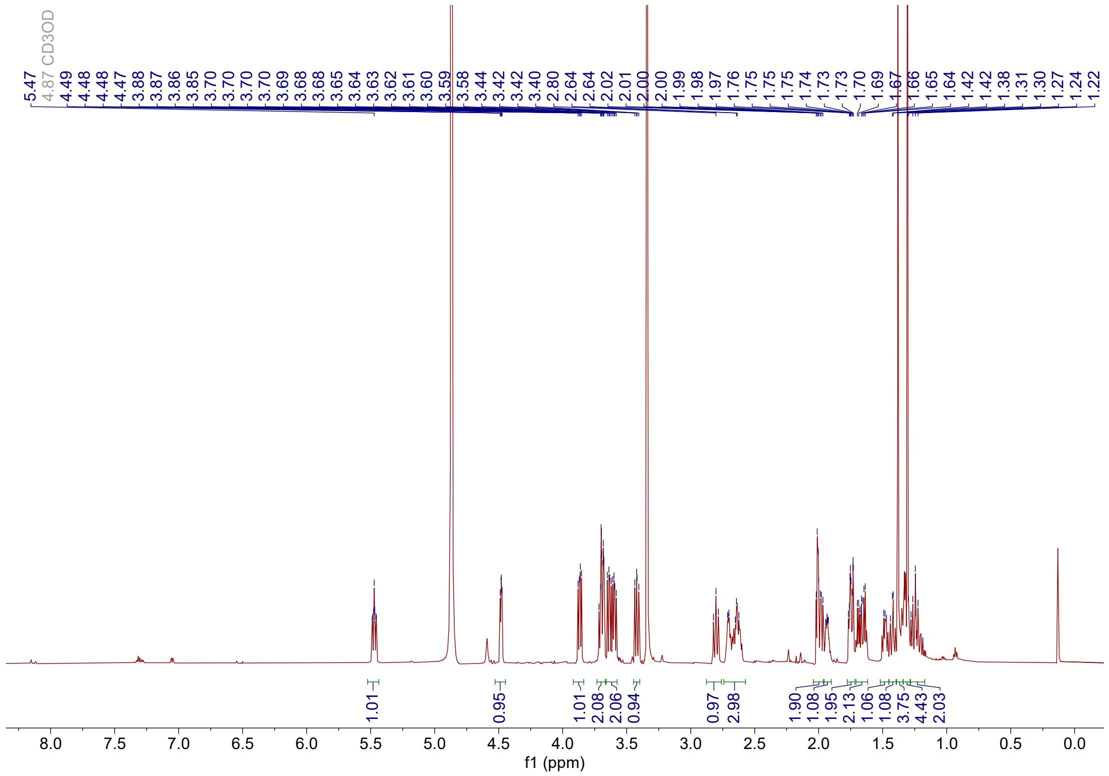

Figure S21a. ^{1}H NMR spectrum of **21** in CD_3OD.

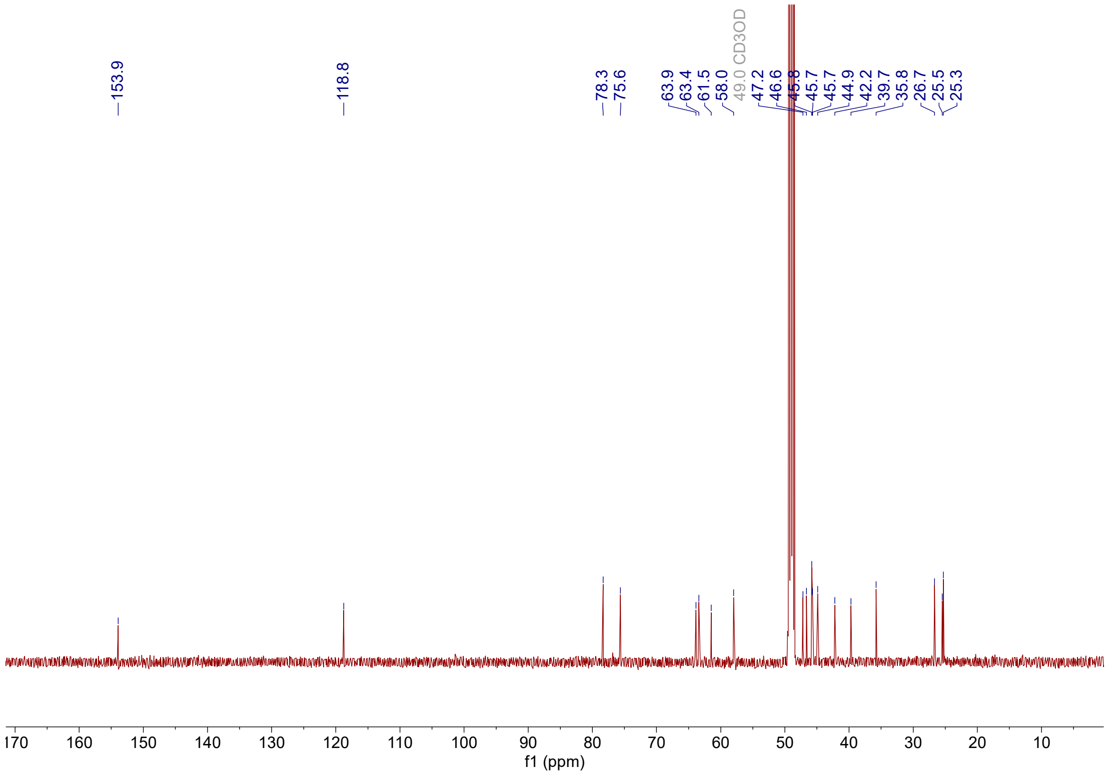

Figure S21b. ^{13}C NMR spectrum of **21** in CD_3OD.

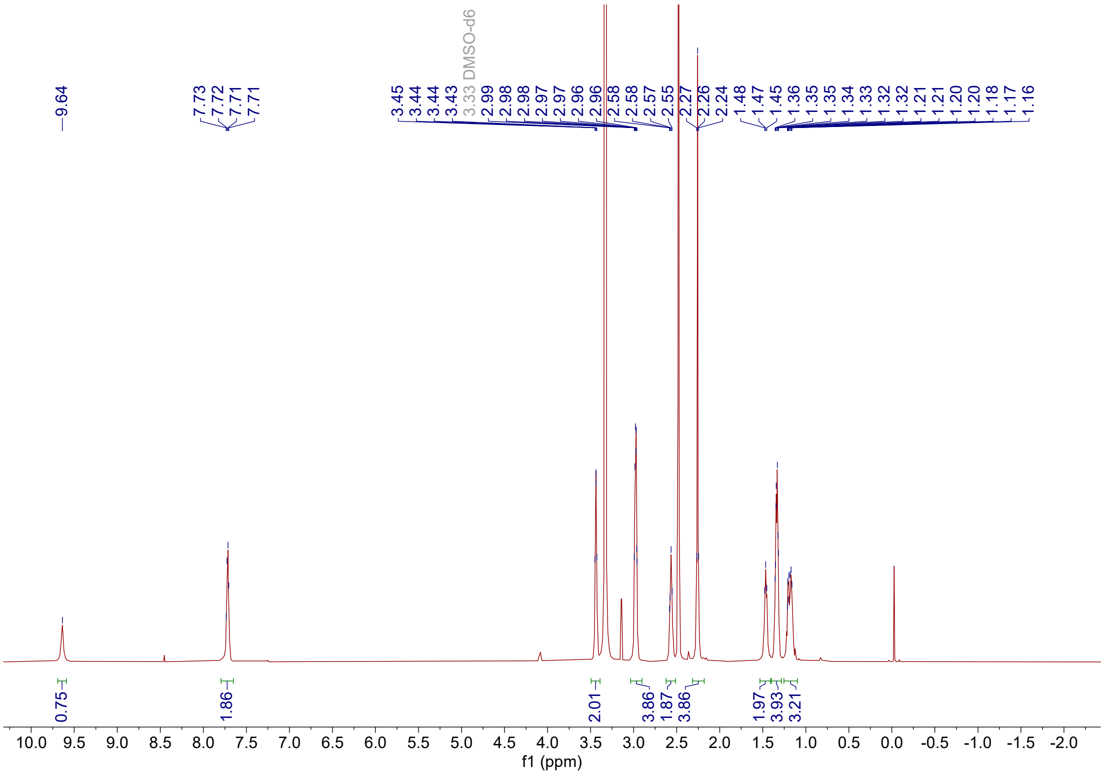

Figure S22a. ^{1}H NMR spectrum of **22** in DMSO.

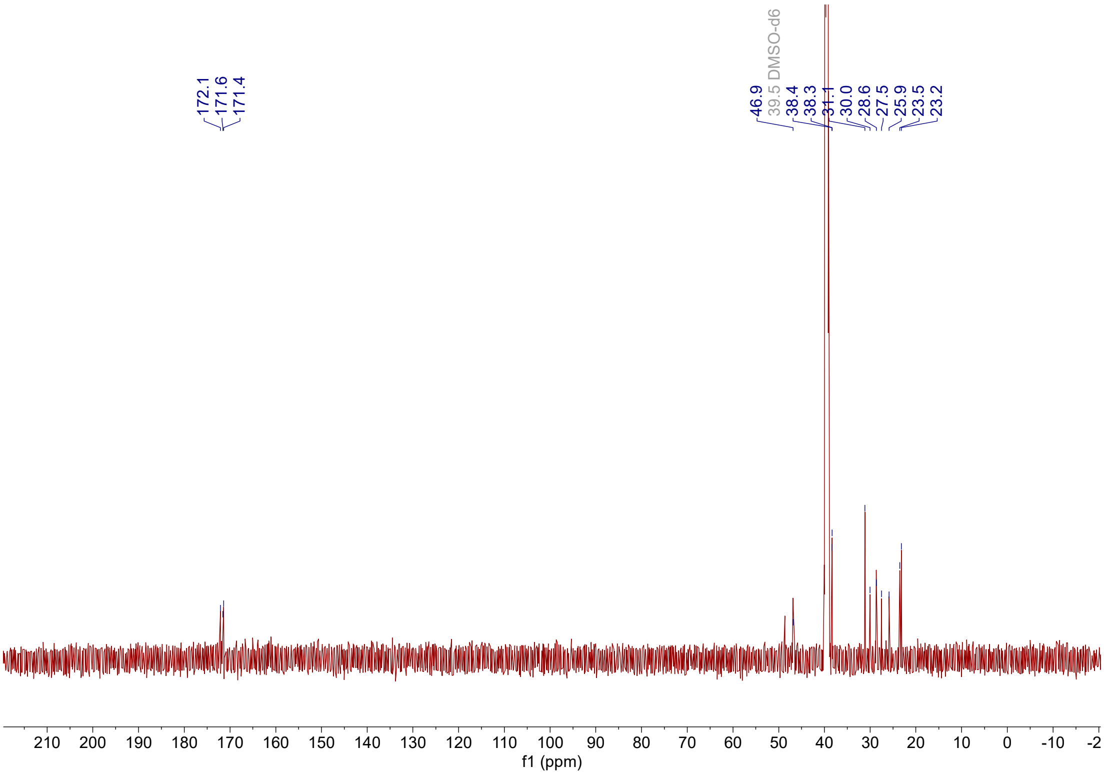

Figure S22b. ^{13}C NMR spectrum of **22** in DMSO.

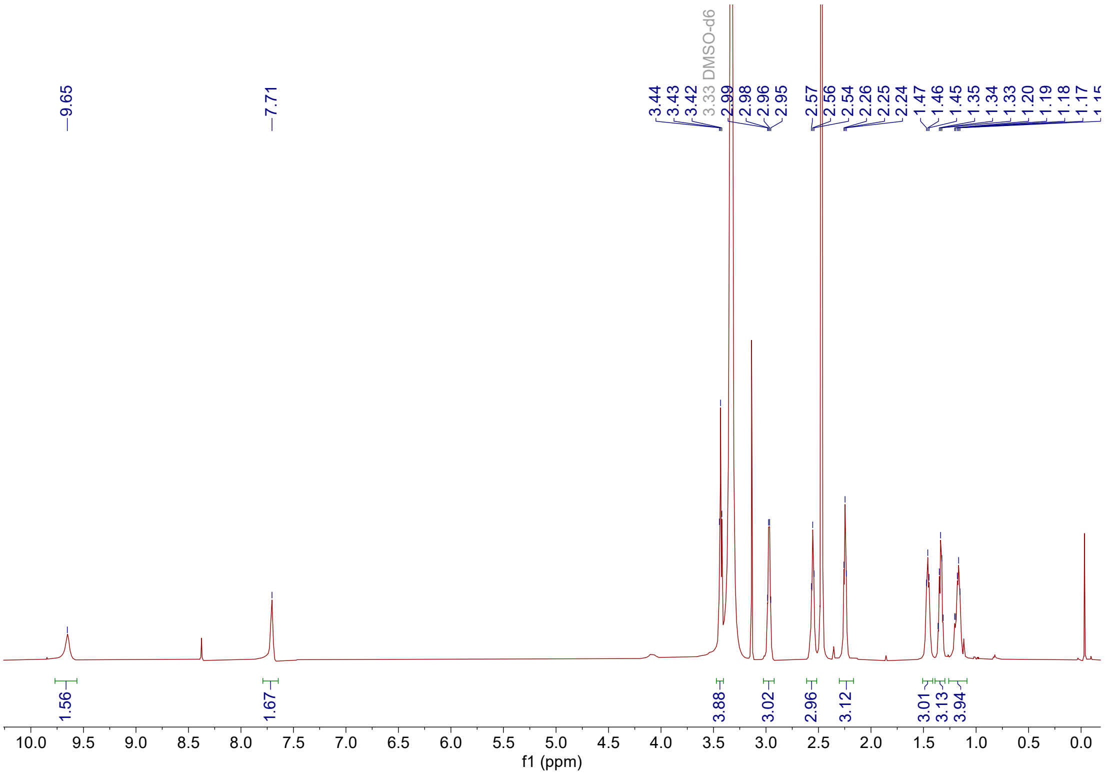

Figure S23a. ^{1}H NMR spectrum of **23** in DMSO.

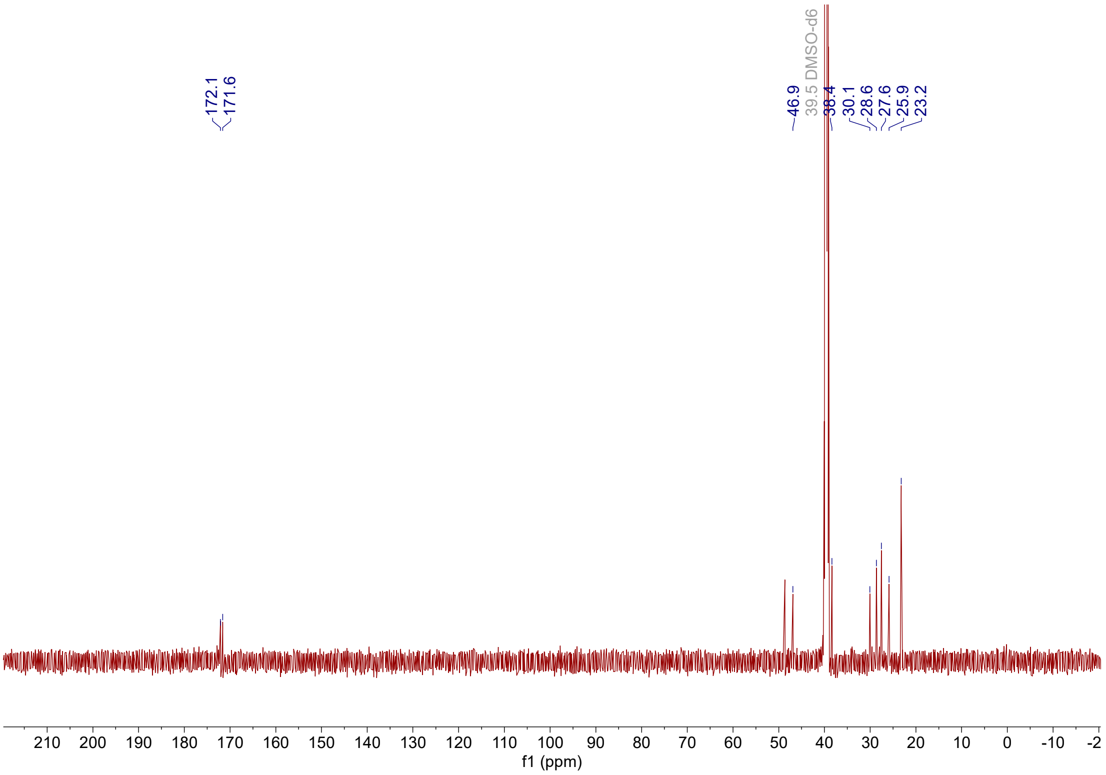

Figure S23b. ^{13}C NMR spectrum of **23** in DMSO.

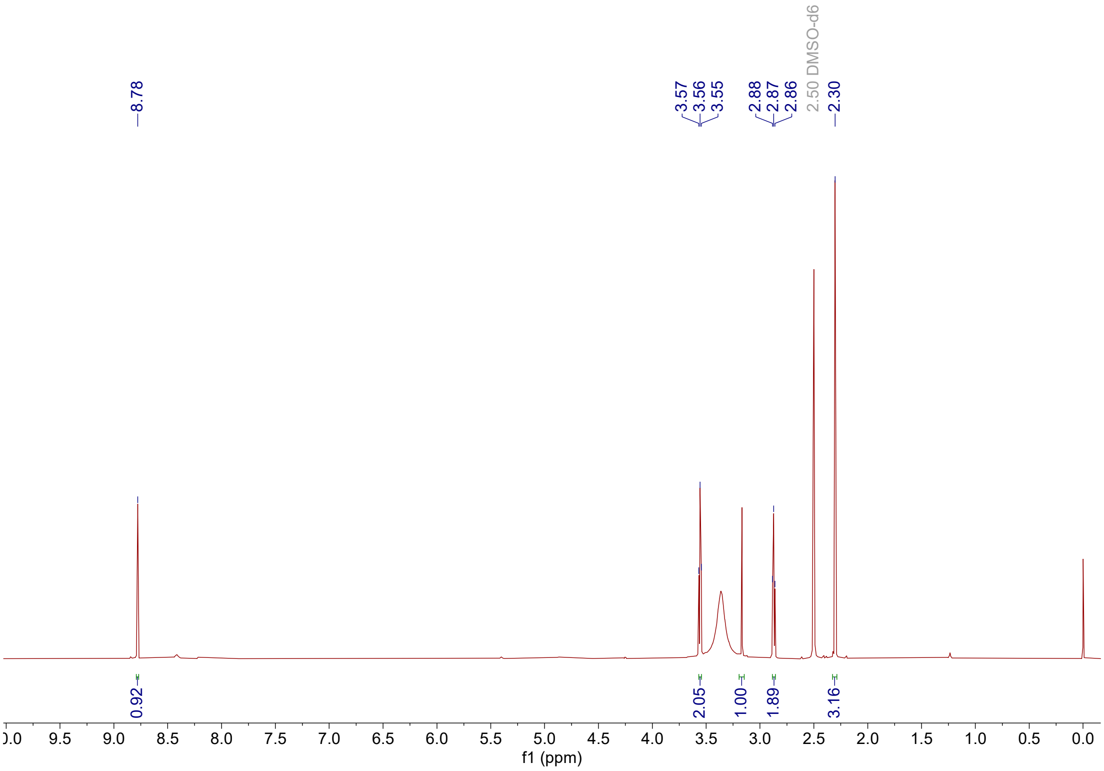

Figure S24a. ^{1}H NMR spectrum of **24** in DMSO.

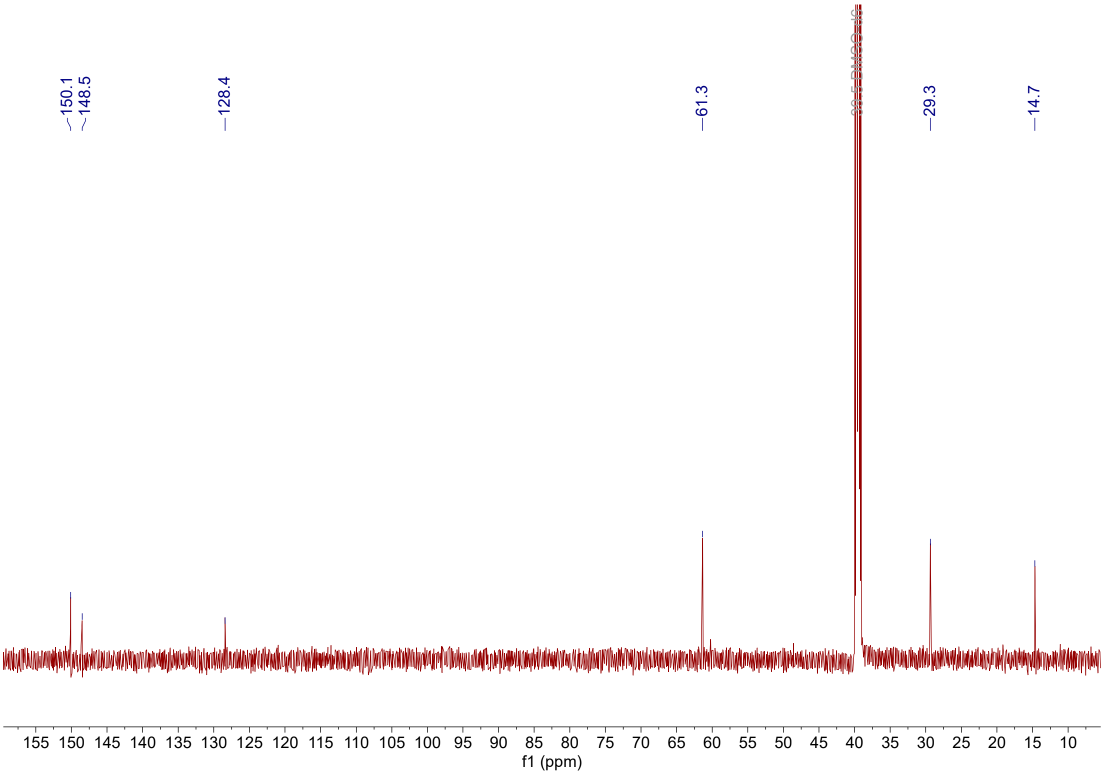

Figure S24b. ^{13}C NMR spectrum of **24** in DMSO.

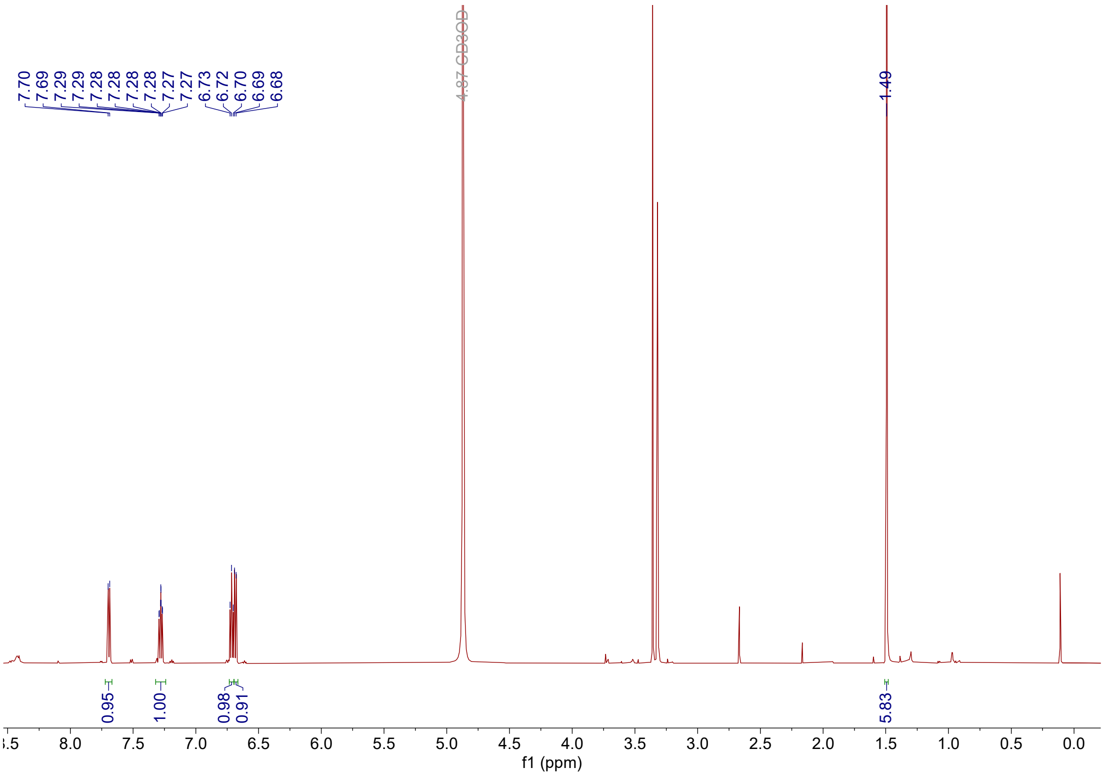

Figure S25a. ^{1}H NMR spectrum of **25** in CD_3OD.

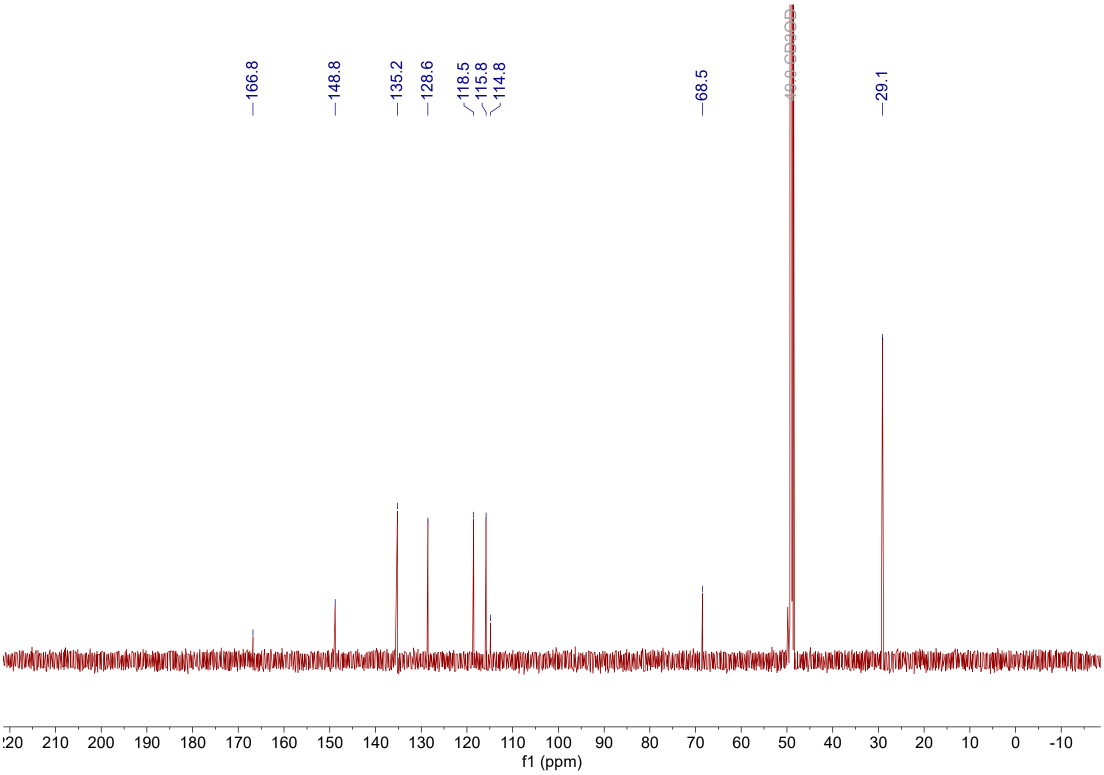

Figure S25b. ^{13}C NMR spectrum of **25** in CD_3OD.

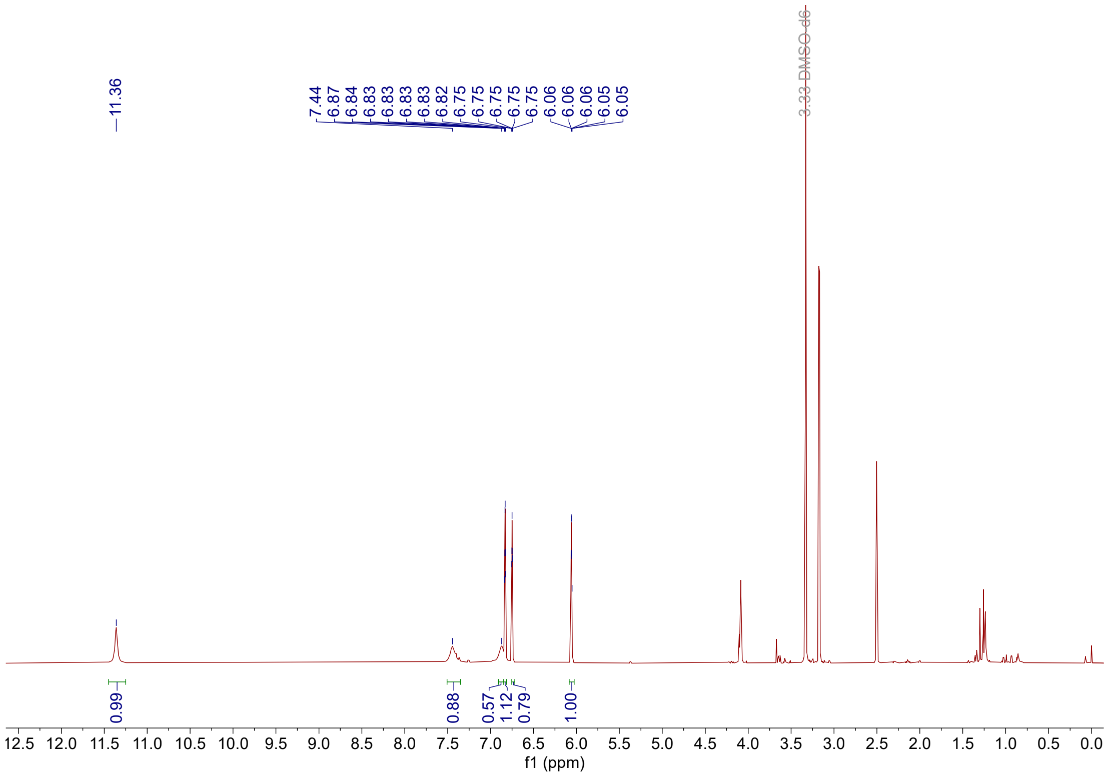

Figure S26a. ^{1}H NMR spectrum of **26** in DMSO.

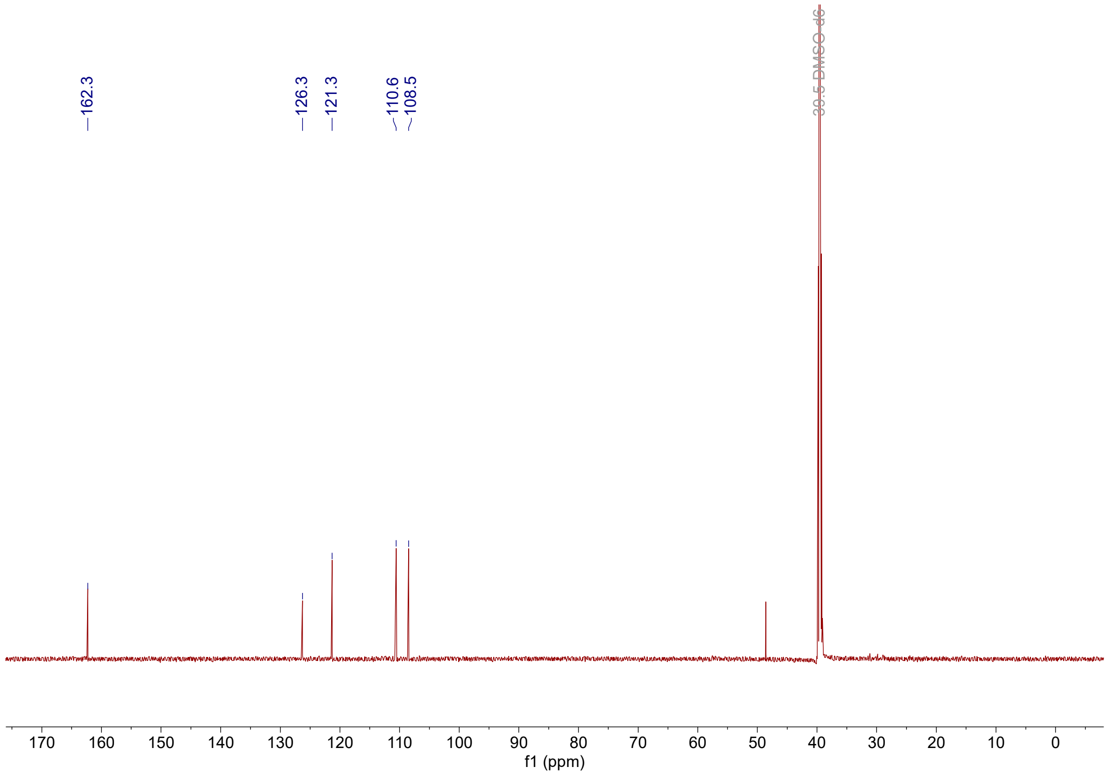

Figure S26b. ^{13}C NMR spectrum of **26** in DMSO.

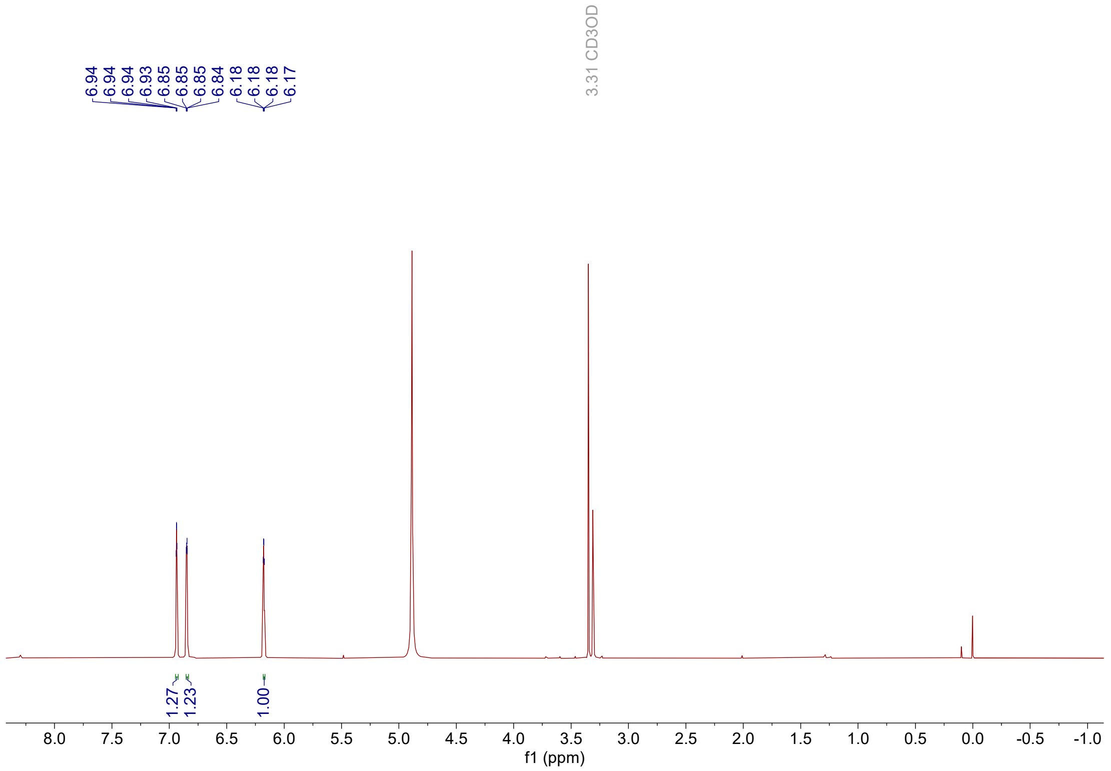

Figure S27a. ^{1}H NMR spectrum of **27** in CD_3OD.

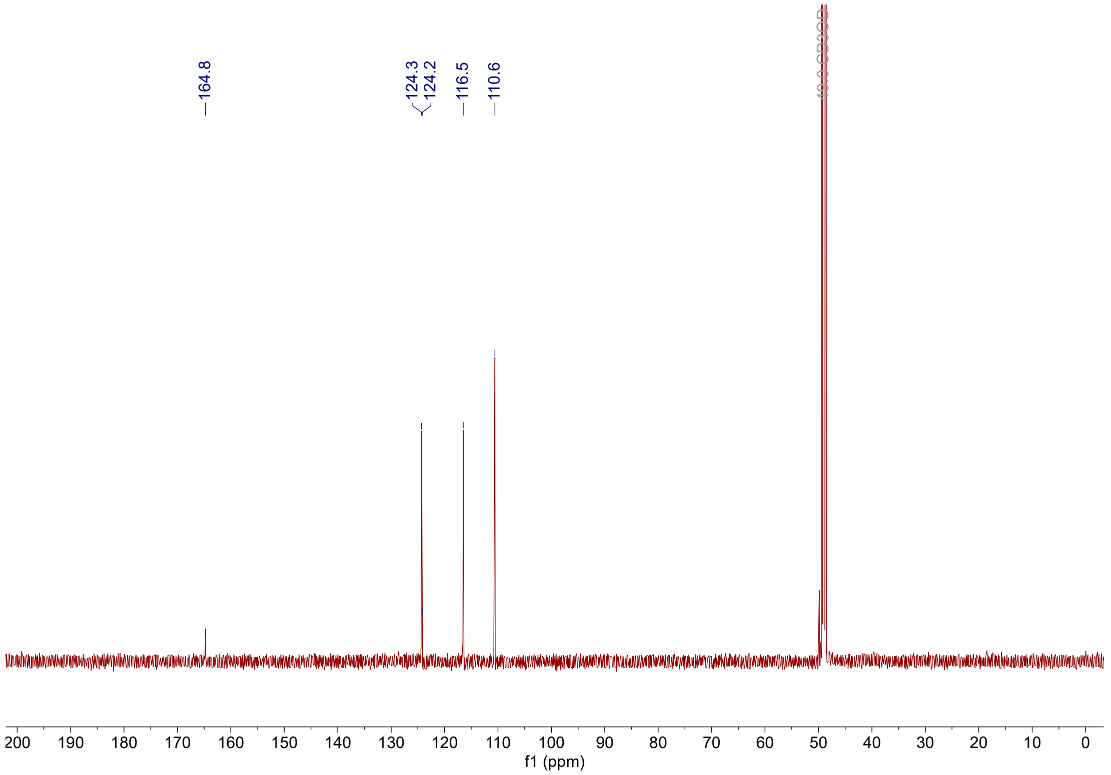

Figure S27b. ^{13}C NMR spectrum of **27** in CD_3OD.

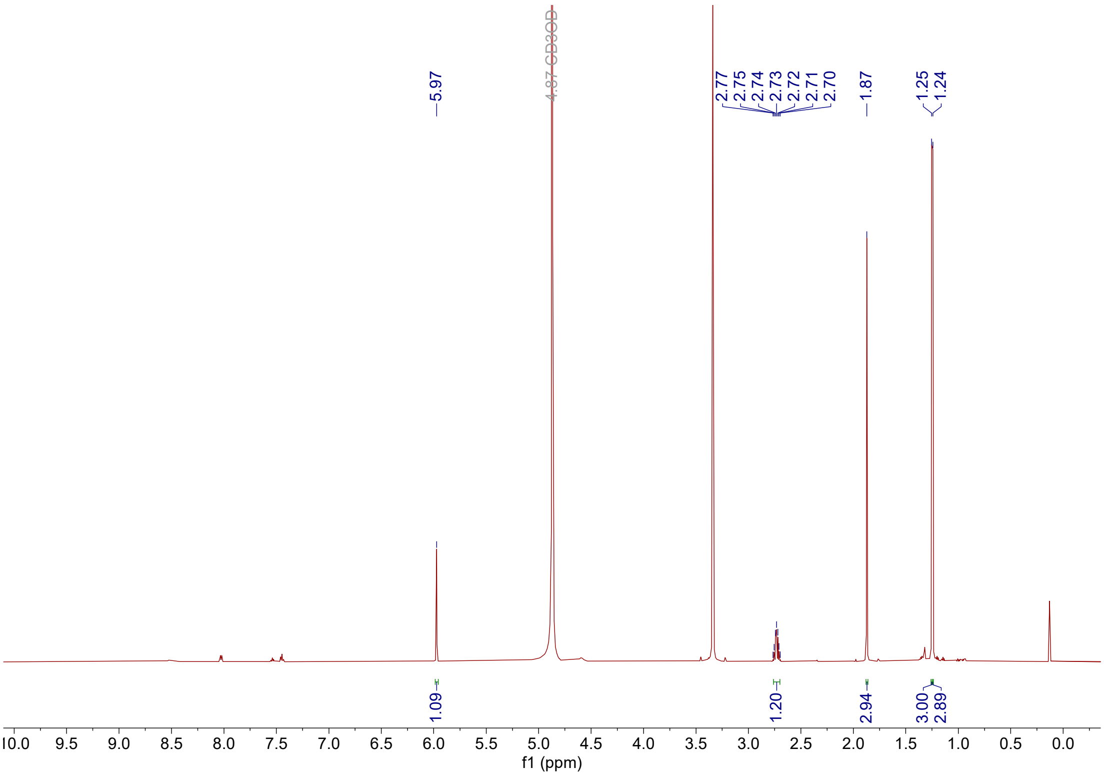

Figure S28a. ^{1}H NMR spectrum of **28** in CD_3OD.

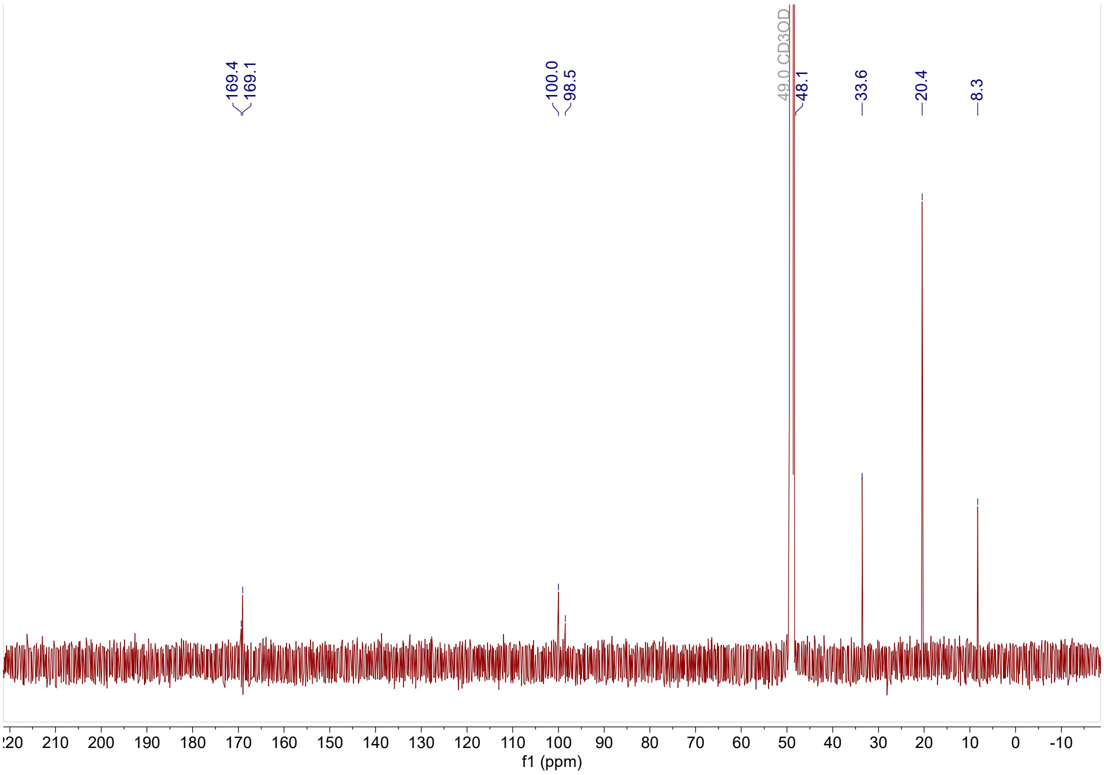

Figure S28b. ^{13}C NMR spectrum of **28** in CD_3OD.

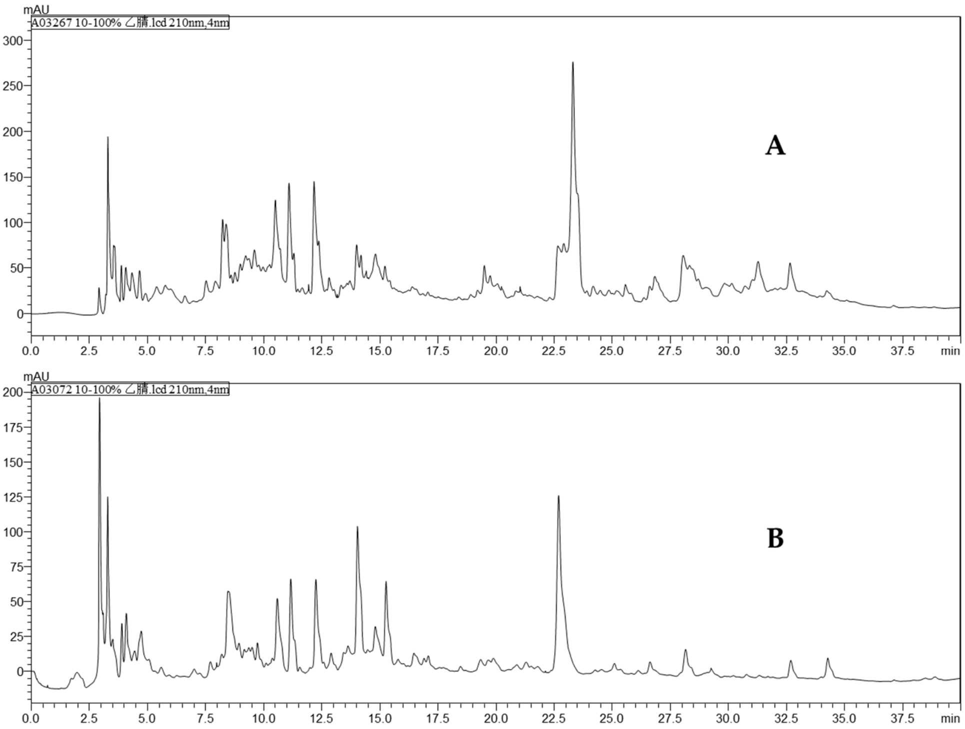

Figure S29. HPLC profiles of residues A and B.

NMR data for 3–28.

Autolytimycin (**3**): white amorphous solid. ^{1}H NMR (600 MHz, DMSO-d_6): δ 9.32 (s, 1H, HN-22), 6.60 (s, 1H, H-19), 6.25 (s, 1H, H-17), 6.20 (s, 1H, H-21), 5.68 (br s, 1H, H-3), 5.20 (d, *J* = 9.6 Hz, 1H, H-9), 4.84 (d, *J* = 7.2 Hz, 1H, H-7), 4.33 (m, 1H, H-11), 3.30 (s, 3H, CH_3O-6), 3.21 (s, 3H, CH_3O-12), 3.19 (m, 1H, H-6), 2.96 (d, *J* = 6.0 Hz, 1H, H-12), 2.29 (m, 1H, H-10), 2.54, 2.28 (m, 2H, H_2-15), 2.16, 2.03 (m, 2H, H_2-4), 1.79 (m, 1H, H-14), 1.70 (s, 3H, H_3-23), 1.36 (s, 3H, H_3-24), 1.23, 1.10 (m, 2H, H_2-5), 1.50, 1.08 (m, 2H, H_2-13), 0.91 (d, *J* = 6.6 Hz, 3H, H_3-25), 0.72 (d, *J* = 6.0 Hz, 3H, H_3-26). ^{13}C NMR (150 MHz, DMSO-d_6): δ 171.1 (C-1), 157.3 (H_2N-CO_2-7), 156.1 (C-18), 141.1 (C-16), 140.0 (C-20), 133.9 (C-3), 133.0 (C-9), 131.6 (C-2), 129.8 (C-8), 115.3 (C-21), 112.8 (C-17), 105.8 (C-19), 80.8 (C-7), 80.4 (C-12), 79.2 (C-6), 73.0 (C-11), 58.3 (CH_3O-6), 56.2 (CH_3O-12), 42.6 (C-15), 34.0 (C-10), 33.2 (C-13), 30.3 (C-14), 29.5 (C-5), 23.1 (C-4), 18.2 (C-26), 13.3 (C-23), 11.6 (C-24), 11.6 (C-25) [1].

Reblastatin (**4**): white solid. ^{1}H NMR (600 MHz, CD_3OD): 6.75 (s, 1H, H-19), 6.36 (s, 1H, H-21), 5.89 (br s, 1H, H-3), 5.32 (s, 1H, H-9), 4.97 (d, *J* = 7.8 Hz, 1H, H-7), 3.73 (s, 3H, CH_3O-17) , 3.57 (m, 1H, H-11), 3.47 (s, 3H, CH_3O-6), 3.37 (s, 3H, CH_3O-12), 3.34 (m, 1H, H-6), 3.14 (m, 1H, H-12), 2.76, 2.55 (m, 2H, H_2-15), 2.55 (m, 1H, H-10), 2.34, 2.19 (m, 2H, H_2-4), 1.96 (m, 1H, H-14), 1.80 (s, 3H, H_3-23), 1.74, 1.34 (m, 2H, H_2-13), 1.52 (s, 3H, H_3-24), 1.37, 1.15 (m, 2H, H_2-5), 1.04 (d, *J* = 6.6 Hz, 3H, H_3-25), 0.84 (d, *J* = 6.6 Hz, 3H, H_3-26). ^{13}C NMR (150 MHz, CD_3OD): δ 174.9 (C-1), 159.1 (H_2N-CO_2-7), 151.7 (C-20), 145.3 (C-17), 136.4 (C-3), 135.7 (C-2), 135.3 (C-16), 134.7 (C-9), 133.0 (C-18), 131.5 (C-8), 118.7 (C-21), 109.6 (C-19), 83.9 (C-7), 82.9 (C-12), 81.2 (C-6), 75.2 (C-11), 60.9 (CH_3O-17), 59.8 (CH_3O-6), 57.3 (CH_3O-12), 37.2 (C-15), 35.8 (C-10), 34.8 (C-13), 32.6 (C-14), 31.3 (C-5), 24.7 (C-4), 19.6 (C-26), 17.4 (C-25), 13.7 (C-23), 12.1 (C-24) [1].

17-*O*-Demethylgeldanamycin (**5**): yellow solid. ^{1}H NMR (600 MHz, $CDCl_3$): δ 8.95 (s, 1H, HN-22), 7.41 (s, 1H, H-19), 6.97 (d, *J* = 11.4 Hz, 1H, H-3), 6.57 (t, *J* = 11.4 Hz, 1H, H-4), 5.90 (t, *J* = 10.2 Hz, 1H, H-5), 5.79 (d, *J* = 9.6 Hz, 1H, H-9), 5.18 (s, 1H, H-7), 4.33 (d, *J* = 9.0 Hz, 1H, H-6), 3.55 (m, 1H, H-11), 3.35 (m, 1H, H-12), 3.36 (s, 3H, CH_3O-12), 3.31 (s, 3H, CH_3O-6), 2.80 (m, 1H, H-10), 2.44 (m, 2H, H_2-15), 2.03 (s, 3H, H_3-23), 1.80 (s, 3H, H_3-24), 1.77 (m, 2H, H_2-13), 1.74 (m, 1H, H-14), 1.00 (d, *J* = 6.6 Hz, 3H, H_3-25), 0.97 (d, *J* = 6.6 Hz, 3H, H_3-26). ^{13}C NMR (150 MHz, $CDCl_3$): δ 184.5 (C-21), 183.2 (C-18), 168.3 (C-1), 156.1 (H_2N-CO_2-7), 153.2 (C-17), 140.6 (C-20), 137.1

(C-5), 134.8 (C-2), 133.7 (C-8), 133.4 (C-9), 127.7 (C-3), 126.4 (C-4), 117.5 (C-16), 108.3 (C-19), 82.0 (C-7), 81.6 (C-6), 81.1 (C-12), 72.8 (C-11), 57.5 (CH_3O-6), 56.9 (CH_3O-12), 34.6 (C-13), 32.9 (C-15), 32.4 (C-10), 28.2 (C-14), 23.4 (C-26), 13.0 (C-24), 12.6 (C-23), 12.4 (C-25) [2].

Geldanamycin (**6**): yellow solid. ^{1}H NMR (600 MHz, $CDCl_3$): δ 8.71 (s, 1H, HN-22), 7.28 (s, 1H, H-19), 6.94 (d, *J* = 11.4 Hz, 1H, H-3), 6.56 (t, *J* = 11.4 Hz, 1H, H-4), 5.87 (t, *J* = 10.2 Hz, 1H, H-5), 5.82 (d, *J* = 10.2 Hz, 1H, H-9), 5.18 (s, 1H, H-7), 4.32 (d, *J* = 8.4 Hz, 1H, H-6), 4.12 (s, 3H, CH_3O-17), 3.54 (d, *J* = 7.0 Hz, 1H, H-11), 3.88–3.39 (m, 1H, H-12), 3.36 (s, 3H, CH_3O-12), 3.29 (s, 3H, CH_3O-6), 2.76–2.79 (m, 1H, H-10), 2.42–2.50 (m, 2H, H_2-15), 2.02 (s, 3H, H_3-23), 1.79 (s, 3H, H_3-24), 1.76–1.77 (m, 2H, H_2-13), 1.64–1.68 (m, 1H, H-14), 0.98 (d, *J* = 6.0 Hz, 3H, H_3-25), 0.97 (d, *J* = 6.0 Hz, 3H, H_3-26). ^{13}C NMR (150 MHz, $CDCl_3$): δ 185.1 (C-21), 184.3 (C-18), 168.3 (C-1), 157.1 (C-17), 156.1 (H_2N-CO_2-7), 138.2 (C-20), 136.6 (C-5), 135.0 (C-2), 133.4 (C-8), 133.3 (C-9), 127.7 (C-16), 127.4 (C-3), 126.5 (C-4), 111.9 (C-19), 81.7 (C-7), 81.4 (C-6), 81.2 (C-12), 72.9 (C-11), 61.8 (CH_3O-17), 57.4 (CH_3O-6), 56.8 (CH_3O-12), 34.8 (C-13), 32.9 (C-15), 32.3 (C-10), 28.1 (C-14), 23.1 (C-26), 13.0 (C-24), 12.7 (C-23), 12.5 (C-25) [2].

Cyclo(D-Trp-L-Tyr) (**7**): white solid. $[\alpha]_D^{20}$: −120.0 (*c* 0.1, MeOH). ^{1}H NMR (600 MHz, CD_3OD): δ 7.60 (d, *J* = 8.0 Hz, 1H, H-4), 7.35 (d, *J* = 8.1 Hz, 1H, H-7), 7.13 (t, *J* = 7.6 Hz, 1H, H-6), 7.06 (t, *J* = 7.5 Hz, 1H, H-5), 7.04 (s, 1H, H-2), 6.63 (d, *J* = 8.5 Hz, 2H, H-18, H-20), 6.47 (d, *J* = 8.5 Hz, 2H, H-17, H-21), 4.18 (m, 1H, H-9), 3.87 (m, 1H, H-12), 3.05 (dd, *J* = 14.6, 4.1 Hz, 1H, H-8a), 2.77 (dd, *J* = 14.6, 5.9 Hz, 1H, H-8b), 2.57 (dd, *J* = 13.7, 3.9 Hz, 1H, H-15a), 1.47 (dd, *J* = 13.7, 8.5 Hz, 1H, H-15b). ^{13}C NMR (150 MHz, CD_3OD): δ 170.2 (C-11), 169.5 (C-14), 157.6 (C-19), 138.1 (C-7a), 132.0 (C-17,C-21), 128.9 (C-3a), 127.8 (C-16), 125.9 (C-2), 122.7 (C-6), 120.2 (C-5), 119.9 (C-4), 116.3 (C-18, C-20), 112.5 (C-7), 109.6 (C-3), 57.9 (C-12), 57.1 (C-9), 40.1 (C-15), 31.2 (C-8) [3].

Cyclo(L-Pro-L-Trp) (**8**): white solid. $[\alpha]_D^{20}$: −55.6 (*c* 0.09, MeOH). ^{1}H NMR (600 MHz, $CDCl_3$): δ 8.20 (s, 1H, H-1), 7.59 (d, *J* = 7.9 Hz, 1H, H-4), 7.40 (d, *J* = 8.2 Hz, 1H, H-7), 7.24 (t, *J* = 7.5 Hz, 1H, H-6), 7.15 (t, *J* = 7.5 Hz, 1H, H-5), 7.11 (s, 1H, H-2), 5.73 (s, 1H, H-10), 4.38 (dd, *J* = 11.0, 3.7 Hz, 1H, H-9), 4.08 (t, *J* = 8.1 Hz, 1H, H-12), 3.77 (dd, *J* = 15.1, 3.8 Hz, 1H, H-8a), 3.66 (m, 1H, H-15a), 3.58 (m, 1H, H-15b), 2.97 (dd, *J* = 15.1, 10.9 Hz, 1H, H-8b), 2.33 (m, 1H, H-13a), 2.01 (m, 2H, H-13b, H-14a), 1.91 (m, 1H, H-14b). ^{13}C NMR (150 MHz, $CDCl_3$): δ 169.5 (C-11), 165.7 (C-17), 136.8 (C-7a), 126.9 (C-3a), 123.4 (C-2), 123.0 (C-6), 120.2 (C-5), 118.7 (C-4), 111.7 (C-7), 110.2 (C-3), 59.4 (C-9), 54.7 (C-12), 45.6 (C-15), 28.5 (C-13), 27.0 (C-8), 22.8 (C-14) [4].

Cyclo(L-Ser-L-Trp) (**9**): yellow oil. $[\alpha]_D^{20}$: −38.0 (*c* 0.1, MeOH), ^{1}H NMR (600 MHz, CD_3OD): δ 7.65 (d, *J* = 7.8 Hz, 1H, H-4), 7.37 (d, *J* = 8.4 Hz, 1H, H-7), 7.14 (t, *J* = 6.6 Hz, 1H, H-6), 7.14 (s, 1H, H-2), 7.04 (t, *J* = 7.8 Hz, 1H, H-5), 4.27 (s, 1H, H-9), 3.85 (d, *J* = 5.4 Hz, 1H, H-12), 3.41 (d, *J* = 11.4 Hz, 2H, H-15), 3.41 (dd, *J* = 11.4, 3.6 Hz, 1H, H-8a), 2.94 (d, *J* = 5.4 Hz, 1H, H-8b). ^{13}C NMR (150 MHz, CD_3OD): δ 170.2 (C-14), 168.1 (C-11), 138.0 (C-7a), 128.9 (C-3a), 125.4 (C-2), 122.5 (C-6), 120.1 (C-4), 119.7 (C-5), 112.3 (C-7), 109.9 (C-3), 64.7 (C-15), 58.8 (C-9), 57.5 (C-12), 31.9 (C-8) [5].

Cyclo(L-Tyr-L-Pro) (**10**): white solid. $[\alpha]_D^{20}$: −72.0 (*c* 0.1, MeOH). ^{1}H NMR (600 MHz, CD_3OD): δ 7.07 (d, *J* = 8.5 Hz, 2H, H-2′, H-6′), 6.74 (d, *J* = 8.5 Hz, 2H, H-3′, H-5′)), 4.40 (m, 1H, H-9), 4.08 (m, 1H, H-6), 3.59 (m, 1H, H-3), 3.39 (m, 1H, H-3), 3.10 (m, 2H, H_2-10), 2.13 (m, 1H, H-5), 1.84 (m, 2H, H_2-4), 1.27 (m, 1H, H-5). ^{13}C NMR (150 MHz, CD_3OD): δ 170.8 (C-7), 167.0 (C-1), 157.7 (C-4′), 132.1 (C-2′, C-6′), 127.7 (C-1′), 116.2 (C-3′, C-5′), 60.1 (C-6), 57.9 (C-9), 45.9 (C-3), 37.7 (C-10), 29.4 (C-5), 22.7 (C-4) [6].

Cyclo(L-Ile-L-Tyr) (**11**): white solid. $[\alpha]_D^{20}$: −24.0 (*c* 0.1, MeOH). ^{1}H NMR (600 MHz, CD_3OD): δ 7.02 (d, *J* = 8.5 Hz, 2H, H-13, H-17), 6.69 (d, *J* = 8.5 Hz, 2H, H-14, H-16), 4.26 (m, 1H, H-2), 3.70 (dd, *J* = 4.2, 1.7 Hz, 1H, H-5), 3.18 (m, 1H, H-11a), 2.89 (dd, *J* = 14.0, 4.7 Hz, 1H, H-11b), 1.42 (m, 1H, H-7), 0.80 (m, 1H, H-8), 0.76 (d, *J* = 7.1 Hz, 3H, H_3-10), 0.71 (t, *J* = 7.1 Hz, 3H, H_3-9). ^{13}C NMR (150 MHz, CD_3OD): δ 169.9 (C-1, C-4), 157.9 (C-15), 132.6 (C-13, C-17), 127.5 (C-12), 116.3 (C-14, C-16), 60.9 (C-2), 57.4 (C-5), 39.8 (C-7), 38.9 (C-11), 24.7 (C-8), 15.4 (C-10), 11.9 (C-9) [7].

Cyclo(L-Ile-L-Phe) (**12**): white solid. $[\alpha]_D^{20}$: −8.0 (*c* 0.1, MeOH). ^{1}H NMR (600 MHz, DMSO-d_6): δ 8.10 (s, 1H, HN-6), 7.88 (s, 1H, HN-3), 7.21 (m, 5H, H-13, H-14, H-15, H-16, H-17), 4.21 (m, 1H, H-5), 3.58 (m, 1H, H-2), 3.15 (m, 1H, H-11a), 2.86 (dd, *J* = 13.5, 5.1 Hz, 1H, H-11b), 1.40 (m, 1H, H-7), 0.65 (m, 2H, H_2-8), 0.57 (m, 3H, H_3-9), 0.56 (d, *J* = 7.1 Hz, 3H, H_3-10). ^{13}C NMR (150 MHz, DMSO-d_6): δ 166.4 (C-1, C-4), 136.3 (C-12), 130.4 (C-13, C-17), 127.9 (C-14, C-16), 126.5 (C-15), 58.7 (C-2), 55.0 (C-5), 37.8 (C-11), 37.6 (C-7), 23.2 (C-8), 14.4 (C-10), 11.7 (C-9) [8].

Cyclo(4-OH-L-Pro-L-Phe) (**13**): white solid. $[\alpha]_D^{20}$: −84.0 (*c* 0.1, MeOH), ^{1}H NMR (600 MHz, CD_3OD): δ 7.20–7.29 (m, 5H, H-2′, H-3′, H-4′, H-5′, H-6′), 4.59 (br s, 1H, H-8), 4.51 (td, *J* = 4.8, 1.2 Hz, 1H, H-9), 4.38 (dd, *J* = 6.0, 1.2 Hz, 1H, H-6), 4.28 (t, *J* = 4.8 Hz, 1H, H-4), 3.74 (dd, *J* = 12.6, 4.8 Hz, 1H, H-3b), 3.29 (s, 1H, H-3a), 3.17 (t, *J* = 6.0 Hz, 1H, H-10a), 3.17 (t, *J* = 6.0 Hz, 1H, H-10b), 2.09 (q, *J* = 6.0 Hz, 1H, H-5a), 1.41 (m, 1H, H-5b). ^{13}C NMR (150 MHz, CD_3OD): δ 171.3 (C-7), 167.1 (C-1), 137.4 (C-1′), 131.0 (C-2′), 129.5 (C-3′), 128.0 (C-4′), 68.5 (C-4), 58.3 (C-6), 57.6 (C-9), 55.2 (C-3), 38.9 (C-5), 38.0 (C-10) [9].

Cyclo(L-Ser-L-Phe) (**14**): white solid. $[\alpha]_D^{20}$: −3.3 (*c* 0.06, MeOH). ^{1}H NMR (600 MHz, DMSO-*d*6): δ 7.99 (d, *J* = 2.6 Hz, 1H, HN-3), 7.89 (d, *J* = 2.5 Hz, 1H, HN-6), 7.28 (t, *J* = 7.4 Hz, 2H, H-3′, H-5′), 7.22 (dd, *J* = 8.5, 1.6 Hz,1H, H-4′), 7.17 (t, *J* =7.3 Hz, 2H, H-2′, H-6′), 4.87 (s, 1H, OH), 4.05 (m, 1H, H-2), 3.66 (m, 1H, H-5), 3.28 (m, 1H, H-7a), 3.10 (dd, *J* = 13.5, 6.1 Hz, 1H, H-8a), 2.98 (dd, *J* = 13.5, 5.0 Hz, 1H, H-8b), 2.85 (dd, *J* = 10.8, 5.8 Hz, 1H, H-7b). ^{13}C NMR (150 MHz, DMSO-*d*6): δ 167.5 (C-1), 165.8 (C-4), 136.7 (C-1′), 129.9 (C-6′, C-2′), 128.1 (C-5′, C-3′), 126.5 (C-4′), 63.1 (C-7), 57.1 (C-5), 55.5 (C-2), 40.1 (C-8) [10].

Cyclo(L-Phe-L-Ala) (**15**): white solid. $[\alpha]_D^{20}$: −10.0 (*c* 0.1, MeOH), ^{1}H NMR (600 MHz, CD_3OD): δ 7.20–7.29 (m, 5H, H-2′, H-3′, H-4′, H-5′, H-6′), 4.32 (td, *J* = 4.8, 1.2 Hz, 1H, H-2), 3.78 (m, 1H, H-5), 3.28 (dd, *J* = 13.8, 4.2 Hz, 1H, H-8b), 2.98 (dd, *J* = 13.8, 4.8 Hz, 1H, H-8a), 0.54 (d, *J* = 6.6 Hz, 3H, H_3-7). ^{13}C NMR (150 MHz, CD_3OD): δ 170.6 (C-4), 168.5 (C-1), 136.6 (C-1′), 131.8 (C-2′), 129.5 (C-3′), 128.3 (C-4′), 57.4 (C-2), 51.5 (C-5), 40.3 (C-8), 20.4 (C-7) [8].

Cyclo(L-Leu-L-Ile) (**16**): white solid. $[\alpha]_D^{20}$: −16.0 (*c* 0.1, MeOH), ^{1}H NMR (600 MHz, $CDCl_3$): δ 4.02 (m, 1H, H-2), 3.95 (dd, *J* = 1.28, 3.68 Hz, 1H, H-5), 2.13 (m, 1H, H-12), 1.91 (m, 1H, H-7), 1.78 (m, 1H, H-11a), 1.62 (m, 1H, H-11b), 1.47 (m, 1H, H-8a), 1.21 (m, 1H, H-8b), 1.04 (d, *J* = 7.2 Hz, 3H, H_3-10), 1.01 (d, *J* = 6.6 Hz, 3H, H_3-13), 0.95 (d, *J* = 6.6 Hz, 3H, H_3-14), 0.88 (t, *J* = 6.6 Hz, 3H, H_3-9). ^{13}C NMR (150 MHz, $CDCl_3$): δ 168.5 (C-1), 167.1 (C-4), 60.2 (C-5), 53.2 (C-2), 43.5 (C-11), 38.3 (C-7), 24.5 (C-12), 24.1 (C-8), 23.5 (C-13), 21.1 (C-14), 15.6 (C-10), 11.9 (C-9) [11].

Cyclo(L-Ala-L-Pro) (**17**): white solid. $[\alpha]_D^{20}$:−98.0 (*c* 0.1, MeOH). ^{1}H NMR (600 MHz, DMSO-d6): δ 8.13 (s, 1H, NH), 4.16 (m, 1H, H-2), 4.08 (m, 1H, H-2′), 3.36 (m, 2H, H-5), 2.12 (m, 1H, H-3a), 1.84 (m, 1H, H-3b), 1.84 (m, 2H, H_2-4), 1.21 (d, *J* = 6.8 Hz, 3H, H_3-3′). ^{13}C NMR (150 MHz, DMSO-d6): δ 169.9 (C-1), 166.5 (C-1′), 58.6 (C-2), 50.1 (C-5), 44.8 (C-2′), 27.6 (C-3), 22.3 (C-4), 15.2 (C-3′) [12].

Cyclo(L-Pro-L-Val) (**18**): white solid. $[\alpha]_D^{20}$: −86.0 (*c* 0.1, MeOH). ^{1}H NMR (600 MHz, CD_3OD): δ 4.22 (m, 1H, H-2′), 4.05 (t, 1H, *J* = 2.1 Hz, H-2), 3.54 (m, 2H, H-5), 2.50 (m, 1H, H-3′), 2.33 (m, 1H, H-3a), 2.03 (m, 1H, H-3b), 1.96 (m, 2H, H_2-4), 1.10 (d, *J* = 7.2 Hz, 3H, H_3-4′), 0.95 (d, *J* = 6.9 Hz, 3H, H_3-5′). ^{13}C NMR (150 MHz, DMSO-d6): δ 172.6 (C-1), 167.6 (C-1′), 61.5 (C-2), 60.0 (C-2′), 45.3 (C-5), 29.9 (C-3), 29.5 (C-3′), 23.3 (C-4), 18.8 (C-4′), 16.7 (C-5′) [13].

6-(2,3-Dihydroxy-3-methyibutyl)indolin-2-one (**19**): yellow oil. $[\alpha]_D^{20}$: +6.0 (*c* 0.1, MeOH), ^{1}H NMR (600 MHz, DMSO-*d*6): δ 10.31 (s, 1H, H-1), 7.07 (d, *J* = 7.8 Hz, 1H, H-4), 6.78 (d, *J* = 9.0 Hz, 1H, H-5), 6.72 (s, 1H, H-7), 4.41 (d, *J* = 6.0 Hz, 1H, HO-9), 4.24 (s, 1H, HO-10), 3.40 (s, 1H, H-3), 3.27 (dd, *J* = 8.4, 4.8 Hz, 1H, H-9), 2.89 (d, *J* = 13.8 Hz, 1H, H-8a), 2.32 (dd, *J* = 13.8, 10.2 Hz, 1H, H-8b), 1.11 (s, 3H, H_3-11), 1.06 (s, 3H, H_3-12). ^{13}C NMR (150 MHz, DMSO-*d*6): δ 176.7 (C-2), 143.5 (C-7a), 140.9 (C-6), 123.8 (C-4), 122.7 (C-3a), 122.1 (C-5), 110.2 (C-7), 79.2 (C-9), 71.7 (C-10), 37.6 (C-8), 35.5 (C-3), 26.9 (C-11), 24.1 (C-12) [14].

17-Hydroxycyclooctatin (**20**): yellow oil. ^{1}H NMR (600 MHz, CD_3OD): δ 5.39 (m, 1H, H-9), 4.45 (m, 1H, H-5), 3.68 (dd, *J* = 10.2, 7.8 Hz, 1H, H-18), 3.62 (dd, *J* = 10.8, 3.6 Hz, 1H, H-17), 3.56 (dd, *J* = 10.8, 6.6 Hz, 1H, H-18), 3.24 (dd, *J* = 10.2, 8.4 Hz, 1H, H-17), 2.76, 1.94 (m, 2H, H-8), 2.64 (m, 1H, H-3), 2.61 (m, 1H, H-2), 2.39 (m, 1H, H-11), 1.99 (m, 1H, H-6), 1.81 (m, 1H, H-15), 1.72, 1.39 (m, 2H, H_2-4), 1.62, 1.45 (m, 2H, H_2-1), 1.69, 1.23 (m, 2H, H_2-13), 1.67, 1.34 (m, 2H, H_2-12), 1.36 (s, 3H, H_3-19), 1.28 (s, 3H, H_3-20), 1.04 (d, *J* = 6.6 Hz, 3H, H_3-16). ^{13}C NMR (150 MHz, CD_3OD): δ 153.9 (C-10), 119.5 (C-9), 78.3 (C-7), 75.6 (C-5), 65.6 (C-17), 63.4 (C-18), 57.9 (C-6), 52.3 (C-11), 46.6 (C-1), 45.8 (C-14), 45.5 (C-13), 44.9 (C-3), 42.2 (C-8), 39.7 (C-4), 38.7 (C-15), 35.8 (C-2), 26.7 (C-19), 25.9 (C-12), 25.3 (C-20), 17.3 (C-16) [15].

16,17-Dihydroxycyclooctatin (**21**): yellow oil. ^{1}H NMR (600 MHz, CD_3OD): δ 5.47 (t, *J* = 8.4 Hz, 1H, H-9), 4.49 (t, *J* = 4.8 Hz, 1H, H-5), 3.86 (q, *J* = 5.4 Hz, 1H, H-16), 3.69 (m, 1H, H-16), 3.63 (m, 1H, H-17), 3.61 (m, 2H, H-18), 3.42 (dd, *J* = 10.8, 8.4 Hz, 1H, H-17), 2.80 (t, *J* = 11.4 Hz, 1H, H-8), 2.60–2.66 (m, 3H, H-2, H-3, H-11), 1.95–2.00 (m, 2H, H-6, H-8), 1.93 (m, 1H, H-13), 1.75 (m, 2H, H-4, H-12), 1.67 (m, 1H, H-13), 1.63 (m, 1H, H-1), 1.49 (m, 1H, H-1), 1.42 (m, 1H, H-12), 1.38 (s, 3H, H_3-19), 1.31 (s, 3H, H_3-20), 1.31 (m, 1H, H-4),

1.23 (m, 1H, H-15). ^{13}C NMR (150 MHz, CD_3OD): δ 153.9 (C-10), 118.7 (C-9), 78.3 (C-7), 75.6 (C-5), 63.9 (C-16), 63.4 (C-18), 61.5 (C-17), 58.0 (C-6), 47.2 (C-11), 46.6 (C-1), 45.8 (C-14), 45.7 (C-15), 45.6 (C-13), 44.9 (C-3), 42.2 (C-8), 39.7 (C-4), 35.8 (C-2), 26.7 (C-19), 25.5 (C-20), 25.3 (C-12) [16].

Terragine E (**22**): white solid. ^{1}H NMR (600 MHz, DMSO-d_6): δ 9.64 (br s, 2H, OH), 7.72 (s, 4H, HN), 3.44 (t, *J* = 6.6 Hz, 2H, H-18, H-29), 2.97 (dt, *J* = 7.0, 6.0 Hz, 4H, H-7, H-11, H-22, H-33), 2.57 (t, *J* = 9.6 Hz, 2H, H-15, H-26), 2.26 (t, *J* = 6.6 Hz, 2H, H-14, H-25), 2.26 (s, 2H, H-3, H-4), 1.47 (m, 2H, H-19, H-30), 1.34 (m, 4H, H-8, H-10, H-21, H-32), 1.20 (m, 3H, H-9, H-20, H-31). ^{13}C NMR (150 MHz, DMSO-d_6): δ 172.0 (C-16, C-27), 171.5 (C-5, C-13), 171.4 (C-2, C-24), 46.8 (C-18, C-29), 38.3 (C-7, C-11, C-22, C-33), 31.1 (C-3, C-4), 30.0 (C-14, C-25), 28.6 (C-8, C-10, C-21, C-32), 27.5 (C-15, C-26), 25.8 (C-19, C-30), 23.5 (C-9), 23.2 (C-20, C-31) [17].

Deferriferrioxamine E (**23**): white solid. ^{1}H NMR (600 MHz, DMSO-d_6): δ 9.65 (s, 3H, HO), 7.71 (t, *J* = 6.0 Hz, 3H, HN), 3.43 (t, *J* = 7.2 Hz, 6H, H-7, H-18, H-29), 2.96 (dt, *J* = 7.2, 6.0 Hz, 6H, H-11, H-22, H-33), 2.56 (t, *J* = 6.0 Hz, 6H, H-4, H-15, H-26), 2.25 (t, *J* = 7.2 Hz, 6H, H-3, H-14, H-25), 1.46 (m, 6H, H-8, H-19, H-30), 1.34 (m, 6H, H-10, H-21, H-32), 1.19 (m, 6H, H-9, H-20, H-31). ^{13}C NMR (150 MHz, DMSO-d_6): δ 172.1 (C-5, C-16, C-27), 171.6 (C-2, C-13, C-24), 46.9 (C-7, C-18, C-29), 38.4 (C-11, C-22, C-33), 30.1 (C-3, C-14, C-25), 28.6 (C-10, C-21, C-32), 27.5 (C-4, C-15, C-26), 25.9 (C-8, C-19, C-30), 23.2 (C-9, C-20, C-31) [18].

4-(2-Hydroxyethyl)-5-methyloxazole (**24**): white solid. ^{1}H NMR (600 MHz, DMSO-d_6): δ 8.78 (s, 1H, H-2), 3.56 (t, *J* = 6.5 Hz, 2H, H_2-8), 2.87 (t, *J* = 6.5 Hz, 2H, H_2-7), 2.30 (s, 3H, H_3-6). ^{13}C NMR (150 MHz, DMSO-d_6): δ 150.1 (C-2), 148.5 (C-5), 128.4 (C-4), 61.3 (C-8), 29.3 (C-7), 14.7 (C-6) [19].

2,3-Dihydro-2,2-dimethyl-4 (1*H*)-quinazolinone (**25**): white solid. ^{1}H NMR (600 MHz, CD_3OD): δ 7.70 (d, *J* = 7.8 Hz, 1H, H-5), 7.29 (t, *J* = 8.8 Hz, 1H, H-7), 6.72 (t, *J* = 8.8 Hz, 1H, H-8), 6.69 (d, *J* = 8.2 Hz, 1H, H-6), 1.50 (s, 6H, H_3-9, H_3-10). ^{13}C NMR (150 MHz, CD_3OD): δ 166.8 (C-4), 148.8 (C-8a), 135.2 (C-7), 128.6 (C-5), 118.5 (C-8), 115.8 (C-6), 114.8 (C-5a), 68.5 (C-2), 29.1 (C-9, C-10) [20].

1*H*-Pyrrole-2-carboxamide (**26**): white solid. ^{1}H NMR (600 MHz, DMSO-d_6): δ 11.36 (br s, 1H, H-1), 7.44 (br s, 2H, H_2-7), 6.83 (m, 1H, H-5), 6.75 (m, 1H, H-3), 6.06 (m, 1H, H-4). ^{13}C NMR (150 MHz, DMSO-d_6): δ 162.3 (C-6), 126.3 (C-2), 121.3 (C-5), 110.6 (C-3), 108.5 (C-4) [21].

1*H*-Pyrrole-2-carboxylic acid (**27**): white solid. ^{1}H NMR (600 MHz, CD_3OD): δ 6.94 (dd, *J* = 2.5, 1.5 Hz, 1H, H-5), 6.85 (dd, *J* = 3.7, 1.5 Hz, 1H, H-3), 6.18 (dd, *J* = 3.7, 2.5 Hz, 1H, H-4). ^{13}C NMR (150 MHz, CD_3OD): δ 164.8 (COOH), 124.3 (C-2), 124.2 (C-5), 116.5 (C-3), 110.6 (C-4) [22].

Surugapyrone A (**28**): white solid. ^{1}H NMR (600 MHz, CD_3OD): δ 5.97 (s, 1H, H-5), 2.72 (m, 1H, H-7), 1.87 (s, 3H, H_3-10), 1.25 (d, *J* = 6.6 Hz, 6H, H_3-8, H_3-9). ^{13}C NMR (150 MHz, CD_3OD): δ 169.4 (C-6), 169.0 (C-2), 167.9 (C-4), 100.0 (C-3), 98.5 (C-5), 33.5 (C-7), 20.4 (C-8, C-9), 8.8 (C-10) [23].

References

1. Wrona, I.E.; Gozman, A.; Taldone, T.; Chiosis, G.; Panek, J.S. Synthesis of reblastatin, autolytimycin, and non-benzoquinone analogues: potent inhibitors of heat shock protein 90. *J. Org. Chem.* **2010**, *75*, 2820–2835. https://doi.org/10.1021/jo1000109.
2. Wang, C.; Wang, L.; Fan, J.; Sun, K.; Zhu, W. Cytotoxic compounds from the deep-sea sediment-derived *Streptomyces malaysiensis* OUCMDZ-2167. *Chin. J. Org. Chem.* **2017**, *37*, 658–666. https://doi.org/10.6023/cjoc201609021.
3. Caballero, E.; Avendano, C.; Menendez, J. C. Brief total synthesis of the cell cycle inhibitor tryprostatin B and related preparation of its alanine analogue. *J. Org. Chem.* **2003**, *68*, 6944–6951. https://doi.org/10.1021/jo034703l.
4. Ivanova, V.; Laatsch, H.; Kolarova, M.; Aleksieva, K. Structure elucidation of a new natural diketopiperazine from a *Microbispora aerata* strain isolated from Livingston Island, Antarctica. *Nat. Prod. Res.* **2013**, *27*, 164–170. https://doi.org/10.1080/14786419.2012.665911.
5. Yan, Y.M.; Zhu, H.J.; Xiang, B.; Qi, J.J.; Wang, X.L.; Geng, F.N.; Cheng, Y.X. Chemical constituents from *Periplaneta Americana* and their effects on wound healing. *Nat. Prod. Res. Dev.* **2018**, *30*, 591–596. https://doi.org/10.16333/j.1001-6880.2018.4.010.
6. Mehnaz, S.; Saleem, R. S.; Yameen, B.; Pianet, I.; Schnakenburg, G.; Pietraszkiewicz, H.; Valeriote, F.; Josten, M.; Sahl, H. G.; Franzblau, S. G.; Gross, H. Lahorenoic acids A-C, *ortho*-dialkyl-substituted aromatic acids from the biocontrol strain *Pseudomonas aurantiaca* PB-St2. *J. Nat. Prod.* **2013**, *76*, 135–141. https://doi.org/10.1021/np3005166.
7. Ding, G.Z.; Liu, Y.B.; Ma, S.G.; Yu, S.S. Metabolites of *Aspergillus fumigatus*. *China J. Chin. Mater. Med.* **2012**, *37*, 3083–3085.
8. Stark, T.; Hofmann, T. Structures, sensory activity, and dose/response functions of 2,5-diketopiperazines in roasted cocoa nibs (*Theobroma cacao*). *J. Agric. Food Chem.* **2005**, *53*, 7222–7231. https://doi.org/10.1021/jf051313m.

9. Furtado, N.; Pupo, M.T.; Ivone, C.; Campo, V.L.; Duarte, M.; Bastos, J.K. Diketopiperazines produced by an *Aspergillus fumigatus* Brazilian strain. *J. Braz. Chem. Soc*. **2005**, *16*, 1448–1453. https://doi.org/10.1590/S0103-50532005000800026.
10. Isaka, M.; Palasarn, S.; Rachtawee, P.; Vimuttipong, S.; Kongsaeree, P. Unique diketopiperazine dimers from the insect pathogenic fungus *Verticillium hemipterigenum* BCC 1449. *Org. Lett.* **2005**, *7*, 2257–2260. https://doi.org/10.1021/ol0507266.
11. Laville, R.; Nguyen, T.B.; Moriou, C.; Petek, S.; Debitus, C.; Al-Mourabit, A. Marine natural occurring 2,5-diketopiperazines: isolation, synthesis and optical properties. *Heterocycles* **2015**, *90*, 1351–1366. https://doi.org/10.3987/COM-14-S(K)87.
12. Selvakumar, S.; Sivasankaran, D.; Singh, V.K. Enantioselective Henry reaction catalyzed by C_2-symmetric chiral diamine–copper (II) complex. *Org. Biomol. Chem.* **2009**, *7*, 3156–3162. https://doi.org/10.1039/b904254g.
13. Campbell, J.; Lin, Q.; Geske, G. D.; Blackwell, H. E. New and unexpected insights into the modulation of LuxR-type quorum sensing by cyclic dipeptides. *ACS Chem. Biol.* **2009**, *4*, 1051–1059. https://doi.org/10.1021/cb900165y.
14. Zheng, D.; Han, L.; Jiang, Y.; Cao, Y.R.; Liu, J.; Chen, X.; Li, Y.Q.; Huang, X.S. Structure elucidation of four prenylindole derivatives from *Streptomyces* sp. isolated from *Ailuropoda melanoleuca* feces. *Magn. Reson. Chem*. **2013**, *51*, 188–191. https://doi.org/10.1002/mrc.3928.
15. Lee, S.R.; Lee, D.; Park, M.; Lee, J.C.; Park, H.J.; Kang, K.S.; Kim, C.E.; Beemelmanns, C.; Kim, K.H. Absolute configuration and corrected NMR assignment of 17-hydroxycyclooctatin, a fused 5-8-5 tricyclic diterpene. *J. Nat. Prod*. **2020**, *83*, 354–361. https://doi.org/10.1021/acs.jnatprod.9b00837.
16. Zhao, G.S.; Li, S.R.; Wang, Y.Y.; Hao, H.L.; Shen, Y.M.; Lu, C.H. 16,17-Dihydroxycyclooctatin, a new diterpene from *Streptomyce*s sp. LZ35. *Drug. Discov. Ther*. **2013**, *7*, 185–188. https://doi.org/10.5582/ddt.2013.v7.5.185.
17. Wang, G.Y.S.; Graziani, E.; Waters, B.; Pan, W.; Li, X.; McDermott, J.; Meurer, G.; Saxena, G.; Andersen, R.J.; Davies, J. Novel natural products from soil DNA libraries in a streptomycete host. *Org. Lett*. **2000**, *2*, 2401–2404. https://doi.org/10.1021/ol005860z.
18. Matsubara, K.; Sakuda, S.; Kondo, T.; Tanaka, M.; Nishimura, T.; Suzuki, A. Morphological changes in insect BM-N4 cells induced by nocardamine. *Biosci. Biotechnol. Biochem*. **1998**, *62*, 2049–2051. https://doi.org/10.1271/bbb.62.2049.
19. Tao, M.H.; Chen, Y.C.; Wei, X.Y.; Tan, J.W.; Zhang, W.M. Chemical constituents of the endophytic fungus *Phomopsis* sp. A240 isolated from *Taxus chinensis* var. *mairei*. *Helv. Chim. Acta.* **2014**, *97*, 426–430. https://doi.org/10.1002/hlca.201300367.
20. Conti, R.; Chagas, F. O.; Caraballo-Rodriguez, A. M.; Melo, W. G.; do Nascimento, A. M.; Cavalcanti, B. C.; de Moraes, M. O.; Pessoa, C.; Costa-Lotufo, L. V.; Krogh, R.; Andricopulo, A. D.; Lopes, N. P.; Pupo, M. T. Endophytic actinobacteria from the Brazilian medicinal plant *Lychnophora ericoides* mart. and the biological potential of their secondary metabolites. *Chem Biodivers.* **2016**, *13*, 727–736. https://doi.org/10.1002/cbdv.201500225.
21. Wischang, D.; Hartung, J. Parameters for bromination of pyrroles in bromoperoxidase-catalyzed oxidations. *Tetrahedron.* **2011**, *67*, 4048–4054. https://doi.org/10.1016/j.tet.2011.04.010.
22. Dietera, A.; Hamm, A.; Fiedler, H. P.; Goodfellow, M.; Muller, W. E.; Brun, R.; Beil, W.; Bringmann, G. Pyrocoll, an antibiotic, antiparasitic and antitumor compound produced by a novel alkaliphilic *Streptomyces* strain. *J. Antibiot.* **2003**, *56*, 639–646. https://doi.org/10.7164/antibiotics.56.639.
23. Sugiyama, Y.; Oya, A.; Kudo, T.; Hirota, A. Surugapyrone A from *Streptomyces coelicoflavus* strain USF-6280 as a new DPPH radical-scavenger. *J. Antibiot*. **2010**, *63*, 365–369. https://doi.org/10.1038/ja.2010.60.
